# Effects of exercise interventions on executive function in adolescents: a systematic review and three-level meta-analysis of randomized controlled trials

**DOI:** 10.3389/fpsyt.2026.1968917

**Published:** 2026-10-27

**Authors:** Dongying Li, Shixian Li, Min Yao, Sihong Ye, Kun Geng, Liyong Zhou, Lulu Niu

**Affiliations:** 1 School of Physical Education, Yunnan Minzu University, Kunming, China; 2 School of Education, Yunnan Minzu University, Kunming, China; 3 School of Physical Education, Yunnan University, Kunming, China

**Keywords:** adolescents, cognitive flexibility, executive function, exercise intervention, inhibitory control, three-level meta-analysis, working memory

## Abstract

**Objective:**

To estimate the effects of exercise interventions lasting at least 4 weeks on overall executive function and its three domains in adolescents, and to explore differences across population subgroups.

**Methods:**

Nine databases were systematically searched. Individually randomized parallel-group trials of adolescents aged 10–19 years comparing exercise with no additional structured exercise were included. Dependent Hedges’ g estimates were synthesized using three-level models with approximate within-trial covariance and trial-clustered CR2 inference. Risk of bias and evidence certainty were assessed using RoB 2 and GRADE.

**Results:**

Twenty-two independent trials (1,599 unique participants) contributed 27 comparisons and 86 effect sizes. The pooled overall EF effect favored exercise (g = 0.55, 95% CI 0.33 to 0.77; p < 0.001), with substantial total heterogeneity (I² = 84.92%). Domain-specific estimates also favored exercise: inhibitory control (17 trials; g = 0.56, 95% CI 0.28 to 0.84), working memory (10 trials; g = 0.51, 95% CI 0.18 to 0.84), and cognitive flexibility (nine trials; g = 0.43, 95% CI 0.30 to 0.57). The overall estimate was more heavily informed by inhibitory-control scores (47/86) than by working-memory (25/86) or cognitive-flexibility scores (14/86). Correlation, trial-exclusion, risk-of-bias, and task-classification sensitivity analyses retained effects in the same direction. Certainty was low for overall EF, inhibitory control, and working memory, and moderate for cognitive flexibility.

**Conclusion:**

Sustained exercise may improve performance on EF tasks in adolescents, but heterogeneous interventions and controls, overlapping task constructs, and mostly low-certainty evidence limit the generality and real-world interpretation of the pooled effects.

**Systematic review registration:**

https://www.crd.york.ac.uk/PROSPERO/view/CRD420261475826, identifier CRD420261475826

## 1 Introduction

Executive function (EF) comprises inhibitory control, working memory, and cognitive flexibility. These higher-order processes enable individuals to maintain goals, resist distraction, update information, and shift flexibly between rules (1–4). Adolescence involves continuing reorganization of frontostriatal and frontoparietal networks. It is also a sensitive period when trajectories of self-regulation, learning behavior, and mental health increasingly diverge (5, 6). Better self-control during childhood and adolescence is associated with health, economic, and social outcomes in adulthood (7). However, most adolescents worldwide do not achieve recommended levels of physical activity (8, 9). Academic pressure, sedentary behavior, and mental health risks further underscore the need for scalable and low-stigma interventions with physical and cognitive benefits. Clarifying whether long-term exercise produces reliable benefits across EF domains is therefore relevant to developmental cognitive science. It also has practical implications for school physical education, weight management, and youth mental health care.

Previous research provides a strong foundation for this question. Mechanistic studies suggest that exercise may support cognition through brain-derived neurotrophic factor, angiogenesis, cerebral blood flow, synaptic plasticity, and hippocampal structure (10–16). Cognitively engaging open-skill or game-based activities may also train attentional allocation, rule updating, and motor inhibition. Systematic reviews and meta-analyses have generally reported favorable associations between exercise and EF, attention, or academic outcomes in young people (17–24). More recent syntheses have examined cognitively engaging exercise, multilevel effect structures, and adolescent-specific populations (25–30). Collectively, this work established exercise as more than a fitness intervention. It may also offer a cognitive and psychological strategy that can be embedded in educational and clinical settings. Previous evidence further suggests genuine variation by EF domain, exercise modality, and health status.

Despite these advances, existing syntheses have several interrelated methodological limitations. First, some reviews combined children with adolescents or acute exercise with training programs lasting several weeks. These developmental stages and exposure patterns are not fully comparable. Second, a single trial often reports several tasks, conditions, response-time outcomes, and accuracy outcomes. Selecting one outcome or averaging outcomes before a conventional two-level analysis discards information. Conversely, treating every outcome as independent can overstate precision. Third, differences in exercise modality, health status, comparator, and EF measurement are often compressed into one I² estimate. This approach does not distinguish within-study from between-study heterogeneity (31–34). Fourth, unadjusted cluster-randomized trials may underestimate standard errors. Their inclusion introduces unquantifiable bias when intraclass correlation coefficients or design effects are unavailable (35). These concerns do not negate earlier findings. They indicate the need for an updated synthesis that retains dependent effects and represents uncertainty appropriately.

We therefore systematically reviewed English-language, individually randomized trials of exercise programs lasting at least four weeks in adolescents. We estimated effects on overall executive function (EF), inhibitory control, working memory, and cognitive flexibility. Three-level random-effects models accommodated multiple outcomes within trials and distinguished within-trial from between-trial heterogeneity. Exploratory analyses examined participant health status, mean age, intervention duration, weekly frequency, and session duration. Sensitivity analyses assessed the influence of individual trials, high-risk results, task classification, and assumed correlations. Funnel-plot analyses and GRADE assessments informed judgments about small-study effects and evidence certainty. This approach distinguishes independent trials from dependent effect sizes and supports cautious interpretation of the pooled estimates without implying a superior exercise prescription.

## 2 Methods

### 2.1 Study design, reporting guidelines, and registration

This systematic review and three-level meta-analysis of randomized controlled trials (RCTs) followed the PRISMA 2020 statement (36). Methods were also informed by the Cochrane Handbook for Systematic Reviews of Interventions (35). Protocol registration, https://www.crd.york.ac.uk/PROSPERO/view/CRD420261475826.

### 2.2 Eligibility criteria

Eligibility criteria were defined according to the PICOS framework:

(1) Participants: Adolescents aged 10–19 years, consistent with the World Health Organization (WHO) definition of adolescence, regardless of health status. (2) Interventions: Structured exercise programs lasting at least 4 weeks, with no restriction on exercise modality. (3) Comparators: No additional structured exercise prescribed by the investigators; usual activities, waitlist, non-exercise health education, or regular school activities, including physical education, were permitted.

(4) Outcomes: At least one measure of the three core executive function domains—inhibitory control, working memory, or cognitive flexibility—with sufficient data to calculate a standardized effect size. (5) Study design: Peer-reviewed, English-language, parallel-group randomized controlled trials.

Studies were excluded if they involved acute or <4-week exercise interventions, crossover or nonrandomized designs, participants outside the 10–19-year age range without separately extractable adolescent data, an additional structured exercise intervention prescribed by the investigators in the control group, outcomes unrelated to the three core executive-function domains, duplicate reports, or insufficient statistical data. These decisions and full-text exclusion reasons are documented in **Figure 1** and **Supplementary Table S2**.

**Figure 1:**
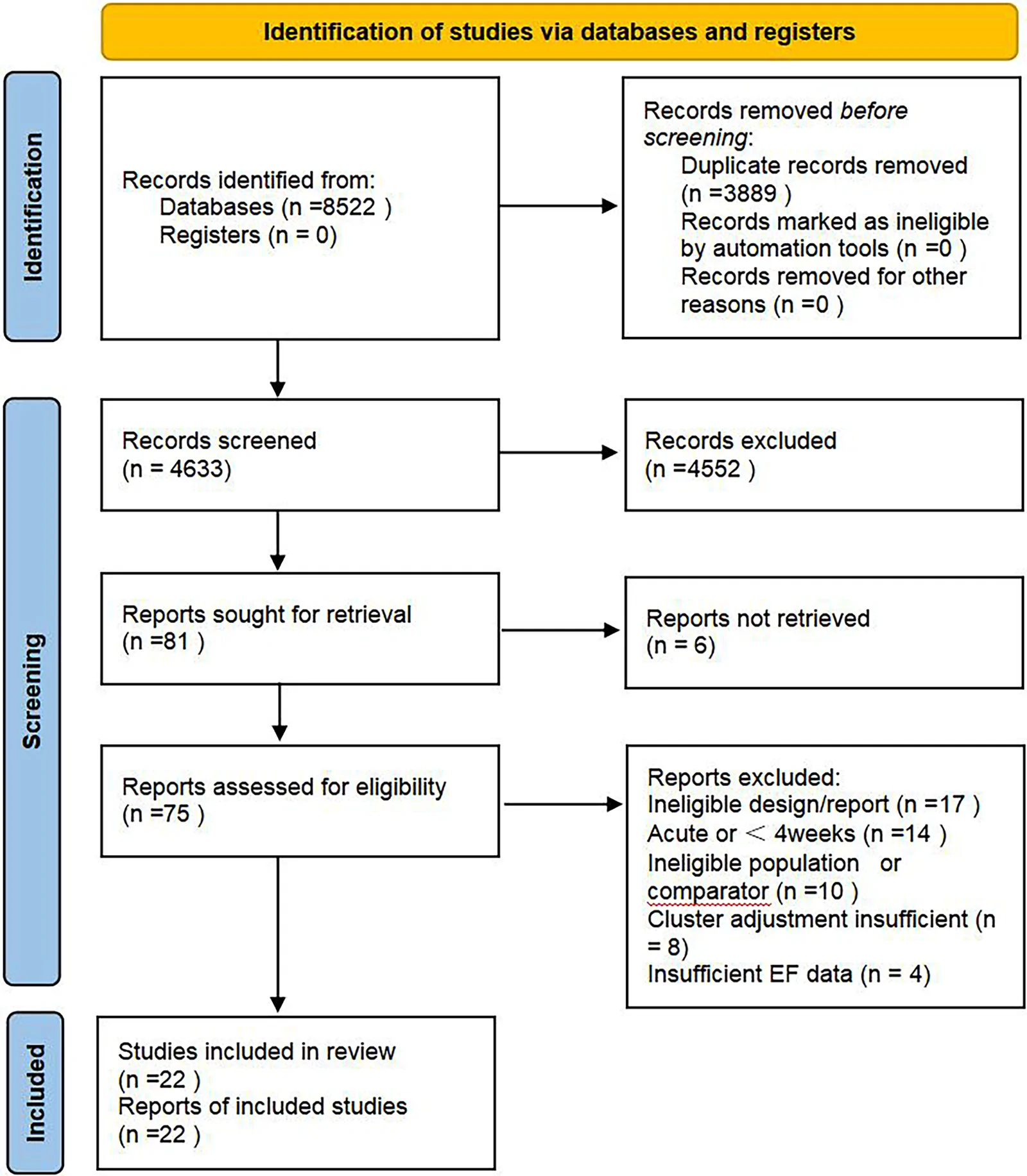
PRISMA 2020 flow diagram of study selection.

### 2.3 Search strategy

PubMed, Web of Science, Embase, the Cochrane Library, Scopus, CINAHL, SPORTDiscus, PsycINFO, and ERIC were searched from inception through 29 September 2026. Database-specific strategies are reproduced in **Supplementary Table S1**. Two reviewers independently screened titles/abstracts and full texts, resolving disagreement by discussion or adjudication. Records and reports were tracked separately in the PRISMA flow diagram; a report could be excluded at full text for the first applicable reason.

### 2.4 Study selection and data extraction

Two reviewers (DL and LN) independently removed duplicates, screened titles and abstracts, and assessed full texts. Disagreements were resolved through discussion or adjudication by a third reviewer (SL). A prespecified form captured author, year, country or region, health status, age, randomized sample size, and comparator. It also captured exercise modality, frequency, intensity, session duration, total weeks, EF task, outcome direction, and preintervention and postintervention means and standard deviations. When one study included several eligible exercise arms, each comparison was retained as a separate study arm. Shared control participants were counted once in unique-participant totals and their full reported sample size was retained for effect-size calculation; the control group was not split. Multiple tasks within a comparison contributed distinct, statistically dependent effect sizes. Participant counts across EF domains can overlap and must not be summed.

### 2.5 Executive-function domains and outcome coding

We assigned each extracted score to the EF domain most directly targeted by its task and scoring metric, with score direction harmonized so positive g favored exercise. Stroop, flanker, Go/No-Go, and related conflict scores were coded as inhibitory control; N-back, Sternberg, and span scores as working memory; and set-shifting, task-switching, Wisconsin Card Sorting Test, and Trail Making Test B scores as cognitive flexibility. This pragmatic classification is not a claim that any task is construct-pure. **Supplementary Table S10** provides the task, exact extracted score, coding basis, and ambiguity flag for all 86 effects. Because Posner cueing scores also reflect spatial attention, Digit Span total may reflect storage rather than manipulation, and raw Trail Making Test B time includes visual search and motor speed, we repeated the synthesis after excluding only these 11 affected scores (six, three, and two, respectively), retaining other eligible results from their trials.

### 2.6 Risk-of-bias assessment

Two reviewers (DL and LN) independently assessed risk of bias using the Cochrane RoB 2 tool (35). Judgments concerned the effect of assignment to intervention and covered randomization, deviations from intended interventions, missing outcome data, outcome measurement, and selective reporting. Overall risk was classified as low risk, some concerns, or high risk. Disagreements were resolved through discussion or adjudication by a third reviewer (SL).

### 2.7 Effect-size calculation

For each arm and outcome, change was defined as postintervention minus preintervention mean. When change-score SD was not reported, it was estimated as $SD_{\Delta }=\sqrt{SD_{\text{pre}}^{2}+SD_{\text{post}}^{2}-2r\;SD_{\text{pre}}SD_{\text{post}}}$, using r = 0.50 in the primary analysis. Hedges’ g was the small-sample-corrected standardized difference between exercise and control mean changes, standardized by the pooled change-score SD. Sampling variances used the metafor implementation of the standardized mean difference (escalc, measure = “SMD”, correct = TRUE, vtype = “LS”) (37). Thus, this analysis uses a pooled-change-SD denominator; it is not the pretest-SD-standardized Morris dppc2 estimator (38). Lower-is-better scales were reversed before synthesis.

We constructed an approximate block-diagonal sampling-covariance matrix within each independent trial using metafor::vcalc, specifying trial, task/score identifier, exercise-arm identifier, control-arm identifier, the full arm sample sizes, and an assumed within-trial outcome correlation ρ = 0.50 (37). This representation allows covariances both among scores from the same participants and among comparisons with a shared control; it does not recover unknown empirical correlations. A three-level random-effects model (rma.mv, REML; effect sizes nested within trial) then estimated a pooled mean and level-2 and level-3 variance components. Confidence intervals and p-values used CR2 cluster-robust inference with the independent trial as cluster, to reduce reliance on a fully correct working covariance matrix when the number of trials is limited (39). We present level-specific τ² and I², and total I² (40); a variance estimate at or near the zero boundary is not evidence that outcomes are truly homogeneous.

The overall model pooled all eligible EF scores. Because the number of extracted scores differs by domain, this is an outcome-weighted average over the observed task mix and must not be read as equal improvement in all three EF domains. Separate models were fitted for inhibitory control, working memory, and cognitive flexibility. All analyses were performed in R with metafor and clubSandwich (37, 39); the analysis code is available from the corresponding author on reasonable request.

### 2.8 Secondary and sensitivity analyses

We assessed sensitivity to the assumed correlations by varying one parameter at a time. The within-trial outcome correlation was set to ρ = 0.30 or 0.70 while the pre–post correlation remained r = 0.50. The pre–post correlation was then set to r = 0.30 or 0.70 while ρ remained 0.50. Additional analyses removed all effect sizes from one independent trial at a time, excluded results rated at high overall risk of bias by RoB 2 for the corresponding outcome, and excluded the 11 scores with ambiguous EF-domain attribution.

Exploratory subgroup analyses considered healthy adolescents, adolescents with overweight or obesity, and adolescents with ADHD separately. Trials involving subthreshold depression or elevated suicide risk were not combined with ADHD into a single clinical subgroup. Four separate univariable meta-regressions examined trial-level mean age, intervention duration in weeks, weekly exercise frequency, and session duration. We reported both unadjusted and Benjamini–Hochberg-adjusted p-values for these four moderator tests (41). To assess small-study effects, correlated scores were aggregated to one estimate per independent trial before constructing funnel plots and conducting exploratory Egger-type and Begg tests (42–44). These tests were interpreted cautiously, particularly when fewer than ten trials contributed to an outcome.

### 2.9 Certainty of the evidence

The certainty of the evidence for overall executive function and each executive-function domain was assessed using the GRADE approach (45). Evidence from randomized trials was initially rated as high certainty and was downgraded, where appropriate, for risk of bias, inconsistency, indirectness, imprecision, or publication bias. The resulting certainty of evidence was classified as high, moderate, low, or very low. GRADE ratings reflect overall confidence in the estimated effect and should not be interpreted solely according to statistical significance or a single p value.

## 3 Results

### 3.1 Study selection

The updated searches identified 8,522 records. After 3,889 duplicates were removed, 4,633 records underwent title/abstract screening and 4,552 were excluded. Of 81 reports sought, six could not be obtained; 75 full texts were assessed and 53 were excluded (ineligible design, n = 16; acute or <4-week intervention, n = 14; cluster randomization without usable adjustment, n = 8; ineligible active-exercise comparator, n = 7; insufficient or ineligible EF data, n = 4; ineligible age, n = 3; duplicate/secondary publication, n = 1). Twenty-two independent RCTs entered the synthesis (**Figure 1**; **Supplementary Table S2**).

### 3.2 Study characteristics

The 22 independent RCTs were published between 2015 and 2026 and were conducted across nine countries. Eleven were conducted in China: six in mainland China (46–51), four in Taiwan, China (52–55), and one in Hong Kong, China (56). Four were conducted in South Korea (57–60). One trial each was conducted in Tunisia (61), Spain (62), India (63), Brazil (64), Belgium (65), Türkiye (66), and Denmark (67). The trials included 1,599 unique analyzed participants: 866 in exercise groups and 733 in control groups. Shared-control participants were counted only once. Twelve trials involved generally healthy adolescents, five involved adolescents with overweight or obesity, three involved adolescents with ADHD, one involved adolescents with subthreshold depression, and one involved adolescents at elevated suicide risk. Interventions lasted 8–78 weeks and included high-intensity interval training, moderate-intensity continuous training, aerobic and resistance exercise, and cognitively engaging movement games. Other programs involved basketball, badminton, running, rope skipping, taekwondo, swimming, football juggling, yoga, small-sided games, coordination training, or classroom cycling. The 22 RCTs yielded 27 exercise–control comparisons and 86 EF scores. Detailed trial and arm characteristics are provided in **Supplementary Table S3**.

### 3.3 Risk of bias

RoB 2 assessments covered 45 trial-arm–domain results. Eight (17.8%) were rated low risk, 33 (73.3%) raised some concerns, and four (8.9%) were rated high risk. Some concerns were most frequent for the randomization process and selection of the reported result, whereas outcome measurement was predominantly rated low risk. High-risk domain judgments occurred in deviations from intended interventions (**Figures 2**, **3**).

**Figure 2:**
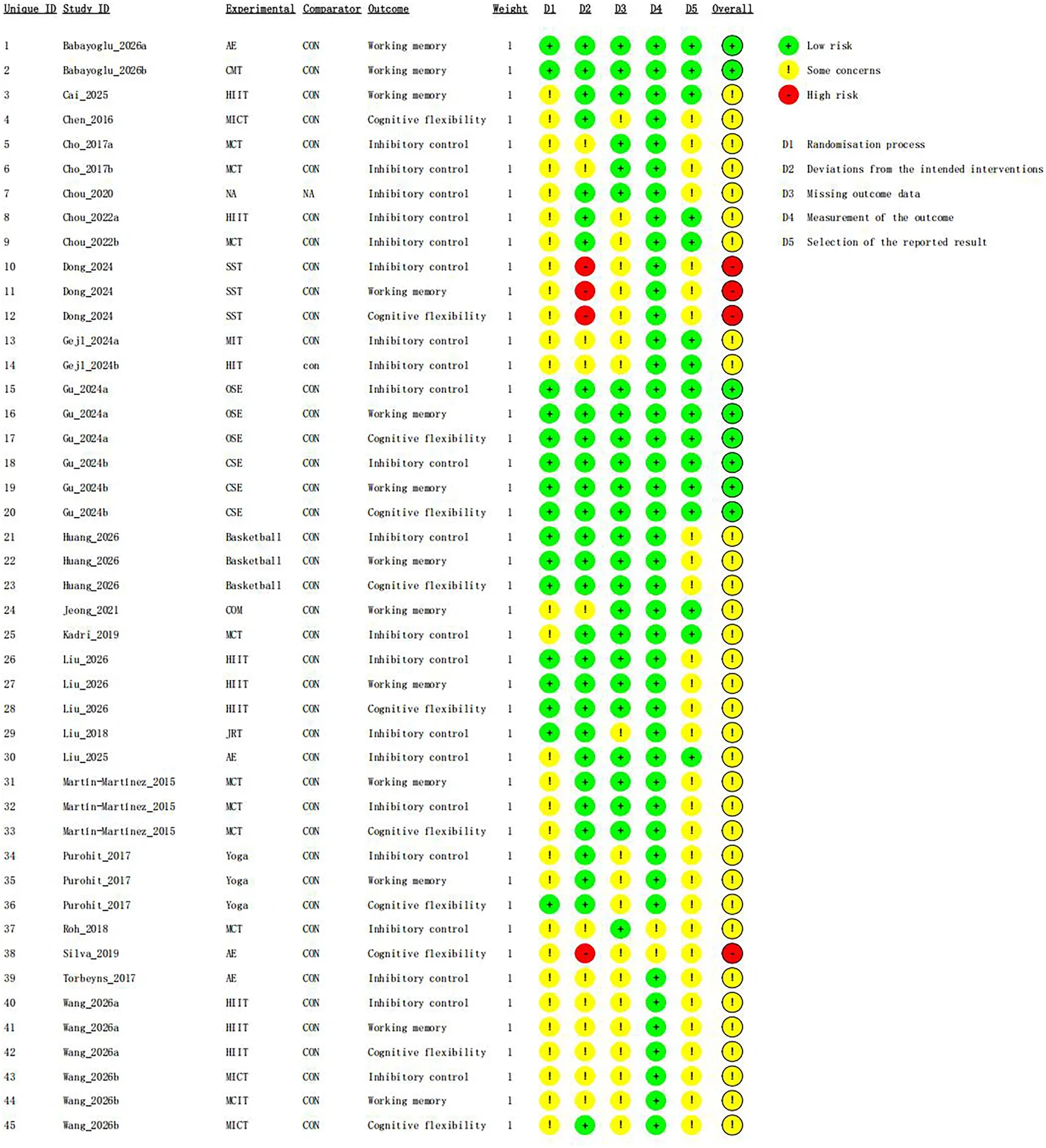
Traffic-light plot of RoB 2 judgments for included comparisons.

**Figure 3:**
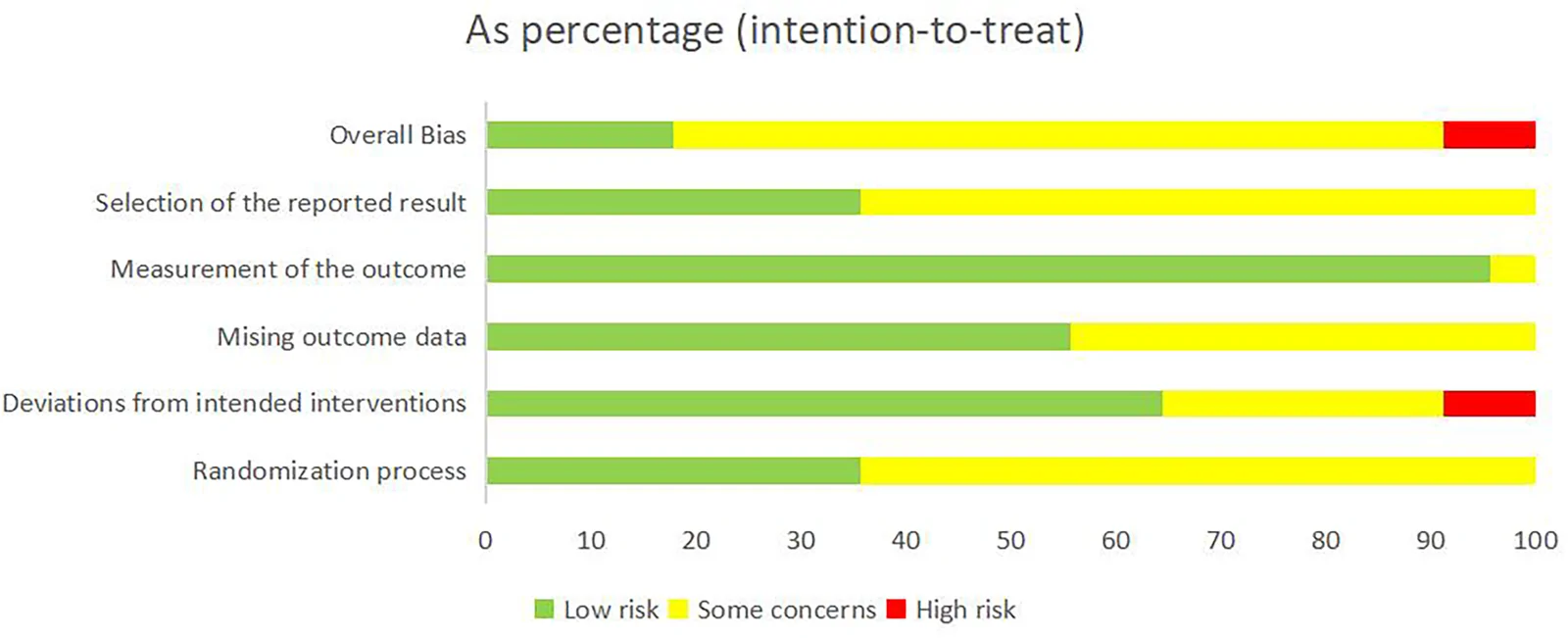
Distribution of RoB 2 judgments by domain for the intention-to-treat effect.

### 3.4 Overall and domain-specific three-level meta-analyses

The overall EF analysis gave g = 0.55 (95% CI 0.33 to 0.77; p < 0.001) across 22 RCTs, 27 comparisons, and 86 effects. The level-2 and level-3 variance estimates were 0.05 and 0.16; their I² components were 20.03% and 64.89% (total I² = 84.92%). This substantial heterogeneity means the mean is not a prediction of benefit in every setting.

The inhibitory-control model included 17 RCTs, 21 comparisons, and 47 effects (g = 0.56, 95% CI 0.28 to 0.84; p < 0.001; total I² = 84.93%). Working memory included ten RCTs, 13 comparisons, and 25 effects (g = 0.51, 95% CI 0.18 to 0.84; p = 0.007; total I² = 80.67%). Cognitive flexibility included nine RCTs, 11 comparisons, and 14 effects (g = 0.43, 95% CI 0.30 to 0.57; p < 0.001); its fitted variance components were at the near-zero boundary and should not be overinterpreted as proof of no heterogeneity. The 47, 25, and 14 effects sum to the overall 86 effects, but neither score counts nor participant counts are balanced across domains. Results are in **Table 1** and **Supplementary Figures S1**–**S4**.

**Table 1:** Three-level random-effects meta-analysis of exercise interventions and executive function.

Outcome	RCTs, n	Comparisons, n	Effect sizes, n	Hedges’ g	95% CI	p-value	T^2^ Level 2	T^2^ Level 3	I^2^ Level 2 (%)	I^2^ Level 3 (%)
Overall EF	22	27	86	0.55	0.33 to 0.77	<0.001	0.05	0.16	20.03	64.89
Inhibitory control	17	21	47	0.56	0.28 to 0.84	<0.001	0.03	0.22	11.55	73.38
Working memory	10	13	25	0.51	0.18 to 0.84	0.007	<0.01	0.16	<0.01	80.67
Cognitive flexibility	9	11	14	0.43	0.30 to 0.57	<0.001	<0.01	<0.01	<0.01	<0.01

RCTs are independent randomized trials; comparisons are exercise–control contrasts; effect sizes are extracted scores. Hedges’ g is the small-sample-corrected standardized difference in mean pre–post changes. Positive values favor exercise. Level 2 represents residual within-trial heterogeneity and level 3 between-trial heterogeneity. Values shown as <0.01 are boundary-near estimates, not a claim of zero biological variation. Confidence intervals and p-values use trial-clustered CR2 inference.

### 3.5 Subgroup analyses by health status

The healthy subgroup comprised 12 RCTs and 36 effects (g = 0.46, 95% CI 0.23 to 0.69; p = 0.001); the overweight/obesity subgroup comprised five RCTs and 26 effects (g = 0.72, 95% CI 0.16 to 1.27; p = 0.023); and the ADHD subgroup comprised three RCTs and ten effects (g = 1.14, 95% CI −1.41 to 3.70; p = 0.192). The three-group difference was not statistically supported (CR2/HTZ p = 0.530), and the ADHD estimate was very imprecise. The subthreshold-depression and elevated-suicide-risk trials contributed to overall EF but not to these three subgroups. These analyses are descriptive and exploratory, not evidence of a superior population or exercise prescription (**Supplementary Table S4**; **Supplementary Figure S5**).

### 3.6 Sensitivity analyses

Leaving out all effects from one independent RCT at a time yielded overall g estimates from 0.49 to 0.58; corresponding ranges were 0.48–0.60 for inhibitory control, 0.42–0.59 for working memory, and 0.41–0.47 for cognitive flexibility. All retained the same direction and statistical significance (**Supplementary Tables S5**–**S8**; **Supplementary Figures S6**–**S9**). After excluding outcome-specific high-risk results, overall EF remained g = 0.55 (95% CI 0.32 to 0.78; 20 RCTs), working memory g = 0.59 (0.29 to 0.89; nine RCTs), and cognitive flexibility g = 0.44 (0.29 to 0.59; seven RCTs); inhibitory control also remained positive (g = 0.56, 0.26 to 0.87; 16 RCTs) (**Supplementary Table S9**; **Supplementary Figures S10**–**S13**).

Excluding the 11 construct-ambiguous scores, not their entire source trials, left 75 effects from 20 trials and an overall estimate of g = 0.53 (95% CI 0.30 to 0.75; p < 0.001). The domain-specific estimates remained positive: inhibitory control g = 0.59 (0.29 to 0.90), working memory g = 0.42 (0.12 to 0.71), and cognitive flexibility g = 0.45 (0.28 to 0.62) (**Supplementary Table S10**; **Supplementary Figures S14**–**S17**).

At fixed r = 0.50, varying the assumed within-trial outcome correlation ρ from 0.30 to 0.70 changed overall g only from 0.56 (0.33 to 0.78) to 0.55 (0.34 to 0.76). At fixed ρ = 0.50, varying the assumed pre–post correlation r from 0.30 to 0.70 changed overall g from 0.47 (0.29 to 0.66) to 0.69 (0.42 to 0.96). All overall p-values were <0.001, but the latter range shows that the estimated magnitude depends materially on r. Corresponding analyses for each domain are in **Supplementary Table S11**; **Supplementary Figures S18**–**S21**.

### 3.7 Small-study effects and publication bias

Trial-level aggregated funnel plots and exploratory tests did not detect asymmetry for overall EF (22 RCTs; Egger-type p = 0.823; Begg p = 0.288) or the three domains. The inhibitory-control tests were p = 0.417 and 0.440; working-memory tests p = 0.484 and 0.381; cognitive-flexibility tests p = 0.770 and 0.477, respectively (**Supplementary Figures S22**–**S25**). With ten or fewer trials in two domains and heterogeneous effects, these nonsignificant tests cannot exclude publication bias or small-study effects.

### 3.8 Univariate meta-regression

In univariable models, mean age had an unadjusted slope of β = −0.102 Hedges’ g per year (95% CI −0.190 to −0.015; p = 0.028), but its Benjamini–Hochberg-adjusted p-value was 0.111. Slopes for intervention weeks (β = 0.023; adjusted p = 0.330), weekly frequency (β = −0.032; adjusted p = 0.987), and session duration (β approximately 0.000; adjusted p = 0.987) were not supported. None of the four moderators remained statistically significant after adjustment. These aggregate-data analyses are underpowered for modest effects and cannot establish causal dose–response relationships (**Supplementary Table S12**; **Supplementary Figure S26**).

### 3.9 Certainty of evidence

GRADE certainty was low for overall EF and inhibitory control because of serious risk of bias and inconsistency; working memory was also low for these reasons. Cognitive flexibility was rated moderate after downgrading for risk of bias. These ratings qualify the confidence placed in each estimated effect, regardless of its p-value (**Table 2**).

**Table 2:** GRADE summary of findings.

Outcome	K	Study design	Risk of bias	Inconsistency	Indirectness	Imprecision	Other considerations	N(E/C)	SMD [95% CI]	Certainty	Importance
Overall Executive function	22	RCT	serious	serious	not serious	not serious	none	866/733	0.55 (0.33- 0.77)	⨁⨁◯◯ Low	CRITICAL
Inhibitory control	17	RCT	serious	serious	not serious	not serious	none	652/551	0.56 (0.28- 0.84)	⨁⨁◯◯ Low	CRITICAL
Working memory	10	RCT	serious	serious	not serious	not serious	none	526/433	0.51 (0.18- 0.84)	⨁⨁◯◯ Low	CRITICAL
Cognitive flexibility	9	RCT	serious	not serious	not serious	not serious	none	382/321	0.43 (0.30- 0.57)	⨁⨁⨁◯ Moderate	CRITICAL

N(E/C) gives unique analyzed participants in exercise/control groups for each outcome; a participant may contribute to more than one EF domain, so domain counts should not be summed. SMD is Hedges’ g. Overall and domain-specific certainty judgments are outcome-specific.

## 4 Discussion

Across 22 independent RCTs, exercise interventions were associated with better average overall EF task performance than control conditions (Hedges’ g = 0.55). Pooled estimates for inhibitory control, working memory, and cognitive flexibility were also positive. However, total heterogeneity was substantial for overall EF (I² = 84.92%), inhibitory control (84.93%), and working memory (80.67%). GRADE certainty was low for these three outcomes and moderate for cognitive flexibility. The pooled estimates should therefore be interpreted as average effects across the included trials, not as evidence of uniform benefit across adolescents or settings.

The marked heterogeneity is likely to have more than one source. For overall EF, the level-3 component accounted for 64.89% of total variability, indicating considerable differences between trials. The trials varied in participant health status and baseline characteristics, exercise content and cognitive demands, intervention dose, comparator conditions, and EF assessments. Exploratory analyses did not identify a reliable explanation for this variability: the difference among the healthy, overweight/obesity, and ADHD subgroups was not statistically supported (p = 0.530), and none of the four meta-regression moderators remained significant after adjustment for multiple testing. The ADHD estimate was especially imprecise because it was based on three trials. These findings should not be interpreted as evidence that the populations respond equally, nor do they establish a superior exercise modality, intensity, frequency, or duration. Control conditions ranged from non-exercise activities to usual physical education. In some school-based trials, both groups continued regular physical education, while only the intervention group received additional structured exercise. This variation may have contributed to heterogeneity, but comparator type was not tested as a separate moderator.

The positive direction of the findings is broadly consistent with earlier reviews of physical activity and cognition in young people (17, 20, 22–25, 30) Direct comparisons of pooled magnitudes are nevertheless difficult because reviews differ in age ranges, intervention duration, eligible tasks, participant health status, and treatment of multiple outcomes. The present domain estimates should also not be treated as a ranking of EF components. Exercise activities may involve stopping responses, maintaining instructions, updating information, or switching rules, but the included trials did not isolate these demands experimentally. Differences among domain estimates could reflect intervention content, task properties, or the different sets of trials contributing to each model, rather than greater biological sensitivity of one EF component.

The interpretation of “overall EF” also depends on the available task mix. Of the 86 extracted scores, 47 assessed inhibitory control, 25 working memory, and 14 cognitive flexibility. The overall estimate is therefore an average across the observed scores, not an equally weighted measure of three distinct underlying abilities. Individual tasks are not construct-pure: Posner cueing also reflects attentional orienting, Digit Span total may emphasize storage, and Trail Making Test B time includes visual search and motor speed. Excluding 11 scores with particularly uncertain domain attribution left a positive overall estimate (g = 0.53), indicating that these scores alone did not account for the main finding. Nonetheless, neither this sensitivity analysis nor domain coding removes measurement overlap. Moreover, improvement on laboratory EF tasks does not by itself demonstrate better academic achievement, classroom behavior, self-regulation, or daily functioning, even though these functions are relevant to adolescent development (1, 7).

Retaining multiple scores from the same participants avoided selecting one outcome arbitrarily, but it required explicit treatment of dependence. The three-level model separated within-trial and between-trial variability, while the approximate sampling-covariance matrix represented shared participants and shared controls. Trial-clustered CR2 inference reduced reliance on a perfectly specified working covariance matrix (31–34, 37, 39). These procedures cannot recover correlations that the original trials did not report. Varying the assumed correlation among outcomes had little effect on the overall estimate, whereas changing the assumed pre–post correlation from 0.30 to 0.70 shifted g from 0.47 to 0.69. The direction of the finding was stable, but its precise magnitude remains assumption-dependent. Leave-one-trial-out, high-risk-result exclusion, and task-coding analyses likewise retained positive estimates; they do not eliminate the substantial heterogeneity or the risk-of-bias concerns.

Several interacting pathways could plausibly link sustained exercise with EF performance, including cognitive engagement, social interaction, mood, fitness, and neurobiological adaptation (10–16). These mechanisms were not isolated in the included trials and cannot be established by the present meta-analysis. Most RoB 2 results raised at least some concerns, and nonsignificant funnel-plot tests cannot exclude small-study effects, particularly in the smaller domain-specific evidence sets. Taken together, the evidence supports a possible average benefit of additional exercise for measured EF task performance under the studied conditions. It does not yet support a specific exercise prescription or demonstrate that the observed test-score changes translate into meaningful, lasting improvements in adolescents’ everyday functioning.

## 5 Strengths and limitations

This review has several methodological strengths. The synthesis distinguished independent trials, exercise–control comparisons, and dependent effect sizes. Risk of bias was assessed for each trial-arm–EF-domain result, and task-to-domain assignments were documented. The three-level model incorporated an approximate sampling-covariance structure, with trial-clustered CR2 inference. Sensitivity analyses examined the influence of individual trials, high-risk results, task classification, and assumptions about pre–post and cross-outcome correlations.

Several limitations warrant caution. Restricting eligibility to English-language, peer-reviewed full-text reports may have excluded relevant evidence. Four reports without usable EF data and eight cluster-randomized trials without sufficient information to account for clustering could not be synthesized. Empirical pre–post and cross-outcome correlations were generally unavailable, requiring assumed values. Varying the assumed pre–post correlation changed the pooled effect magnitude, and the approximate covariance structure and CR2 inference cannot remove all uncertainty arising from dependent outcomes.

Some EF domains and subgroup analyses included few independent trials, limiting the precision of estimates and the power to detect differences. Univariable trial-level meta-regressions also remain susceptible to confounding and ecological bias. Overlapping cognitive demands across EF tasks complicate domain-specific interpretation, while changes in laboratory task scores may not translate into everyday benefits. Comparator conditions varied, including non-exercise activities and ordinary physical education; comparator type was not examined in a separate quantitative analysis. Together with substantial heterogeneity and mostly low GRADE certainty, these limitations preclude recommendations for a specific exercise prescription or claims of universal benefit.

## 6 Directions for future research

Future individually randomized trials should pre-register one clearly defined primary EF score per domain; report randomization and concealment, attrition, adherence, analyzed group sizes, and both pre/post and change-score dispersion; and provide correlations needed for multi-outcome synthesis. Shared-control multi-arm studies should identify arm overlap explicitly. Trials should distinguish added exercise from background physical education, measure exercise dose consistently, and include direct academic, self-regulation, or daily-function outcomes alongside task measures. Larger, better reported trials would permit more credible moderator and comparator-specific analyses.

## 7 Conclusion

Sustained exercise was associated with better performance on the EF scores included in 22 adolescent randomized trials. The pooled overall estimate was positive, as were estimates for inhibitory control, working memory, and cognitive flexibility, but the overall estimate is weighted by the available task mix and should not be interpreted as balanced benefit across domains. Substantial heterogeneity, outcome dependence, differing comparators, and mostly low-certainty evidence require cautious interpretation. Present data do not establish a universally effective or superior exercise prescription, nor do they show that test-score changes translate to meaningful daily-life improvement.

## Data Availability

The original contributions presented in the study are included in the article/**Supplementary Material**. Further inquiries can be directed to the corresponding author.
