# Supplementary material for: Effects of exercise interventions on executive function in adolescents: a systematic review and three-level meta-analysis of randomized controlled trials
Source: Front Psychiatry. 2026 Oct 27;17:1968917. doi: 10.3389/fpsyt.2026.1968917 (PMC13748318; doi:10.3389/fpsyt.2026.1968917)
Supplement: Supplementary file 1 [file DataSheet1.pdf]

Supplementary Material

1. Search strategy

The exact database-specific search strategies used for this review are presented below. Nine electronic databases were searched from inception to 29 September 2026: PubMed, Embase, Web of Science Core Collection, Scopus, the Cochrane Central Register of Controlled Trials (CENTRAL), CINAHL via EBSCO, SPORTDiscus via EBSCO, APA PsycINFO, and ERIC. Controlled vocabulary and free-text terms covered adolescents, exercise or physical activity, executive function and its core domains (inhibitory control, working memory, and cognitive flexibility), and randomised or controlled clinical trials. No restrictions were applied regarding the health status of adolescents.

Table S1. Database-specific search strategies

Database	Search strategy	Amount
PubMed		
#1	("Child"[MeSH Terms]) OR ("Adolescent"[MeSH Terms])	3,665,749
#2	(child*[Title/Abstract]) OR (adolescen*[Title/Abstract]) OR (youth[Title/Abstract]) OR (teen*[Title/Abstract]) OR (pediatric[Title/Abstract]) OR (paediatric[Title/Abstract])	2,463,633
#3	#1 OR #2	4,470,753
#4	("Exercise"[MeSH Terms]) OR ("Exercise Therapy"[MeSH Terms]) OR ("Physical Education and Training"[MeSH Terms]) OR ("Sports"[MeSH Terms])	451,797
#5	(exercise[Title/Abstract]) OR ("physical activity"[Title/Abstract]) OR ("exercise training"[Title/Abstract]) OR ("physical training"[Title/Abstract]) OR (sport*[Title/Abstract]) OR ("physical education"[Title/Abstract])	644,235
#6	#4 OR #5	821,805
#7	("Executive Function"[MeSH Terms]) OR ("Memory, Short-Term"[MeSH Terms]) OR ("Inhibition, Psychological"[MeSH Terms]) OR ("Cognitive Flexibility"[MeSH Terms])	65,038
#8	("executive function*"[Title/Abstract]) OR ("working memory"[Title/Abstract]) OR ("inhibitory control"[Title/Abstract]) OR ("cognitive flexibility"[Title/Abstract]) OR ("cognitive control"[Title/Abstract])	106,491
#9	#7 OR #8	131,211
#10	("Randomized Controlled Trial"[Publication Type]) OR (randomized[Title/Abstract]) OR (randomised[Title/Abstract]) OR (trial[Title/Abstract])	1,654,775
#11	#3 AND #6 AND #9 AND #10	588
Web of Science		

#1	(child* OR adolescen* OR youth* OR teen* OR pediatric OR paediatric OR "school-aged")	942,677
#2	(exercise* OR "physical activity" OR "exercise training" OR "physical training" OR sport* OR "physical education")	881,019
#3	("executive function*" OR "executive control" OR "working memory" OR "inhibitory control" OR "cognitive flexibility" OR "cognitive control")	61,510
#4	(randomized OR randomised OR randomly OR trial OR "controlled trial" OR "cluster random*" OR RCT)	1,182,099
#5	#1 AND #2 AND #3 AND #4	544
Embase		
#1	'child'/exp OR 'child' OR 'adolescent'/exp OR 'adolescent'	5,595,150
#2	child*:ti,ab OR adolescen*:ti,ab OR youth*:ti,ab OR teen*:ti,ab OR pediatric*:ti,ab OR paediatric*:ti,ab OR schoolchild*:ti,ab	3,357,281
#3	#1 OR #2	6,179,407
#4	exercise'/exp OR 'physical activity'/exp OR 'exercise therapy'/exp OR 'exercise training'/exp OR 'sport'/exp OR 'physical education'/exp OR 'aerobic exercise'/exp OR 'resistance training'/exp	1,319,624
#5	exercise*:ti,ab OR 'physical activity':ti,ab OR 'exercise training':ti,ab OR 'physical training':ti,ab OR 'aerobic exercise':ti,ab OR 'aerobic training':ti,ab OR 'resistance training':ti,ab OR 'strength training':ti,ab OR 'interval training':ti,ab OR 'high intensity interval training':ti,ab OR hiit:ti,ab OR 'coordination training':ti,ab OR 'cognitively engaging exercise':ti,ab OR sport*:ti,ab OR 'physical education':ti,ab OR running:ti,ab OR cycling:ti,ab OR swimming:ti,ab OR dance:ti,ab OR yoga:ti,ab	1,173,903
#6	#4 OR #5	1,747,373
#7	'executive function'/exp OR 'working memory'/exp OR 'response inhibition'/exp OR 'cognitive flexibility'/exp	136,588
#8	executive function':ti,ab OR 'executive functions':ti,ab OR 'executive control':ti,ab OR 'cognitive control':ti,ab OR 'working memory':ti,ab OR 'inhibitory control':ti,ab OR 'response inhibition':ti,ab OR 'cognitive flexibility':ti,ab OR 'task switching':ti,ab OR 'set shifting':ti,ab OR stroop:ti,ab OR flanker:ti,ab OR 'go/no-go':ti,ab OR 'n-back':ti,ab	158,874
#9	#7 OR #8	197,884
#10	randomized controlled trial'/exp OR 'controlled clinical trial'/exp OR randomized:ti,ab OR randomised:ti,ab OR randomly:ti,ab OR trial:ti,ab OR 'controlled trial':ti,ab OR 'cluster randomized':ti,ab OR 'cluster randomised':ti,ab OR crossover:ti,ab	3,273,211

#11	#3 AND #6 AND #9 AND #10	1,155
Cochrane Library		
#1	Child OR Adolescent OR Children OR Adolescents	351979
#2	child* OR adolescen* OR youth* OR teen* OR pediatric* OR paediatric* OR schoolchild* OR "school aged"	399915
#3	#1 OR #2	399915
#4	Exercise OR "Exercise Therapy" OR "Physical Activity" OR Sport	198483
#5	exercise* OR training OR "physical training" OR "physical activity" OR "exercise training" OR "aerobic exercise" OR "aerobic training" OR "resistance training" OR "strength training" OR "interval training" OR "high intensity interval training" OR HIIT OR "coordination training" OR "physical education" OR sport* OR running OR cycling OR swimming OR dance OR yoga	330287
#6	#4 OR #5	330287
#7	Executive Function OR "Working Memory" OR "Inhibitory Control" OR "Cognitive Flexibility"	17472
#8	executive function OR "executive functions" OR "executive functioning" OR "executive control" OR "cognitive control" OR "working memory" OR "inhibitory control" OR "response inhibition" OR "cognitive flexibility" OR "task switching" OR "set shifting" OR Stroop OR flanker OR "go/no-go" OR "go no go" OR "n-back" OR "n back"	23833
#9	#7 OR #8	23833
#10	randomized OR randomised OR randomly OR random* OR trial* OR "controlled trial" OR "randomized controlled trial" OR "randomised controlled trial" OR "cluster randomized" OR "cluster randomised" OR crossover OR "cross-over"	2452318
#11	#3 AND #6 AND #9 AND #10	2473
Scopus		
#1	TITLE-ABS-KEY(child OR children OR adolescent OR adolescents OR adolescence OR youth OR youths OR teen OR teens OR teenager OR teenagers OR pediatric OR paediatric OR "school aged" OR schoolchild OR schoolchildren)	5919975
#2	TITLE-ABS-KEY (exercise* OR training OR "physical training" OR "physical activity" OR "exercise training" OR "aerobic training" OR "resistance training" OR "strength training" OR "interval training" OR "high intensity interval training" OR HIIT OR "coordination training" OR sport* OR "physical education")	3605089

#3	TITLE-ABS-KEY<br>("executive function" OR "executive functions" OR "executive control" OR "cognitive control" OR "working memory" OR "inhibitory control" OR "response inhibition" OR "cognitive flexibility" OR "task switching" OR "set shifting")	180208
#4	TITLE-ABS-KEY<br>(randomized OR randomised OR randomly OR trial)	4281552
#5	#1 AND #2 AND #3 AND #4	2303
CINAHL (EBSCO)		
#1	(MH "Child+") OR (MH "Adolescence+")	1214989
#2	TI child* OR AB child* OR<br>TI adolescen* OR AB adolescen* OR<br>TI youth* OR AB youth* OR<br>TI teen* OR AB teen* OR<br>TI pediatric* OR AB pediatric* OR<br>TI paediatric* OR AB paediatric*	912969
#3	#1 OR #2	605043
#4	(MH "Exercise+") OR<br>(MH "Exercise Therapy") OR<br>(MH "Physical Activity")	202631
#5	TI exercise* OR AB exercise* OR<br>TI training OR AB training OR<br>TI "physical activity" OR AB "physical activity" OR<br>TI "aerobic training" OR AB "aerobic training" OR<br>TI "resistance training" OR AB "resistance training" OR<br>TI "strength training" OR AB "strength training" OR<br>TI "interval training" OR AB "interval training" OR<br>TI sport* OR AB sport* OR<br>TI "physical education" OR AB "physical education"	514071
#6	#4 OR #5	122503
#7	(MH "Executive Function") OR<br>(MH "Working Memory")	9267
#8	TI "executive function*" OR AB "executive function*" OR<br>TI "working memory" OR AB "working memory" OR<br>TI "inhibitory control" OR AB "inhibitory control" OR<br>TI "response inhibition" OR AB "response inhibition" OR<br>TI "cognitive flexibility" OR AB "cognitive flexibility" OR<br>TI "cognitive control" OR AB "cognitive control"	27417
#9	#7 OR #8	6756
#10	(MH "Randomized Controlled Trials") OR<br>TI random* OR AB random* OR<br>TI trial OR AB trial	735071

#11	#3 AND #6 AND #9 AND #10	507
SPORTDiscus (EBSCO)		
#1	(DE "CHILDREN") OR (DE "YOUTH") OR (DE "TEENAGERS") OR (DE "PEDIATRICS")	71403
#2	TI child* OR AB child* OR<br>TI adolescen* OR AB adolescen* OR<br>TI youth* OR AB youth* OR<br>TI teen* OR AB teen* OR<br>TI pediatric* OR AB pediatric* OR<br>TI paediatric* OR AB paediatric*	156214
#3	#1 OR #2	41398
#4	(DE "EXERCISE") OR<br>(DE "EXERCISE therapy") OR<br>(DE "PHYSICAL activity") OR<br>(DE "STRENGTH training")	149462
#5	TI exercise* OR AB exercise* OR<br>TI training OR AB training OR<br>TI "physical activity" OR AB "physical activity" OR<br>TI "exercise training" OR AB "exercise training" OR<br>TI "physical training" OR AB "physical training" OR<br>TI "aerobic training" OR AB "aerobic training" OR<br>TI "resistance training" OR AB "resistance training" OR<br>TI "strength training" OR AB "strength training" OR<br>TI "interval training" OR AB "interval training" OR<br>TI sport* OR AB sport* OR<br>TI "physical education" OR AB "physical education"	697375
#6	#4 OR #5	120799
#7	(DE "EXECUTIVE functions") OR<br>(DE "WORKING memory")	855
#8	TI "executive function*" OR AB "executive function*" OR<br>TI "working memory" OR AB "working memory" OR<br>TI "inhibitory control" OR AB "inhibitory control" OR<br>TI "response inhibition" OR AB "response inhibition" OR<br>TI "cognitive flexibility" OR AB "cognitive flexibility" OR<br>TI "cognitive control" OR AB "cognitive control"	5080
#9	#7 OR #8	716
#10	TI random* OR AB random* OR<br>TI trial OR AB trial OR<br>TI "controlled trial" OR AB "controlled trial"	123682
#11	#3 AND #6 AND #9 AND #10	164
	ERIC	

	(child* OR adolescen* OR youth* OR teen* OR pediatric* OR paediatric* OR schoolchild* OR student* OR "school aged" OR "middle school" OR "secondary school" OR "high school") AND (exercise* OR "physical activity" OR "physical activities" OR "exercise training" OR "physical training" OR "aerobic exercise" OR "aerobic training" OR "resistance training" OR "strength training" OR "interval training" OR "high intensity interval training" OR HIIT OR "coordination training" OR "cognitively engaging exercise" OR sport* OR "physical education" OR running OR cycling OR swimming OR dance OR yoga) AND ("executive function" OR "executive functions" OR "executive functioning" OR "executive control" OR "cognitive control" OR "working memory" OR "inhibitory control" OR "response inhibition" OR "cognitive flexibility" OR "task switching" OR "set shifting" OR Stroop OR flanker OR "go/no-go" OR "go no go" OR "n-back" OR "n back")	230
	APA PsycINFO	
	(child* or adolescen* or youth* or teen* or pediatric* or paediatric* or schoolchild* or student* or "school aged" or "middle school" or "secondary school" or "high school").mp.	1985848
	(exercise* or "physical activity" or "physical activities" or "exercise training" or "physical training" or "aerobic exercise" or "aerobic training" or "resistance training" or "strength training" or "interval training" or "high intensity interval training" or HIIT or "coordination training" or "cognitively engaging exercise" or sport* or "physical education" or running or cycling or swimming or dance or yoga).mp.	236,090
	("executive function" or "executive functions" or "executive functioning" or "executive control" or "cognitive control" or "working memory" or "inhibitory control" or "response inhibition" or "cognitive flexibility" or "task switching" or "set shifting" or Stroop or flanker or "go/no-go" or "go no go" or "n-back" or "n back").mp.	130,990
	(random* or trial* or "controlled trial" or "randomized controlled trial" or "randomised controlled trial" or RCT).mp.	448071
	1 and 2 and 3 and 4	558
Total	PubMed + Web of Science + Embase + CENTRAL + Scopus + CINAHL + SPORTDiscus + ERIC + APA PsycINFO	8,522

2. Reasons for full-text exclusion

Following full-text eligibility assessment, 53 reports were excluded and 22 studies were included in the final review. For transparency, each excluded report was assigned a single primary reason for exclusion according to the predefined eligibility criteria. The most common reasons were an ineligible study design, including cross-over or non-randomised designs (n = 16), and an intervention duration of less than 4 weeks (n = 14). Other reasons included insufficient information for appropriate cluster adjustment (n = 8), an ineligible comparator (n = 7), insufficient or ineligible executive-function data (n = 4), participant age outside the predefined 10–19-year range (n = 3), and duplicate or secondary publication of an included trial (n = 1). The distribution of full-text exclusion reasons is summarised in Table S2.

Table S2. Summary of reasons for full-text exclusion

Alexandrino et al., 2026	Effect of 12 Sessions of Short-Duration High-Intensity Interval Training on Executive Function and Inhibitory Control in Healthy Adolescents: a Cluster Randomized Clinical Trial	Cluster RCT — insufficient cluster adjustment
Barene et al., 2025	Effects of short physical activity sessions on physical fitness and cognitive control in Norwegian upper secondary school students: the MOVE12 pilot study	No eligible post-intervention between-group EF outcome — EF was assessed only as an acute within-group response
Benzing et al., 2016	Acute Cognitively Engaging Exergame-Based Physical Activity Enhances Executive Functions in Adolescents	Intervention <4 weeks — single acute exercise bout
Birinci & Pancar, 2025	Comparison of Acute Irisin and Cognitive Responses to Different Exercise Modalities Among Late Adolescents	Cross-over design
Browne et al., 2016	Acute effect of vigorous aerobic exercise on the inhibitory control in adolescents	Cross-over design
Budde et al., 2010	Steroid hormones in the saliva of adolescents after different exercise intensities and their influence on working memory in a school setting	Intervention <4 weeks — single 12-min exercise bout
Che et al., 2026	The bidirectional modulation of acute aerobic exercise on cognitive performance and brain electrical activity in adolescents with smartphone addiction: an experimental study using EEG power spectrum analysis	Intervention <4 weeks
Cioffi & Lubetzky, 2023	BOXVR Versus Guided YouTube Boxing for Stress, Anxiety, and Cognitive Performance in Adolescents: a Pilot Randomized Controlled Trial	Intervention <4 weeks
Cooper et al., 2012	The effects of a mid-morning bout of exercise on adolescents' cognitive function	Cross-over design
Cooper et al., 2015	Breakfast glycaemic index and exercise: combined effects on adolescents' cognitive function and metabolism	Cross-over design
Cooper et al., 2016	Sprint-based exercise and cognitive function in adolescents	Cross-over design
Fernandes et al., 2022	Effects of Capoeira on children's executive functions: a randomized controlled trial	Age outside 10–19 years
Fu et al., 2021	Effects Of Acute Exercise On Cognitive Function In Different Exposures	Intervention <4 weeks
Hagins & Rundle, 2016	Yoga Improves Academic Performance in Urban High School Students Compared to Physical Education: A Randomized Controlled Trial	Cluster RCT — insufficient cluster adjustment
Harveson et al., 2016	Acute Effects of 30 Minutes Resistance and Aerobic Exercise on Cognition in a High School Sample	Cross-over design
Hawani et al., 2026	Effects of external verbal encouragement during minifootball on affective responses and post-training working memory in male adolescents	Cross-over design

Haweni et al., 2025	Optimizing learning and motor performance in highly trained youth football players: the role of time interval between synchronous and asynchronous sessions in a blended training model	Cross-over design
Hecht & Garber, 2021	Effectiveness of the POWER Program in Improving Physical Activity and Executive Function in Fifth Grade Students	Non-randomized/quasi-experimental design
ISRCTN11803379, 2023	Effects of exercise on brain and vascular functions in adolescents	Cross-over design
Jeon & Ha, 2017	The effect of exercise intensity on brain derived neurotrophic factor and memory in adolescents	Active-exercise comparator
Kang et al., 2020	Meditative Movement Affects Working Memory Related to Neural Activity in Adolescents: A Randomized Controlled Trial	Intervention <4 weeks
Kawabata et al., 2021	Breakfast and Exercise Improve Academic and Cognitive Performance in Adolescents	Intervention <4 weeks (single exercise bout)
Kjellenberg et al., 2024	Short, frequent physical activity breaks improve working memory while preserving cerebral blood flow in adolescents	Cross-over design
Lakes et al., 2013	The Healthy for Life Taekwondo pilot study: A preliminary evaluation of effects on executive function and BMI, feasibility, and acceptability	Active-exercise comparator
Leahy et al., 2020	Feasibility of test administration and preliminary findings for cognitive control in the Burn 2 Learn pilot randomised controlled trial	Cluster RCT — insufficient cluster adjustment
Ludyga et al., 2018	The effects of a school-based exercise program on neurophysiological indices of working memory operations in adolescents	Cluster RCT — insufficient cluster adjustment
Ludyga et al., 2021	Effects of Judo on Neurocognitive Indices of Response Inhibition in Preadolescent Children: a Randomized Controlled Trial	Age outside 10–19 years
Meha et al., 2025	A new injury prevention program 'FUNBALL' improves cognitive performance of young football (soccer) players: a cluster randomized controlled trial	Cluster RCT — insufficient cluster adjustment
Mero Piedra et al., 2023	Effects of a physical education intervention on attention and inhibitory control in Ecuadorian children with intellectual disabilities	Insufficient EF data
Mücke et al., 2020	The Influence of an Acute Exercise Bout on Adolescents' Stress Reactivity, Interference Control, and Brain Oxygenation Under Stress	Intervention <4 weeks
Mücke et al., 2023	Physical but not cognitive demand of an exercise bout influences subsequent affective inhibition	Cross-over design
Ringenbach et al., 2015	Assisted Cycling Therapy (ACT) improves inhibition in adolescents with autism spectrum disorder	Cross-over design
Ringenbach et al., 2016	The effects of assisted cycling therapy (ACT) and voluntary cycling on reaction time and measures of executive function	No randomized non-exercise control — inactive comparison

	in adolescents with Down syndrome	group was non-randomized
Robinson et al., 2022	Effects of Classroom-Based Resistance Training With and Without Cognitive Training on Adolescents' Cognitive Function, On-task Behavior, and Muscular Fitness	Cluster RCT — insufficient cluster adjustment
Roldan & Olmedo, 2025	Effect of low and high intentional velocity strength training on executive functions in children: a randomized controlled trial	Age outside 10–19 years
Ruiter et al., 2022	Effects of Desk-Bike Cycling on Phonological Working Memory Performance in Adolescents With Attention Deficit Hyperactivity Disorder	Cross-over design
Rutkauskaite et al., 2025	The Influence of Short-Term Dance-Oriented Exergaming on Cognitive Skills and Psychological Well-Being of Adolescents	Active-exercise comparator only
Shaw & Lubetzky, 2021	A Short Bout of Exercise With and Without an Immersive Virtual Reality Game Can Reduce Stress and Anxiety in Adolescents: A Pilot Randomized Controlled Trial	Intervention <4 weeks
Staiano et al., 2012	Competitive Versus Cooperative Exergame Play for African American Adolescents' Executive Function Skills: Short-Term Effects in a Long-Term Training Intervention	No eligible core EF outcome (composite D-KEFS only)
Stenman et al., 2017	Effects of Two-Week High-Intensity Interval Training on Cognition in Adolescents - A Randomized Controlled Pilot Study	Intervention <4 weeks
Subramanian et al., 2015	Effect of Structured and Unstructured Physical Activity Training on Cognitive Functions in Adolescents - A Randomized Control Trial	Active-exercise comparator
Tarp et al., 2016	Effectiveness of a School-Based Physical Activity Intervention on Cognitive Performance in Danish Adolescents: LCoMotion—Learning, Cognition and Motion—A Cluster Randomized Controlled Trial	Cluster RCT — insufficient cluster adjustment
Valkenborghs et al., 2022	Effect of high-intensity interval training on hippocampal metabolism in older adolescents	Duplicate/secondary report
van den Berg et al., 2018	Exercise of Varying Durations: no Acute Effects on Cognitive Performance in Adolescents	Cross-over design
Van Riper et al., 2023	Aerobic Exercise, Cognitive Performance, and Brain Activity in Adolescents with Attention-Deficit/Hyperactivity Disorder	Cross-over design
Wade et al., 2020	The impact of exercise environments on adolescents' cognitive and psychological outcomes: A randomised controlled trial	Intervention <4 weeks (single exercise bout)
Wang & Lipowski, 2026	Effects of high-intensity interval exercise on attentional network function in youth athletes	Intervention <4 weeks — acute HIIE session
Wang et al., 2025	An adaptive AI-based virtual reality sports system for adolescents with excess body weight: a randomized	Insufficient EF data

	controlled trial	
Wassenaar et al., 2021	The effect of a one-year vigorous physical activity intervention on fitness, cognitive performance and mental health in young adolescents: the Fit to Study cluster randomised controlled trial	Cluster RCT — insufficient cluster adjustment
Wen et al., 2021	Influence of acute exercise on inhibitory control and working memory of children: a comparison between soccer, resistance, and coordinative exercises	Intervention <4 weeks — acute exercise session
Williams et al., 2022	Effect of two-weeks of school-based sprint training on physical fitness, risk factors for cardiometabolic diseases and cognitive function in adolescent girls: A randomized controlled pilot trial	Intervention <4 weeks
Xie et al., 2025	Effects of community-based open and closed skill exercise interventions on ecological executive function and mobile phone dependence of adolescents	Active-exercise comparator
Zhang et al., 2026	Comparative effects of physical education interventions on motor ability and executive function in junior high school students and their interrelationships	Active-exercise comparator only

3. Characteristics of the included studies

The characteristics of the 22 included randomised controlled trials are summarised in Table S3. The included studies were published between 2015 and 2026 and were conducted across multiple countries and regions. Participants were adolescents aged 10–19 years and included healthy adolescents, adolescents with overweight or obesity, and adolescents with mental health concerns, including attention-deficit/hyperactivity disorder (ADHD), subthreshold depression, and elevated suicide risk. No restrictions were imposed on health status during study selection.

The exercise interventions varied substantially across studies and included aerobic exercise, high-intensity interval training, moderate-intensity continuous exercise, combined aerobic and resistance training, coordinative exercise, cognitively engaging movement games, open- and closed-skill exercise, basketball, badminton, rope skipping, taekwondo, swimming, yoga, small-sided games, football-based skill training, and classroom cycling. Control conditions included no intervention, wait-list control, usual daily activities, health education, and regular or standard physical education.

Executive function was assessed across the three prespecified core domains of inhibitory control, working memory, and cognitive flexibility. The included studies used a range of validated neuropsychological and computerised tasks,

including the Stroop task, Eriksen Flanker task, Go/No-Go task, N-back task, Sternberg working-memory task, Digit Span, Wisconsin Card Sorting Test, Trail Making Test, Posner task, task-switching paradigms, and the More-odd shifting task. Detailed information on study population, sample size, intervention and control conditions, executive-function assessments, and extracted outcomes is provided in Table S3.

Table S3. Characteristics of the included randomized controlled trials.

Author	Country	Year	Health status	Age (mean ± SD)	N	Intervention	Control	EF assessment	Outcome
Babayoğlu et al.	Türkiye	2026	Healthy	10–12 years (median 11.0)	T1: 26<br>T2: 26<br>C: 26	T1: Aerobic exercise<br>T2: Gross-motor coordination training	Standard physical education curriculum	Visual-Auditory Digit Span Task	Digit span total score (working memory)
Cai et al.	China	2025	Healthy	14.03 ± 0.17	T: 114<br>C: 108	HIIT-based classroom physical education exercise	Usual school physical education	N-back task (E-prime 1.0)	1-back RT<br>2-back RT
Chen et al.	Taiwan	2016	Obese	T: 12.64 ± 0.70<br>C: 12.84 ± 0.75	T: 25<br>C: 25	Moderate-intensity<br>physical activity program	Regular health education	WCST (CV4)	WCST nonperseverative errors<br>WCST total errors

Author	Country	Year	Health status	Age (mean ± SD)	N	Intervention	Control	EF assessment	Outcome
Cho et al.	Republic of Korea	2017	Healthy	T: 11.2 ± 0.8<br>C: 11.4 ± 0.7	T: 17<br>C: 18	Taekwondo training	No training	Stroop Color and Word Test	Stroop Color-Word (score)
Cho et al.	Republic of Korea	2017	Healthy	T: 11.20 ± 0.77<br>C: 11.33 ± 0.72	T: 15<br>C: 15	Taekwondo training	Usual school physical education	Children's Stroop Color and Word Test (Korean version)	Stroop Color-Word (score)
Chou et al.	Taiwan	2020	Overweight	T: 12.30 ± 0.66<br>C: 12.08 ± 0.69	T: 44<br>C: 40	Movement games	Original physical education lessons	Stroop test (Vienna Test System)	Stroop color-word interference RT (s)<br>Stroop incongruent color-naming RT (s)<br>Stroop incongruent word-reading RT (s)<br>Stroop word-color interference RT (s)

Author	Country	Year	Health status	Age (mean ± SD)	N	Intervention	Control	EF assessment	Outcome
Chou et al.	Taiwan	2022	Overweight	T1: 11.00 ± 0.64<br>T2: 11.37 ± 0.58<br>C: 11.32 ± 0.54	T1: 25<br>T2: 25<br>C: 25	T1: High-dose cognitively engaging movement games<br>T2: Low-dose cognitively engaging movement games	Sedentary activities	Computerized Stroop Test (Vienna Test System)	Stroop incongruent color-naming RT (s)<br>Stroop incongruent word-reading RT (s)<br>Stroop interference color-naming RT (s)<br>Stroop interference word-reading RT (s)
Dong et al.	China	2024	Healthy	T: 18.34 ± 0.58<br>C: 18.34 ± 0.48	T: 38<br>C: 32	Football juggling learning	Usual daily activities	Modified Flanker task<br>2-back task<br>Shifting task	Flanker incongruent ACC (%)<br>Flanker incongruent RT (ms)<br>2-back ACC (%)<br>2-back RT (ms)<br>Shifting heterogeneous ACC (%)<br>Shifting heterogeneous RT (ms)

Author	Country	Year	Health status	Age (mean ± SD)	N	Intervention	Control	EF assessment	Outcome
Gejl et al.	Denmark	2024	Healthy	T1: 17.9 ± 0.8<br>T2: 17.8 ± 0.8<br>C: 17.9 ± 0.9	T1: 23<br>T2: 25<br>C: 34	T1: Moderate-intensity aerobic training<br>T2: High-intensity aerobic training (cycling and running)	Passive control; habitual lifestyle	Modified Eriksen Flanker task	Flanker interference RT (ms)<br>Flanker interference accuracy (%-point)
Gu et al.	China	2024	Subthreshold depression	T1: 16.50 ± 0.31<br>T2: 16.65 ± 0.44<br>C: 17.17 ± 0.54	T1: 33<br>T2: 29<br>C: 33	T1: Open-skill exercise (badminton)<br>T2: Closed-skill exercise (running)	Regular physical education	Eriksen Flanker task<br>Task-switching test<br>N-back task	Task-switching switch IES (ms)<br>Flanker incongruent IES (ms)<br>1-back IES (ms)<br>2-back IES (ms)<br>3-back IES (ms)
Huang et al.	China	2026	Healthy	NR	T: 32<br>C: 32	Moderate-intensity basketball intervention	Regular physical education only	Flanker task<br>2-back task<br>More-odd shifting task (E-Prime 3.0)	Flanker conflict-effect RT (ms)<br>2-back mean RT (ms)<br>More-odd switch-cost RT (ms)

Author	Country	Year	Health status	Age (mean ± SD)	N	Intervention	Control	EF assessment	Outcome
Jeong et al.	South Korea	2021	High suicide risk	T: 15.31 ± 0.63<br>C: 15.46 ± 0.52	T: 13<br>C: 13	Aerobic exercise (badminton and rope jumping)	No exercise	Sternberg Digit Working Memory Task	Sternberg easy ACC (%)<br>Sternberg easy RT (ms)<br>Sternberg hard ACC (%)<br>Sternberg hard RT (ms)
Kadri et al.	Tunisia	2019	ADHD	T: 14.5 ± 3.5<br>C: 14.2 ± 3.0	T: 20<br>C: 20	Taekwondo practice	Usual school physical education	Stroop Color-Word Test	Color-Word interference score<br>Interference score
Liu et al.	Taiwan	2018	Obese	T: 13.94 ± 0.94<br>C: 14.17 ± 0.71	T: 35<br>C: 35	Coordination exercise (jump rope)	Waitlist control	Stroop task (including food-cue Stroop)	Stroop congruent RT (s)<br>Stroop color RT (s)<br>Stroop incongruent RT (s)<br>Food-cue Stroop RT (s)
Liu et al.	Hong Kong, China	2025	ADHD	T: 15.82 ± 1.11<br>C: 13.65 ± 1.21	T: 40<br>C: 40	Aerobic exercise-based physical activity	Usual daily activities	Posner test	Valid-trial RT (ms)<br>Invalid-trial RT (ms)<br>Neutral-trial RT (ms)

Author	Country	Year	Health status	Age (mean ± SD)	N	Intervention	Control	EF assessment	Outcome
Liu et al.	China	2026	Healthy	T: 13.4 ± 0.4<br>C: 13.5 ± 0.5	T: 98<br>C: 105	Combined aerobic and resistance training (HIIT + resistance exercise)	Regular physical education + reading/homework activities	Eriksen Flanker task<br>N-back task<br>More-odd shifting task (E-Prime 1.1)	Inhibitory-control RT (ms)<br>2-back RT (ms)<br>Cognitive-flexibility RT (ms)
Martín-Martínez et al.	Spain	2015	Healthy	15.35 ± 0.48	Total: 54	Small-sided games	Usual school physical education	WISC-IV Digit and Letter-Number tests<br>Trail Making Test<br>Stroop Test	Digit score<br>Letter-Number score<br>Working Memory Index<br>TMT-B time<br>Stroop Color-Word score
Purohit et al.	India	2017	Healthy	T: 12.69 ± 1.35<br>C: 12.58 ± 1.52	T: 40<br>C: 32	Yoga	Waitlist control	Stroop Color-Word Task<br>Digit Span Test<br>Trail Making Test	Stroop Color-Word score<br>Digit Span score<br>TMT-B time

Author	Country	Year	Health status	Age (mean ± SD)	N	Intervention	Control	EF assessment	Outcome
Roh et al.	Republic of Korea	2018	Healthy	T: 11.53 ± 0.64<br>C: 11.40 ± 0.63	T: 15<br>C: 15	Taekwondo training	Usual school physical education	Children's Stroop Color and Word Test (Korean version)	Stroop Color-Word (score)
Silva et al.	Brazil	2019	ADHD	T: 12.2 ± 2.0<br>C: 12.0 ± 1.0	T: 10<br>C: 10	Swimming-learning program	No training	Trail Making Test (parts A and B)	TMT cognitive flexibility score
Torbeyns et al.	Belgium	2017	Healthy	14.3 ± 0.6	T: 21<br>C: 23	Bike desk cycling	No intervention	Stroop test (E-prime 2.0)	Color-incongruent ACC (%)<br>Word-incongruent ACC (%)<br>Color-incongruent RT (ms)<br>Word-incongruent RT (ms)

Author	Country	Year	Health status	Age (mean ± SD)	N	Intervention	Control	EF assessment	Outcome
Wang et al.	China	2026	Obese, low SES	T1: 13.48 ± 1.12<br>T2: 13.80 ± 1.87<br>C: 13.44 ± 1.59	T1: 25<br>T2: 25<br>C: 25	T1: High-intensity interval exercise<br>T2: Moderate-intensity continuous exercise	Usual daily and academic activities	GO/NOGO task<br>2-back task<br>More-odd shifting task (E-prime 2.0)	GO/NOGO RT (ms)<br>GO/NOGO ACC (%)<br>2-back RT (ms)<br>2-back ACC (%)<br>More-odd shifting RT (ms)<br>More-odd shifting ACC (%)

Note. T, intervention group; C, control group; EF, executive function; RT, reaction time; ACC, accuracy; IES, inverse efficiency score; HIIT, high-intensity interval training; NR, not reported.

4. Main analyses of executive function outcomes

The primary analysis included 22 independent RCTs, 27 eligible exercise–control comparisons, and 86 effect sizes. Using a three-level random-effects model with study-clustered CR2 robust confidence intervals, exercise interventions were associated with a statistically significant beneficial effect across the pooled executive-function outcomes (Hedges' $g = 0.55$, 95% CI 0.33 to 0.77, $p < 0.001$). Positive effect sizes indicated better executive-function performance in the exercise group. Because the overall analysis combined effect sizes across different executive-function domains and tasks, this pooled estimate represents the average effect across the available executive-function outcomes rather than an equally weighted improvement in each domain. Domain-specific analyses were therefore conducted separately.

For inhibitory control, 17 RCTs, 21 comparisons, and 47 effect sizes were included. Exercise produced a significant beneficial effect (Hedges' $g = 0.56$, 95% CI 0.28 to 0.84, $p < 0.001$). For working memory, 10 RCTs, 13 comparisons, and 25 effect sizes contributed to the analysis, with a significant pooled effect favouring exercise (Hedges' $g = 0.51$, 95% CI 0.18 to 0.84, $p = 0.007$). Similarly, the analysis of cognitive flexibility, based on 9 RCTs, 11 comparisons, and 14 effect sizes, showed a significant beneficial effect of exercise (Hedges' $g = 0.43$, 95% CI 0.30 to 0.57, $p < 0.001$).

Taken together, the updated analyses indicated statistically significant beneficial associations of chronic exercise with all three core executive-function domains. The largest pooled estimate was observed for inhibitory control, followed by working memory and cognitive flexibility, although the magnitude of these estimates should be interpreted together with their confidence intervals and between-study heterogeneity rather than as evidence of statistically significant differences between domains.

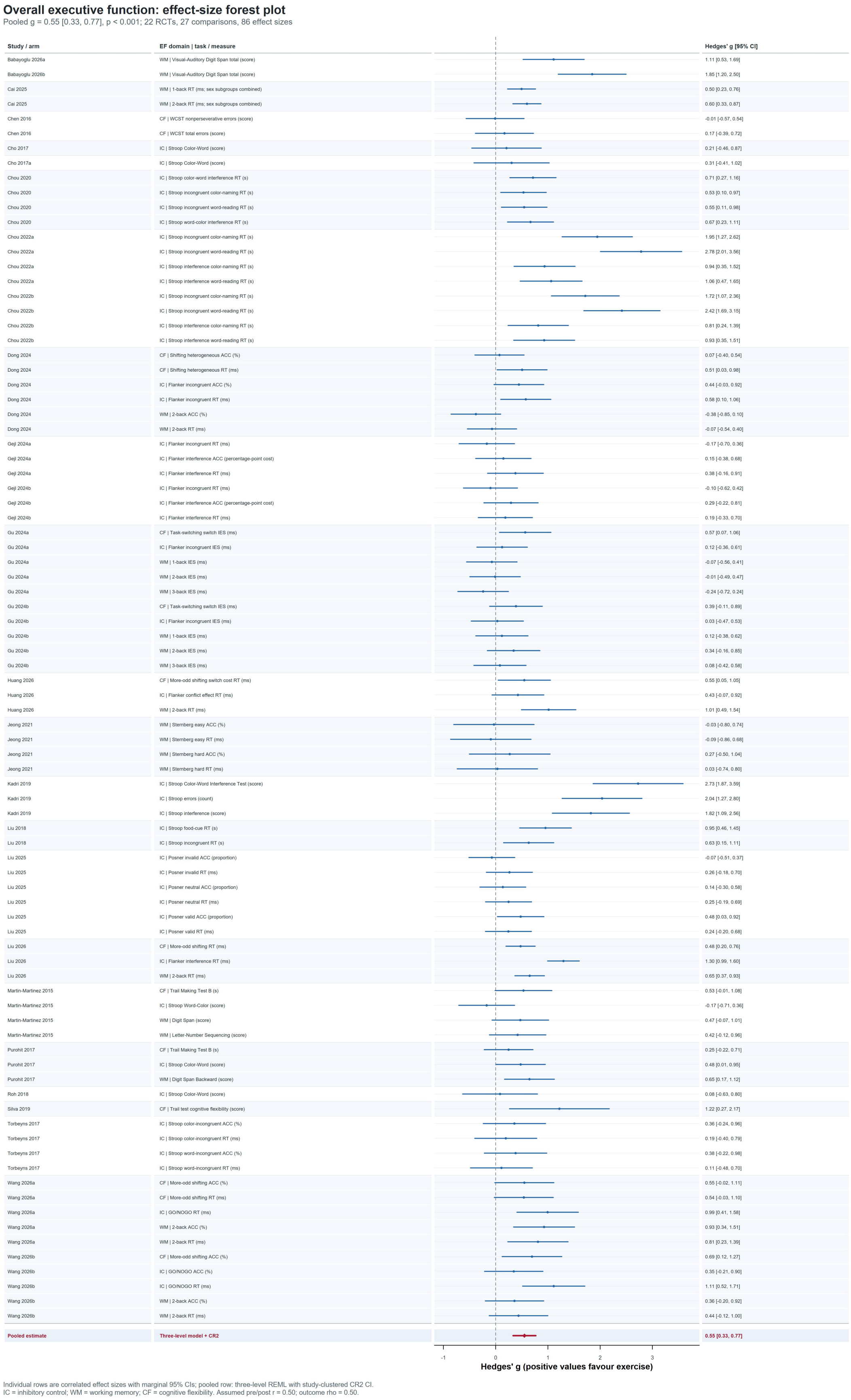

Figure S1. Overall executive function: effect-size forest plot.

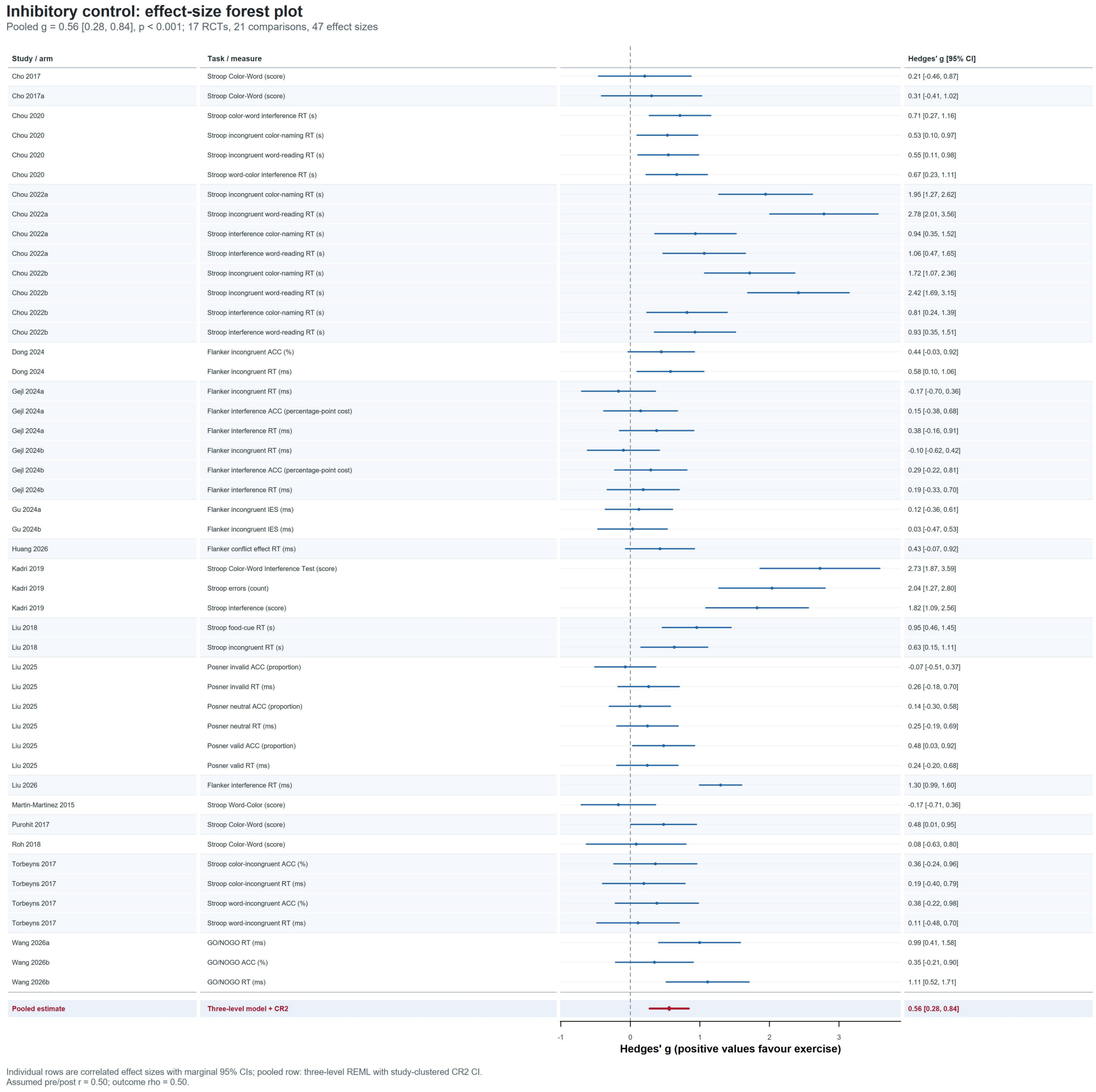

Figure S2. Inhibitory control: effect-size forest plot.

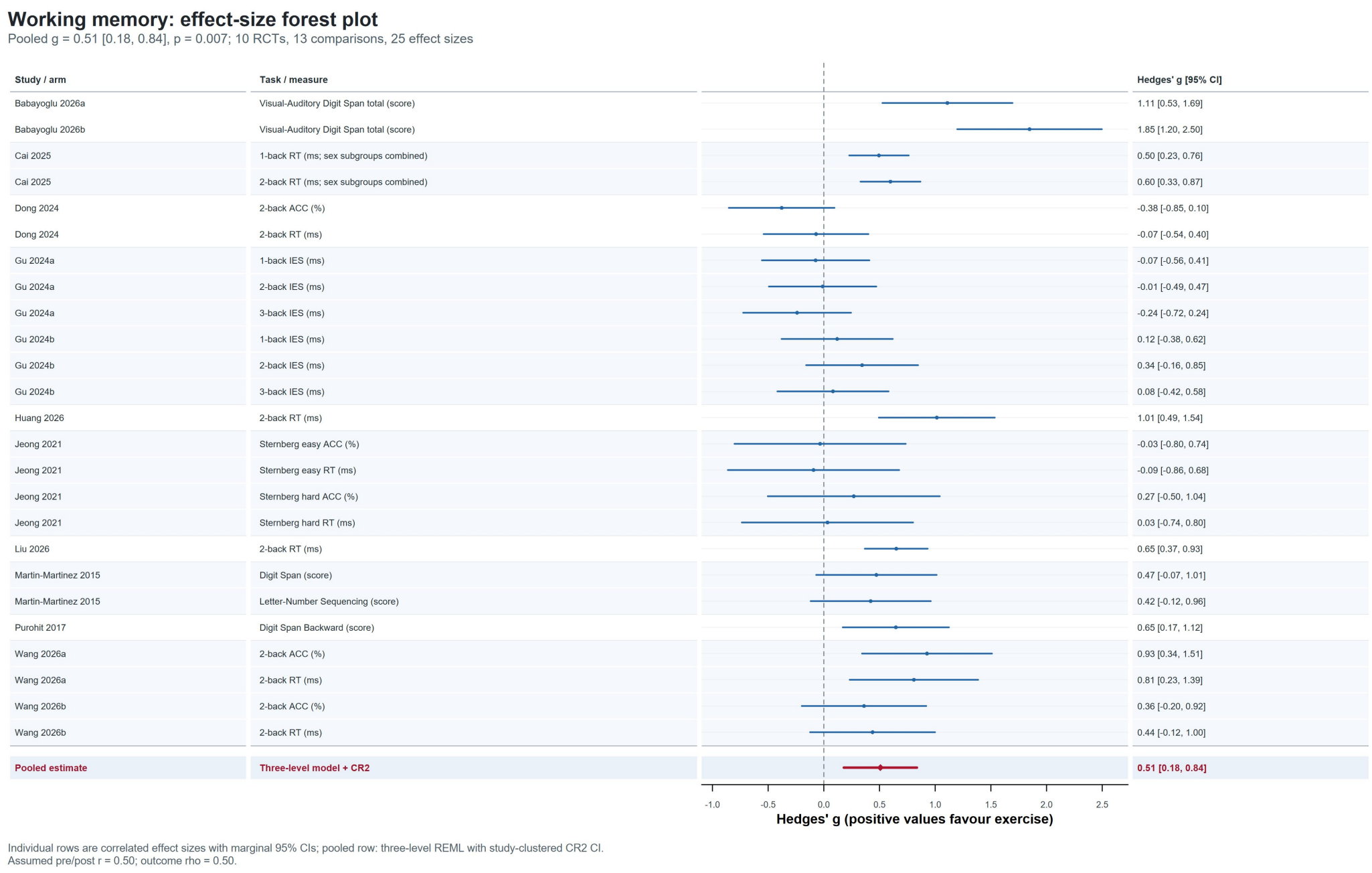

Figure S3. Working memory: effect-size forest plot.

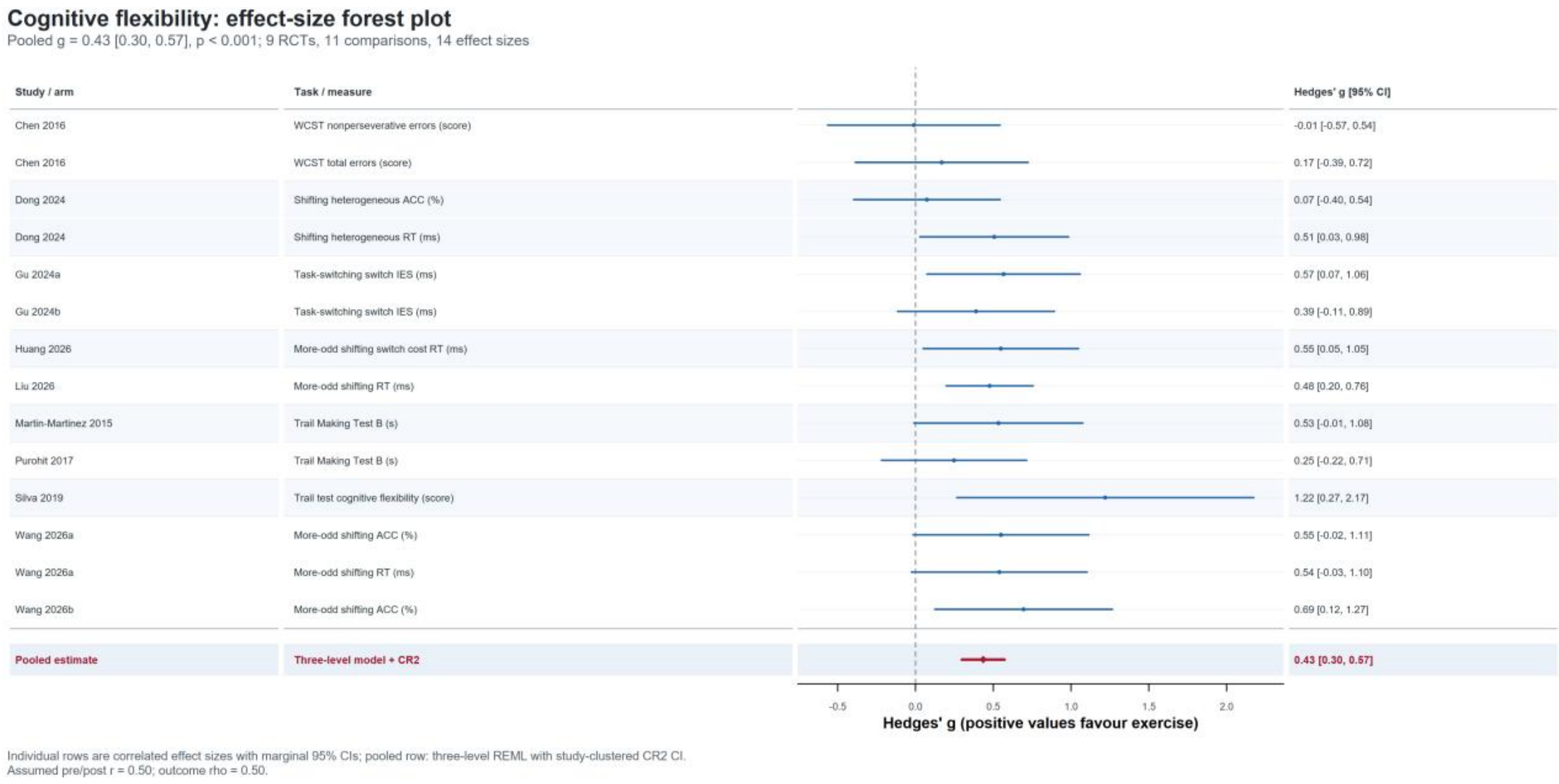

Figure S4. Cognitive flexibility: effect-size forest plot.

5. Exploratory subgroup analysis by population health status

Exploratory subgroup analyses were conducted to examine whether the effect of exercise on overall executive function differed across more clearly defined adolescent populations. To address clinical heterogeneity in the previous classification of mental health conditions, subgroup analyses were restricted to three interpretable population categories: healthy adolescents, adolescents with overweight/obesity, and adolescents with ADHD. Trials involving adolescents with subthreshold depression or elevated suicide risk were retained in the overall meta-analysis but were not combined with ADHD in the subgroup comparison because these populations differ substantially in clinical characteristics, baseline executive-function profiles, and intervention goals. Accordingly, the subgroup comparison included 20 RCTs, whereas the overall pooled estimate was based on all 22 RCTs.

Across all 22 RCTs, exercise interventions were associated with a significant beneficial effect on overall executive function (Hedges' g = 0.55, 95% CI 0.33 to 0.77, $p < 0.001$). Among healthy adolescents, 12 RCTs contributed 14 comparisons and 36 effect sizes, yielding a significant pooled effect (g = 0.46, 95% CI 0.23 to 0.69, p = 0.001). Among adolescents with overweight/obesity, 5 RCTs contributed 7 comparisons and 26 effect sizes, and exercise was also associated with a significant beneficial effect (g = 0.72, 95% CI 0.16 to 1.27, p = 0.023). For adolescents with ADHD, 3 RCTs contributed 3 comparisons and 10 effect sizes; the pooled estimate was larger in magnitude but imprecise and not statistically significant (g = 1.14, 95% CI −1.41 to 3.70, p = 0.192).

The omnibus test did not indicate a statistically significant difference in effect size across the three population subgroups (CR2/HTZ p = 0.530). Therefore, although the point estimates differed numerically, there was insufficient evidence to conclude that the effect of exercise on executive function varied by population health status. Given

the small number of studies in some subgroups, particularly the ADHD subgroup, these analyses should be regarded as exploratory.

Table S4. Exploratory population-stratified three-level meta-analysis of overall executive function.

Population	RCTs, n	Comparisons, n	Effect sizes, n	Hedges' g	95% CI (CR2)	p-value (CR2)	tau2 Level 2	tau2 Level 3	I2 Level 2 (%)	I2 Level 3 (%)	Total I2 (%)
Overall population	22	27	86	0.55	0.33 to 0.77	<0.001	0.05	0.162	20.03	64.89	84.92
Healthy	12	14	36	0.46	0.23 to 0.69	0.001	0.064	0.051	42.83	34.07	76.9
Overweight/Obesity	5	7	26	0.72	0.16 to 1.27	0.023	0.082	0.125	32.93	50.48	83.41
ADHD	3	3	10	1.14	-1.41 to 3.70	0.192	0.013	0.87	1.42	94.36	95.78

Note. Subgroup analyses were restricted to healthy adolescents, adolescents with overweight/obesity, and adolescents with ADHD. CR2, trial-clustered robust inference; Level 2, residual heterogeneity among effect sizes within trials; Level 3, between-trial heterogeneity. The omnibus subgroup test was not statistically significant (CR2/HTZ p = 0.530).

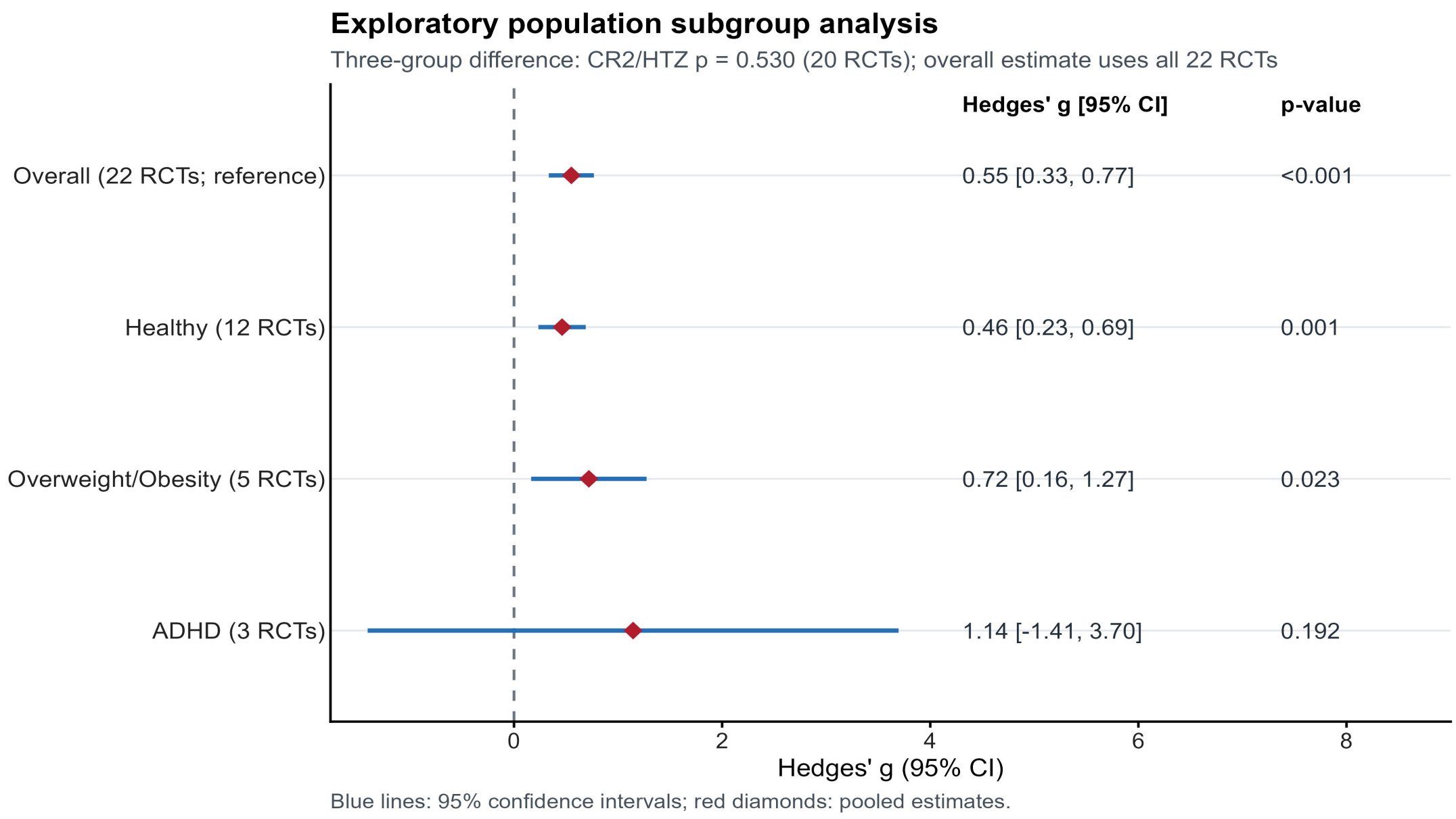

Figure S5. Exploratory subgroup analysis of overall executive function by population health status.

6. Sensitivity analyses

Four complementary sensitivity analyses were conducted to evaluate the robustness of the primary three-level meta-analytic findings: leave-one-RCT-out analyses, exclusion of high-risk-of-bias results, reviewer-focused task-classification analyses, and analyses under alternative within-study outcome and pre–post correlation assumptions. Robustness was judged from the stability of pooled Hedges' g estimates, 95% confidence intervals, statistical significance, and the Level-2 and Level-3 heterogeneity components.

6.1 Leave-one-RCT-out sensitivity analyses

To determine whether any single trial disproportionately influenced the pooled estimates, each independent RCT was removed in turn and the corresponding three-level model was refitted. The pooled effects remained statistically significant in every iteration. The leave-one-out Hedges' g estimates ranged from 0.49 to 0.58 for overall executive function, 0.48 to 0.60 for inhibitory control, 0.42 to 0.59 for working memory, and 0.41 to 0.47 for cognitive flexibility. Detailed estimates are presented in Tables S5–S8 and Figures S6–S9.

Table S5. Leave-one-RCT-out sensitivity analysis for overall executive function.

Excluded study	RCTs / comparisons / effects	Hedges' g (95% CI)	p-value	τ^2 Level 2	τ^2 Level 3	I^2 Level 2 (%)	I^2 Level 3 (%)	Δg vs full
**Full dataset**	**22 / 27 / 86**	**0.55 (0.33–0.77)**	**<0.001**	**0.050**	**0.162**	**20.03**	**64.89**	**0.000**
Babayoglu_2026	21 / 25 / 84	0.51 (0.30–0.72)	<0.001	0.047	0.134	21.65	61.33	-0.040
Cai_2025	21 / 26 / 84	0.55 (0.32–0.78)	<0.001	0.052	0.179	19.42	66.05	0.002
Chen_2016	21 / 26 / 84	0.57 (0.35–0.80)	<0.001	0.051	0.164	20.33	64.88	0.022

Cho_2017	21 / 26 / 85	0.56 (0.34–0.79)	<0.001	0.050	0.169	19.61	65.83	0.012
Cho_2017a	21 / 26 / 85	0.56 (0.34–0.78)	<0.001	0.050	0.171	19.52	66.00	0.009
Chou_2020	21 / 26 / 82	0.55 (0.32–0.78)	<0.001	0.055	0.178	20.38	65.55	-0.002
Chou_2022	21 / 25 / 78	0.51 (0.30–0.71)	<0.001	0.037	0.134	17.99	64.31	-0.043
Dong_2024	21 / 26 / 80	0.57 (0.35–0.79)	<0.001	0.043	0.168	17.19	67.53	0.019
Gejl_2024	21 / 25 / 80	0.58 (0.35–0.80)	<0.001	0.054	0.164	21.27	64.03	0.025
Gu_2024	21 / 25 / 76	0.58 (0.35–0.80)	<0.001	0.057	0.163	21.86	62.98	0.026
Huang_2026	21 / 26 / 83	0.55 (0.32–0.78)	<0.001	0.050	0.176	18.92	66.84	-0.004
Jeong_2021	21 / 26 / 82	0.57 (0.35–0.80)	<0.001	0.053	0.164	20.88	64.63	0.022
Kadri_2019	21 / 26 / 83	0.49 (0.31–0.67)	<0.001	0.048	0.083	28.62	49.44	-0.063

Liu_2018	21 / 26 / 84	0.54 (0.31–0.77)	<0.001	0.051	0.172	19.50	66.10	-0.011
Liu_2025	21 / 26 / 80	0.57 (0.34–0.80)	<0.001	0.056	0.170	21.14	64.25	0.021
Liu_2026	21 / 26 / 83	0.54 (0.31–0.76)	<0.001	0.040	0.173	15.81	68.35	-0.016
Martin-Martinez _2015	21 / 26 / 82	0.56 (0.34–0.79)	<0.001	0.049	0.174	18.92	66.72	0.013
Purohit_2017	21 / 26 / 83	0.56 (0.33–0.79)	<0.001	0.052	0.177	19.51	66.34	0.006
Roh_2018	21 / 26 / 85	0.57 (0.34–0.79)	<0.001	0.050	0.167	19.79	65.49	0.015
Silva_2019	21 / 26 / 85	0.53 (0.32–0.75)	<0.001	0.050	0.162	20.07	64.95	-0.016
Torbeyns_2017	21 / 26 / 82	0.57 (0.34–0.79)	<0.001	0.054	0.172	20.50	65.36	0.015
Wang_2026	21 / 25 / 76	0.55 (0.32–0.78)	<0.001	0.054	0.177	19.98	66.09	-0.004

Note. Each independent RCT was removed once and the three-level random-effects model was refitted. The full dataset is shown as the reference row. Δg denotes the change in pooled Hedges' g relative to the full dataset. Level 2 represents residual heterogeneity among effect sizes within trials and Level 3 represents between-trial heterogeneity. Positive Hedges' g values favor exercise.

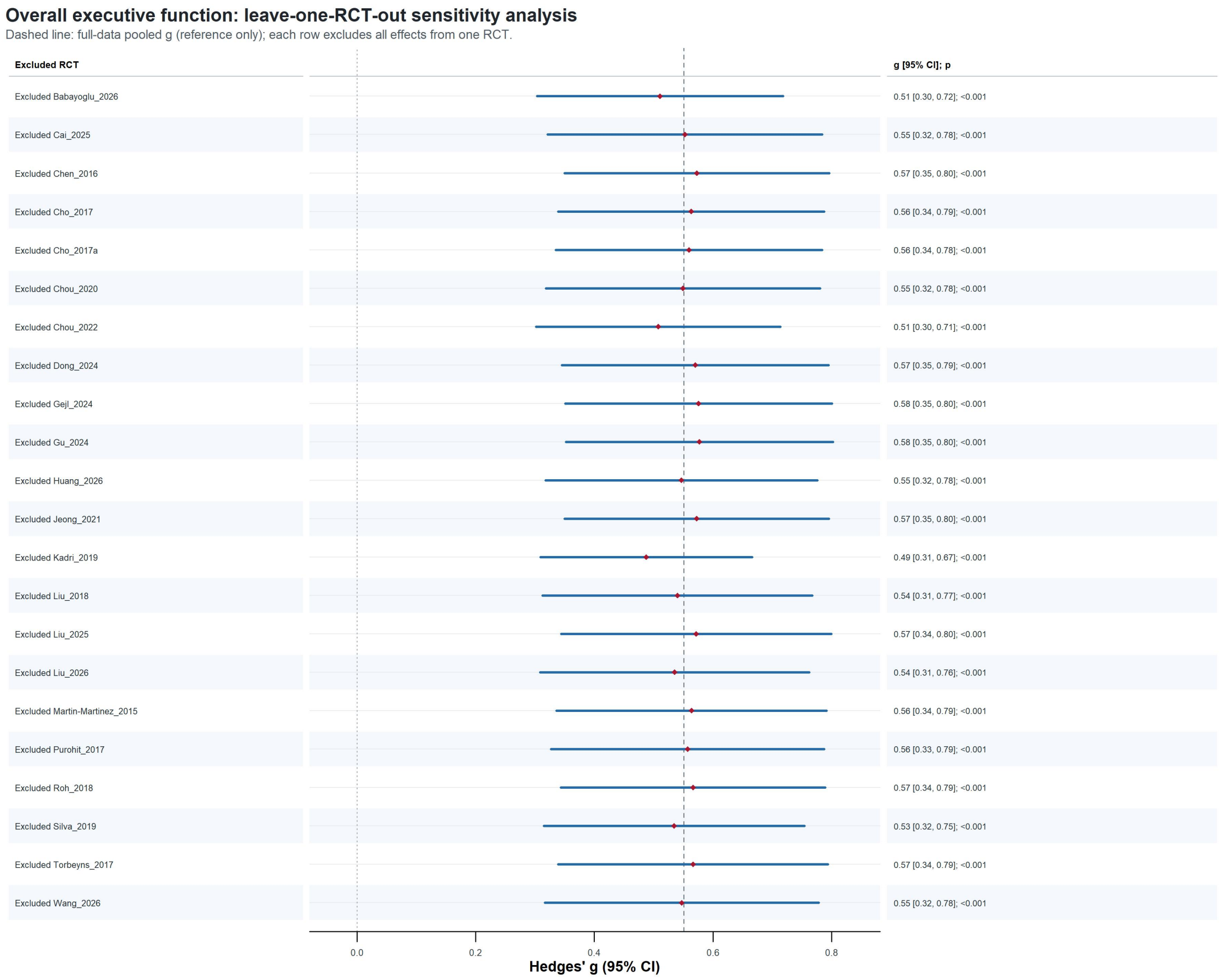

Figure S6. Leave-one-RCT-out sensitivity analysis for overall executive function.

Table S6. Leave-one-RCT-out sensitivity analysis for inhibitory control.

Excluded study	RCTs / comparisons / effects	Hedges' g (95% CI)	p-value	τ^2 Level 2	τ^2 Level 3	I^2 Level 2 (%)	I^2 Level 3 (%)
**Full dataset**	**17 / 21 / 47**	**0.56 (0.28–0.84)**	**<0.001**	**0.034**	**0.219**	**11.55**	**73.38**
Cho_2017	16 / 20 / 46	0.58 (0.28–0.87)	<0.001	0.035	0.230	11.40	74.28
Cho_2017a	16 / 20 / 46	0.57 (0.27–0.87)	<0.001	0.035	0.233	11.33	74.47

Chou_2020	16 / 20 / 43	0.56 (0.25–0.87)	0.002	0.053	0.243	15.53	70.77
Chou_2022	16 / 19 / 39	0.50 (0.23–0.78)	0.001	0.001	0.206	0.47	81.90
Dong_2024	16 / 20 / 45	0.56 (0.26–0.87)	0.001	0.040	0.241	12.38	73.84
Gejl_2024	16 / 19 / 41	0.60 (0.30–0.90)	<0.001	0.050	0.222	15.69	69.86
Gu_2024	16 / 19 / 45	0.59 (0.30–0.89)	<0.001	0.040	0.221	13.20	72.17
Huang_2026	16 / 20 / 46	0.57 (0.27–0.87)	0.001	0.036	0.238	11.19	74.76
Kadri_2019	16 / 20 / 44	0.48 (0.25–0.71)	<0.001	0.030	0.116	15.74	61.37
Liu_2018	16 / 20 / 45	0.54 (0.24–0.85)	0.002	0.036	0.237	11.36	74.52
Liu_2025	16 / 20 / 41	0.59 (0.29–0.90)	<0.001	0.056	0.230	16.64	68.73
Liu_2026	16 / 20 / 46	0.51 (0.23–0.79)	0.002	0.032	0.193	11.77	71.33

Martin-Martinez_2015	16 / 20 / 46	0.60 (0.32–0.88)	<0.001	0.034	0.205	12.00	72.29
Purohit_2017	16 / 20 / 46	0.56 (0.26–0.87)	0.001	0.036	0.239	11.15	74.83
Roh_2018	16 / 20 / 46	0.58 (0.29–0.87)	<0.001	0.035	0.226	11.52	73.94
Torbeyns_2017	16 / 20 / 43	0.58 (0.28–0.89)	<0.001	0.048	0.233	14.68	71.74
Wang_2026	16 / 19 / 44	0.54 (0.24–0.84)	0.002	0.025	0.238	8.12	77.49

Note. Each independent RCT contributing to inhibitory control was removed once and the three-level random-effects model was refitted. The full dataset is shown as the reference row. Level 2 represents residual heterogeneity among effect sizes within trials and Level 3 represents between-trial heterogeneity. Positive Hedges' g values favor exercise.

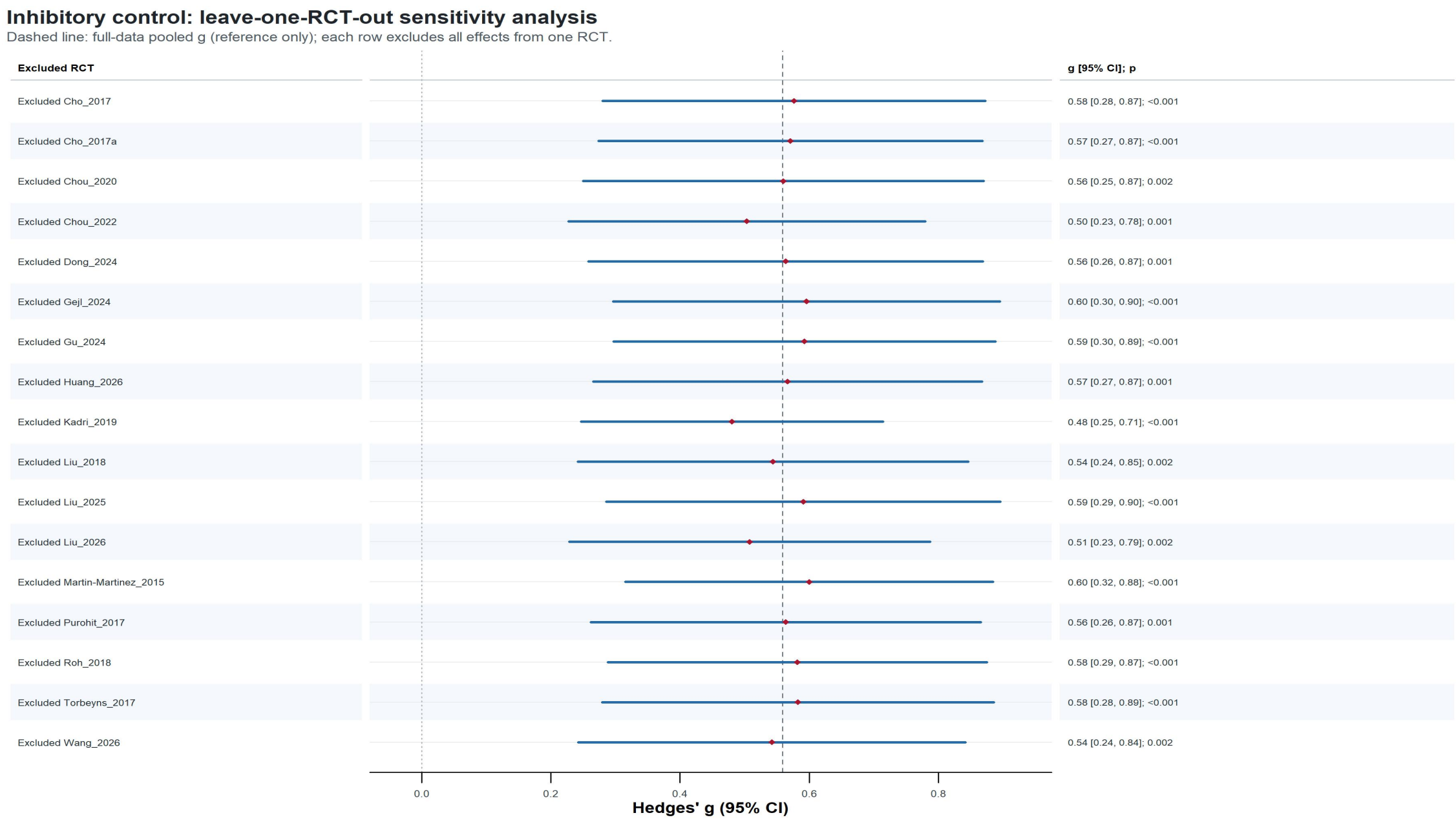

Figure S7. Leave-one-RCT-out sensitivity analysis for inhibitory control.

Table S7. Leave-one-RCT-out sensitivity analysis for working memory.

Excluded study	RCTs / comparisons / effects	Hedges' g (95% CI)	p-value	τ² Level 2	τ² Level 3	I² Level 2 (%)	I² Level 3 (%)
**Full dataset**	**10 / 13 / 25**	**0.51 (0.18–0.84)**	**0.007**	**<0.001**	**0.162**	**<0.001**	**80.67**
Babayoglu_2026	9 / 11 / 23	0.42 (0.13–0.71)	0.010	<0.001	0.096	<0.001	72.07
Cai_2025	9 / 12 / 23	0.50 (0.12–0.89)	0.016	<0.001	0.195	<0.001	81.01
Dong_2024	9 / 12 / 23	0.59 (0.29–0.89)	0.002	<0.001	0.106	<0.001	73.39
Gu_2024	9 / 11 / 19	0.57 (0.22–0.91)	0.005	<0.001	0.151	<0.001	78.33
Huang_2026	9 / 12 / 24	0.46 (0.11–0.80)	0.016	<0.001	0.156	<0.001	80.35
Jeong_2021	9 / 12 / 21	0.55 (0.20–0.90)	0.007	<0.001	0.166	<0.001	82.70
Liu_2026	9 / 12 / 24	0.49 (0.11–0.87)	0.017	<0.001	0.190	<0.001	82.65

Martin-Martinez_2015	9 / 12 / 23	0.52 (0.14–0.89)	0.013	<0.001	0.189	<0.001	83.38
Purohit_2017	9 / 12 / 24	0.49 (0.12–0.87)	0.016	<0.001	0.186	<0.001	83.00
Wang_2026	9 / 11 / 21	0.50 (0.12–0.87)	0.016	<0.001	0.189	<0.001	83.44

Note. Each independent RCT contributing to working memory was removed once and the three-level random-effects model was refitted. The full dataset is shown as the reference row. Level 2 represents residual heterogeneity among effect sizes within trials and Level 3 represents between-trial heterogeneity. Positive Hedges' g values favor exercise.

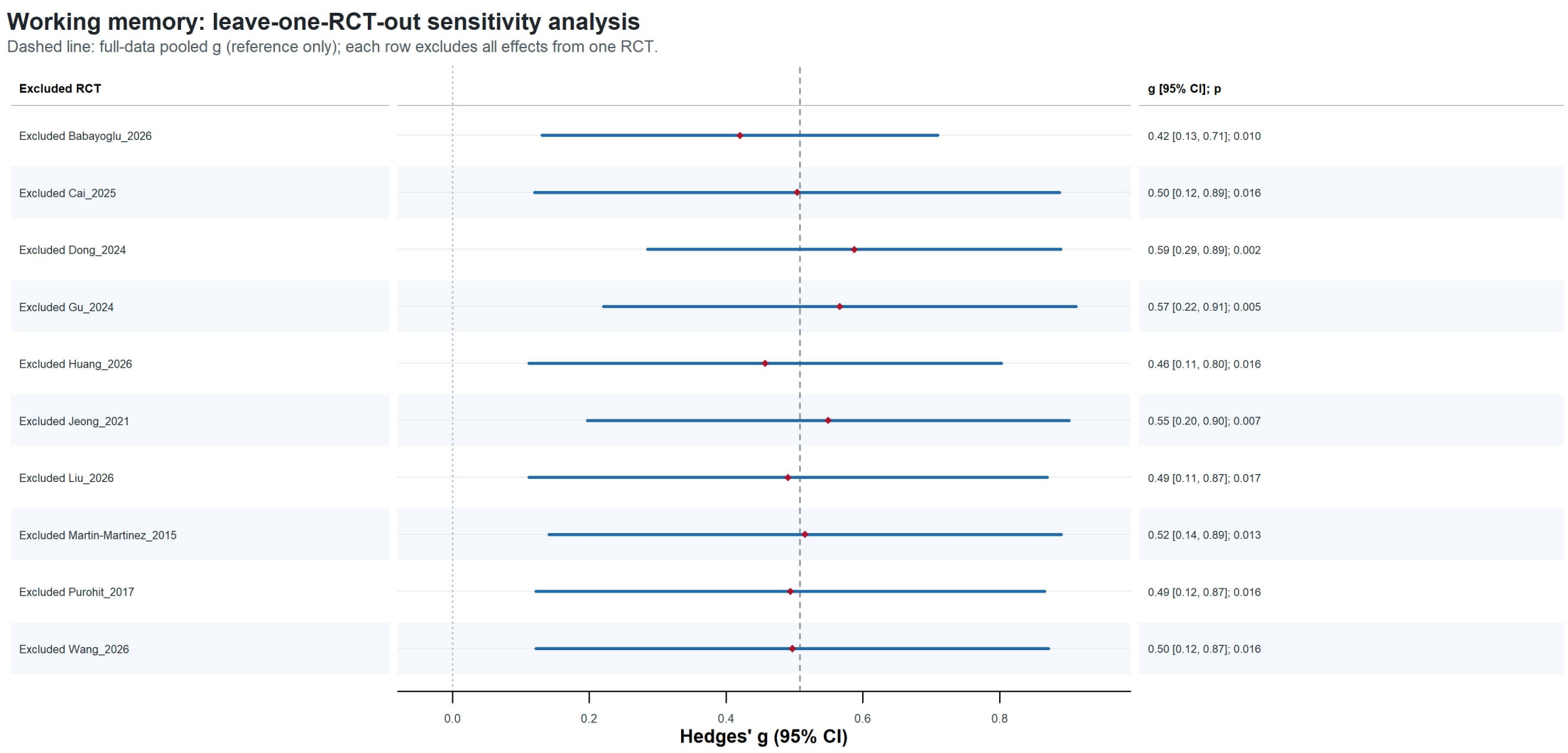

Figure S8. Leave-one-RCT-out sensitivity analysis for working memory.

Table S8. Leave-one-RCT-out sensitivity analysis for cognitive flexibility.

Excluded study	RCTs / comparisons / effects	Hedges' g (95% CI)	p-value	τ² Level 2	τ² Level 3	I² Level 2 (%)	I² Level 3 (%)
**Full dataset**	**9 / 11 / 14**	**0.43 (0.30–0.57)**	**<0.001**	**<0.01**	**<0.01**	**<0.01**	**<0.01**
Chen_2016	8 / 10 / 12	0.47	<0.001	<0.01	<0.01	<0.01	<0.01

		(0.35–0.59)					
Dong_2024	8 / 10 / 12	0.46 (0.31–0.61)	<0.001	<0.01	<0.01	<0.01	<0.01
Gu_2024	8 / 9 / 12	0.43 (0.27–0.59)	0.001	<0.01	<0.01	<0.01	<0.01
Huang_2026	8 / 10 / 13	0.42 (0.27–0.58)	<0.001	<0.01	<0.01	<0.01	<0.01
Liu_2026	8 / 10 / 13	0.42 (0.24–0.60)	0.001	<0.01	<0.01	<0.01	<0.01
Martin-Martinez_2015	8 / 10 / 13	0.43 (0.27–0.58)	<0.001	<0.01	<0.01	<0.01	<0.01
Purohit_2017	8 / 10 / 13	0.46 (0.31–0.60)	<0.001	<0.01	<0.01	<0.01	<0.01
Silva_2019	8 / 10 / 13	0.42 (0.28–0.55)	<0.001	<0.01	<0.01	<0.01	<0.01
Wang_2026	8 / 9 / 11	0.41 (0.26–0.57)	<0.001	<0.01	<0.01	<0.01	<0.01

Note. Each independent RCT contributing to cognitive flexibility was removed once and the three-level random-effects model was refitted. The full dataset is shown as the reference row. Level 2 represents residual heterogeneity among effect sizes within trials and Level 3 represents between-trial heterogeneity. Positive Hedges' g values favor exercise.

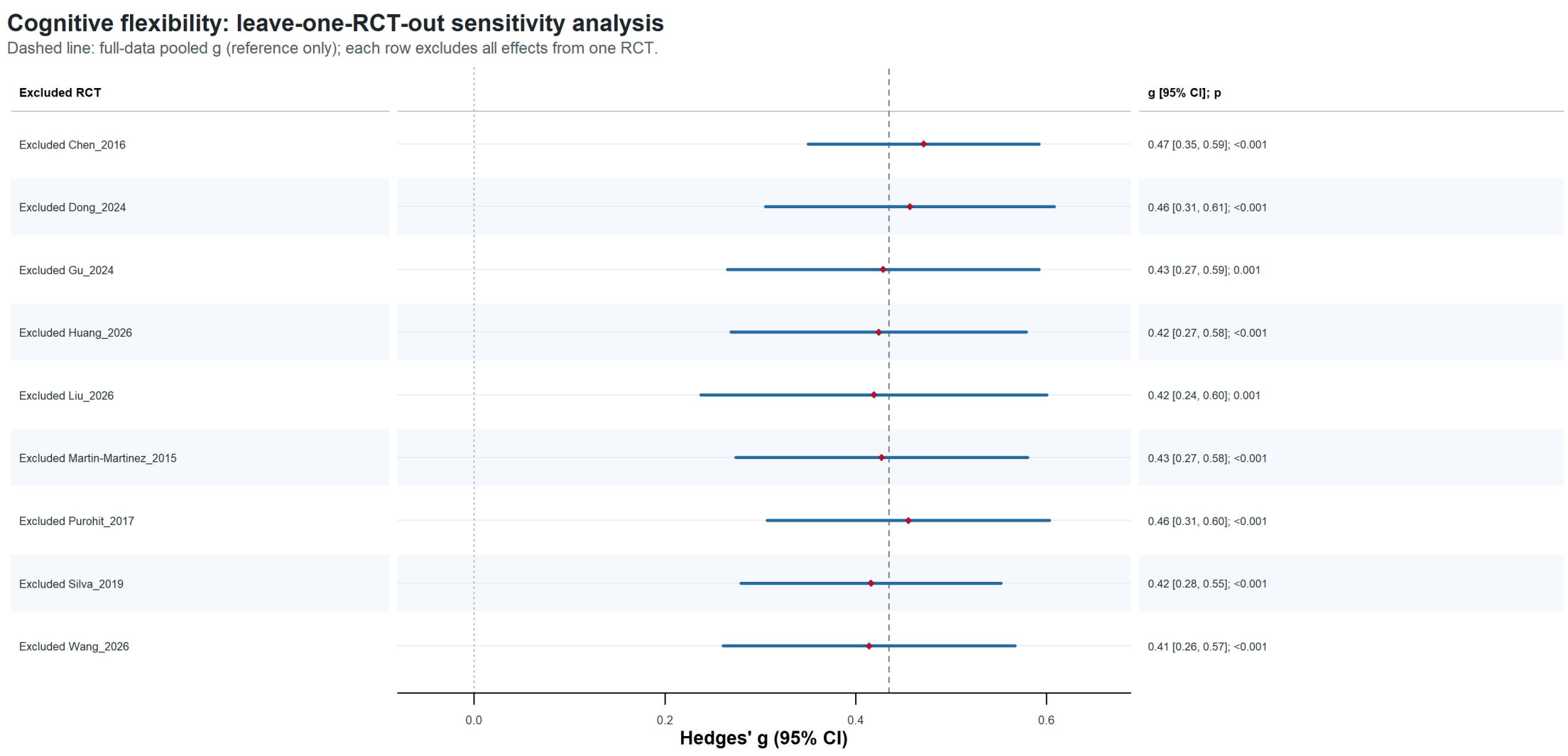

Figure S9. Leave-one-RCT-out sensitivity analysis for cognitive flexibility.

6.2 Sensitivity analysis excluding high-risk-of-bias results

Outcome-specific sensitivity analyses were performed after removing results judged to be at high overall risk of bias using RoB 2. The excluded high-risk RCTs differed by outcome because risk-of-bias judgments were made at the outcome level. Excluding these results did not materially alter the pooled effect estimates or their statistical significance, supporting the robustness of the main findings to the highest-risk evidence (Table S9; Figures S10–S13).

Table S9. Sensitivity analysis excluding high-risk-of-bias results.

**Outcome**	**High-risk RCTs excluded**	**Primary Hedges' g (95% CI)**	**After exclusion Hedges' g (95% CI)**	**p-value**	**τ^2 Level 2 / Level 3**	**I^2 Level 2 / Level 3 (%)**
**Overall**	Dong_2024, Silva_2019	0.55 (0.33–0.77)	0.55 (0.32–0.78)	<0.001	0.04 / 0.17	17.19 / 67.66
**Inhibitory control**	Dong_2024	0.56 (0.28–0.84)	0.56 (0.26–0.87)	0.001	0.04 / 0.24	12.38 / 73.84
**Working memory**	Dong_2024	0.51 (0.18–0.84)	0.59 (0.29–0.89)	0.002	<0.01 / 0.11	<0.01 / 73.39

**Cognitive flexibility**	Dong_2024, Silva_2019	0.43 (0.30–0.57)	0.44 (0.29–0.59)	<0.001	0.00 / <0.01	0.00 / <0.01

Note. The sensitivity analysis excluded trial–domain results judged to be at high overall risk of bias for the corresponding outcome. p-values and heterogeneity estimates refer to the model after exclusion. Level 2 represents residual heterogeneity among effect sizes within trials, and Level 3 represents between-trial heterogeneity. The evidence bases after exclusion were 20 RCTs/25 comparisons/79 effects for overall executive function, 16/20/45 for inhibitory control, 9/12/23 for working memory, and 7/9/11 for cognitive flexibility.

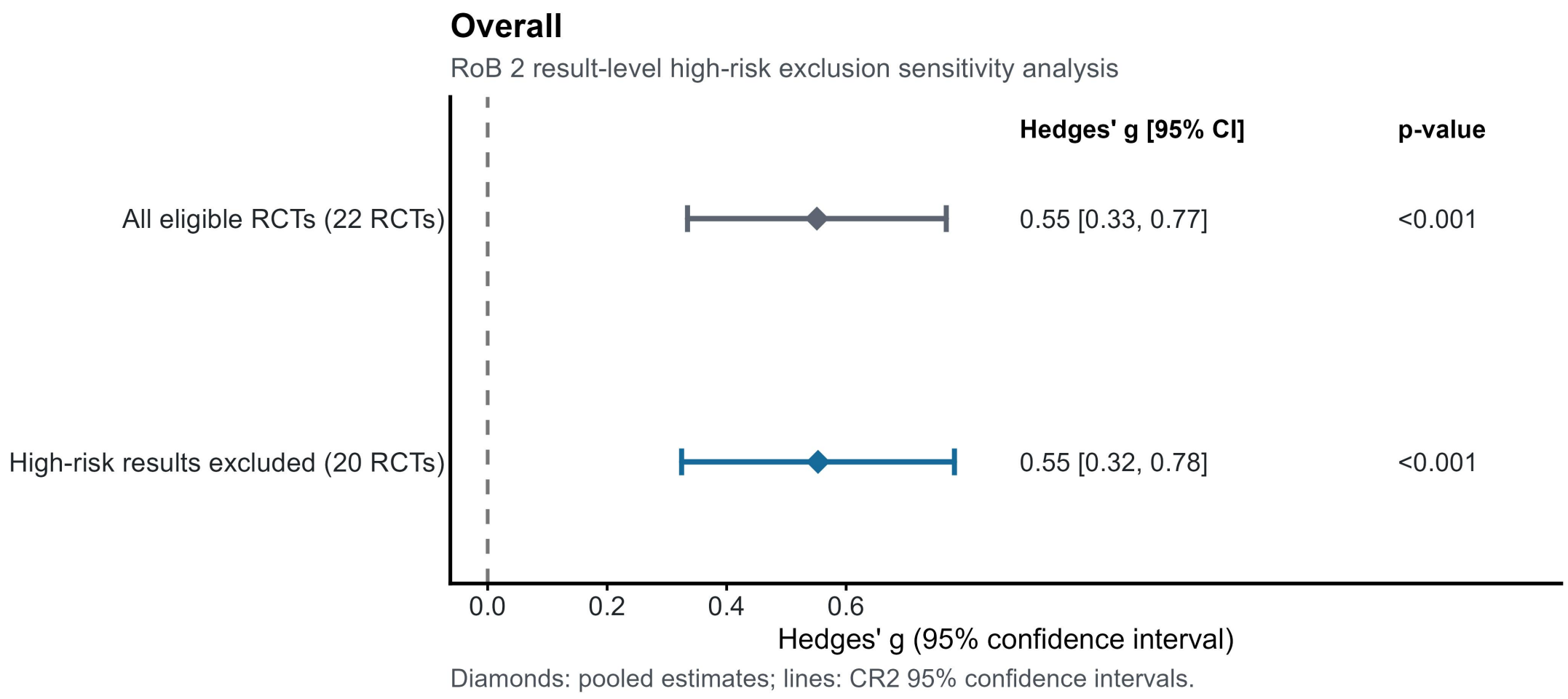

Figure S10. Sensitivity analysis excluding results judged at high risk of bias for overall executive function.

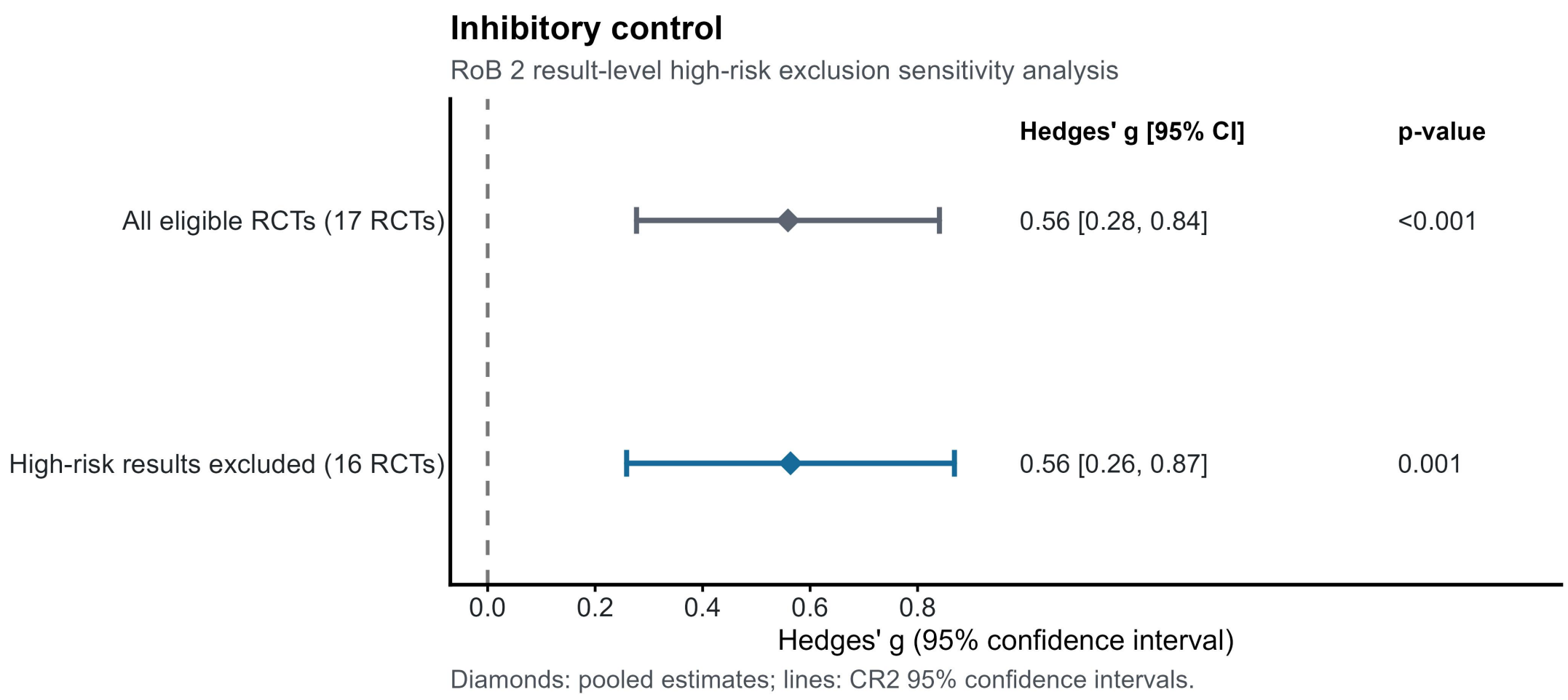

Figure S11. Sensitivity analysis excluding high-risk-of-bias studies for inhibitory control.

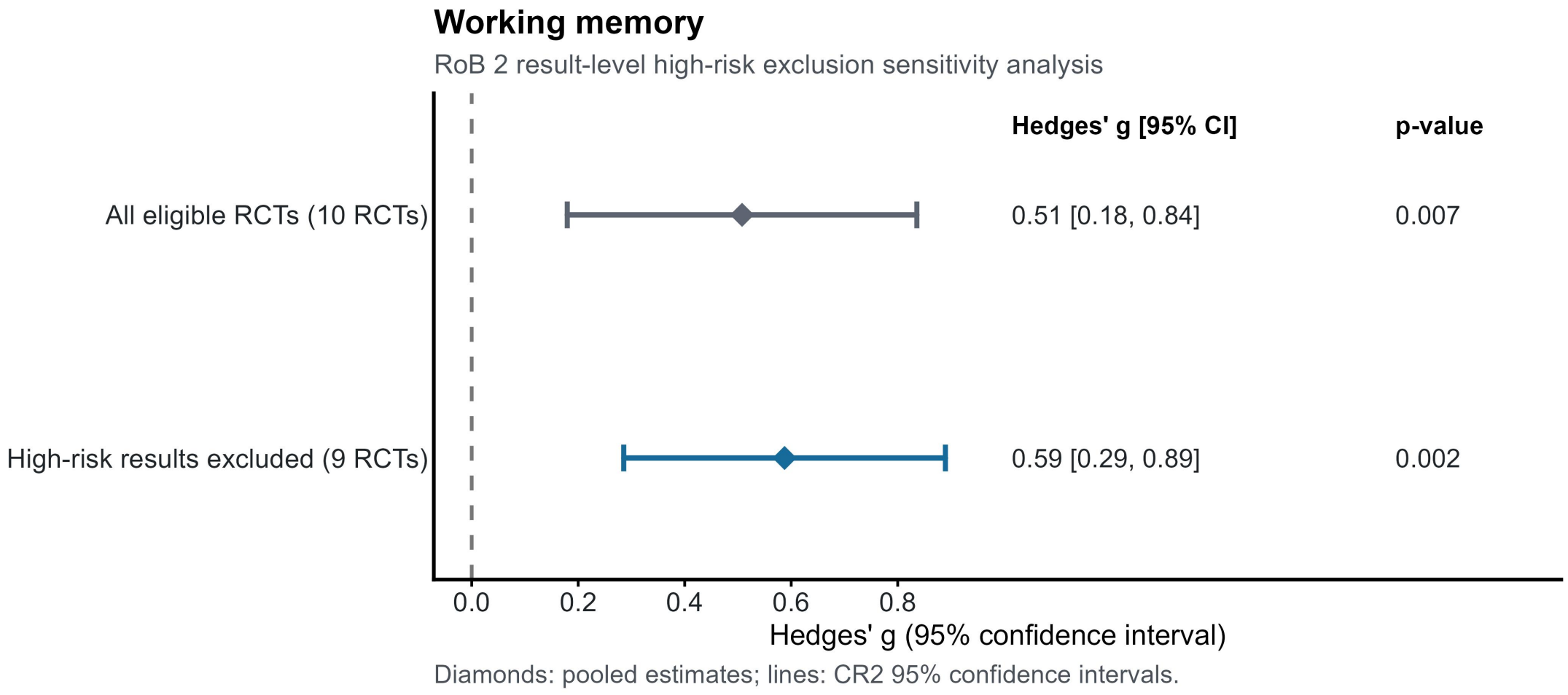

Figure S12. Sensitivity analysis excluding high-risk-of-bias studies for working memory.

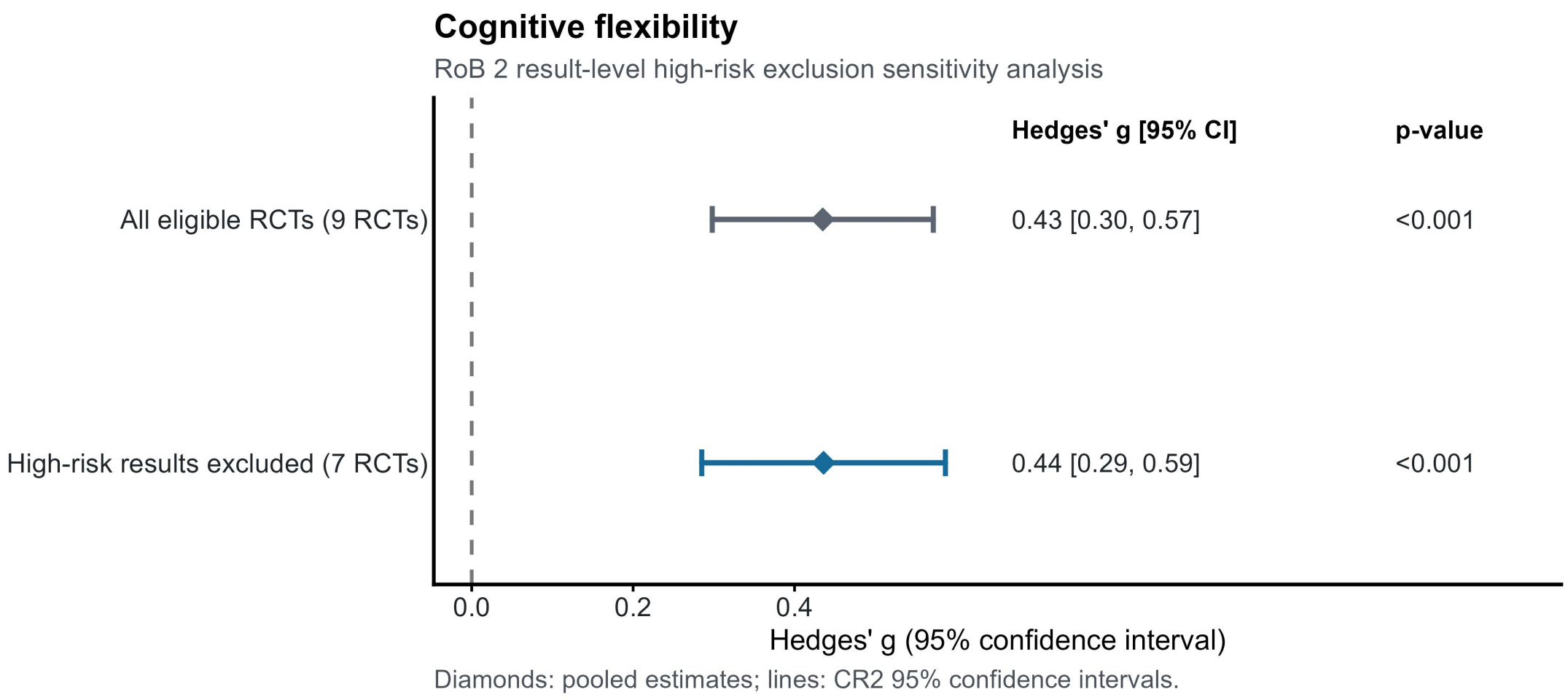

Figure S13. Sensitivity analysis excluding high-risk-of-bias studies for cognitive flexibility.

6.3 Task-classification sensitivity analysis

A reviewer-focused task-classification sensitivity analysis was conducted to examine whether the pooled conclusions depended on outcomes whose executive-function domain assignment was potentially debatable. Eleven prespecified scores were excluded in the sensitivity set: six Posner scores, three Digit Span total scores, and two raw Trail Making Test B time scores. The primary analysis retained all 86 effect sizes, whereas the sensitivity set retained 75 effect sizes from 20 RCTs. Table S10 documents the coding rationale for every extracted score, and Figures S14–S17 show the re-estimated pooled effects after the reviewer-focused exclusions.

Table S10. Executive-function outcome coding and reviewer-focused

task-classification sensitivity analysis.

No.	Study / arm	EF domain	Task / exact extracted score	Coding rationale and construct overlap	Domain specificity	Reviewer-focused concern (affected scores)	Decision
1	Cai_2025	Working memory	N-back 1-back RT (ms; sex subgroups combined)	Active maintenance and updating. Each stimulus is compared with the one presented one trial earlier; this condition imposes relatively low working-memory demands. Reaction time also reflects processing speed.	Low–moderate	—	Retain
2	Cai_2025	Working memory	N-back 2-back RT (ms; sex subgroups combined)	Active maintenance and updating. Each stimulus is compared with the one presented two trials earlier; this condition imposes greater working-memory demands than 1-back. Reaction time also reflects processing speed.	Moderate–high	—	Retain
3	Chen_2016	Cognitive flexibility	Wisconsin Card Sorting Test WCST nonperseverative errors (score)	Rule learning/set shifting (mixed). WCST rule changes require shifting, but nonperseverative errors are not a specific index of set-shifting failure or perseveration.	Low–moderate	—	Retain
4	Chen_2016	Cognitive flexibility	Wisconsin Card Sorting Test WCST total errors (score)	Rule learning/set shifting (mixed). WCST rule changes require shifting, but total errors combine perseverative and other errors and are not a process-pure shifting measure.	Low–moderate	—	Retain
5	Cho_2017	Inhibitory control	Stroop Color–Word Stroop Color-Word (score)	Color–word conflict plus reading/processing speed. The Color–Word condition involves interference control, but the raw score is not an adjusted interference-cost measure.	Moderate	—	Retain
6	Cho_2017a	Inhibitory control	Stroop Color–Word Stroop Color-Word (score)	Color–word conflict plus reading/processing speed. The Color–Word condition involves interference control, but the raw score is not an adjusted interference-cost measure.	Moderate	—	Retain
7	Chou_2020	Inhibitory control	Stroop Stroop color-word	Conflict/interference control. An interference score/condition reflects suppression of competing word	Moderate–high	—	Retain

No.	Study / arm	EF domain	Task / exact extracted score	Coding rationale and construct overlap	Domain specificity	Reviewer-focused concern (affected scores)	Decision
			interference RT (s)	information, but the metric may represent either a difference score or condition-specific RT.			
8	Chou_2020	Inhibitory control	Stroop Stroop incongruent color-naming RT (s)	Conflict control plus general processing speed. Incongruent trials require resolving word–color conflict, but raw RT also reflects general processing and response speed.	Moderate	—	Retain
9	Chou_2020	Inhibitory control	Stroop Stroop incongruent word-reading RT (s)	Conflict control plus general processing speed. Incongruent trials require resolving word–color conflict, but raw RT also reflects general processing and response speed.	Moderate	—	Retain
10	Chou_2020	Inhibitory control	Stroop Stroop word-color interference RT (s)	Conflict/interference control. An interference score/condition reflects suppression of competing word information, but the metric may represent either a difference score or condition-specific RT.	Moderate–high	—	Retain
11	Chou_2022a	Inhibitory control	Stroop Stroop incongruent color-naming RT (s)	Conflict control plus general processing speed. Incongruent trials require resolving word–color conflict, but raw RT also reflects general processing and response speed.	Moderate	—	Retain
12	Chou_2022a	Inhibitory control	Stroop Stroop incongruent word-reading RT (s)	Conflict control plus general processing speed. Incongruent trials require resolving word–color conflict, but raw RT also reflects general processing and response speed.	Moderate	—	Retain
13	Chou_2022a	Inhibitory control	Stroop Stroop interference color-naming RT (s)	Conflict/interference control. An interference score/condition reflects suppression of competing word information, but the metric may represent either a difference score or condition-specific RT.	Moderate–high	—	Retain
14	Chou_2022a	Inhibitory control	Stroop Stroop interference word-reading RT (s)	Conflict/interference control. An interference score/condition reflects suppression of competing word information, but the metric may represent either a difference score or	Moderate–high	—	Retain

No.	Study / arm	EF domain	Task / exact extracted score	Coding rationale and construct overlap	Domain specificity	Reviewer-focused concern (affected scores)	Decision
				condition-specific RT.			
15	Chou_2022b	Inhibitory control	Stroop Stroop incongruent color-naming RT (s)	Conflict control plus general processing speed. Incongruent trials require resolving word–color conflict, but raw RT also reflects general processing and response speed.	Moderate	—	Retain
16	Chou_2022b	Inhibitory control	Stroop Stroop incongruent word-reading RT (s)	Conflict control plus general processing speed. Incongruent trials require resolving word–color conflict, but raw RT also reflects general processing and response speed.	Moderate	—	Retain
17	Chou_2022b	Inhibitory control	Stroop Stroop interference color-naming RT (s)	Conflict/interference control. An interference score/condition reflects suppression of competing word information, but the metric may represent either a difference score or condition-specific RT.	Moderate–high	—	Retain
18	Chou_2022b	Inhibitory control	Stroop Stroop interference word-reading RT (s)	Conflict/interference control. An interference score/condition reflects suppression of competing word information, but the metric may represent either a difference score or condition-specific RT.	Moderate–high	—	Retain
19	Dong_2024	Cognitive flexibility	Task switching Shifting heterogeneous ACC (%)	Mixed-block rule switching. The heterogeneous condition requires alternating between rules; raw ACC/RT also includes non-switch performance and general processing speed.	Moderate	—	Retain
20	Dong_2024	Cognitive flexibility	Task switching Shifting heterogeneous RT (ms)	Mixed-block rule switching. The heterogeneous condition requires alternating between rules; raw ACC/RT also includes non-switch performance and general processing speed.	Moderate	—	Retain
21	Dong_2024	Inhibitory control	Eriksen flanker Flanker incongruent ACC (%)	Distractor resistance plus processing speed. Incongruent trials require ignoring flankers, but raw ACC/RT does not isolate an adjusted interference contrast.	Moderate	—	Retain

No.	Study / arm	EF domain	Task / exact extracted score	Coding rationale and construct overlap	Domain specificity	Reviewer-focused concern (affected scores)	Decision
22	Dong_2024	Inhibitory control	Eriksen flanker Flanker incongruent RT (ms)	Distractor resistance plus processing speed. Incongruent trials require ignoring flankers, but raw ACC/RT does not isolate an adjusted interference contrast.	Moderate	—	Retain
23	Dong_2024	Working memory	N-back 2-back ACC (%)	Active maintenance and updating. Each stimulus is compared with the one presented two trials earlier; this condition imposes greater working-memory demands than 1-back. Accuracy may also reflect sustained attention.	Moderate–high	—	Retain
24	Dong_2024	Working memory	N-back 2-back RT (ms)	Active maintenance and updating. Each stimulus is compared with the one presented two trials earlier; this condition imposes greater working-memory demands than 1-back. Reaction time also reflects processing speed.	Moderate–high	—	Retain
25	Gu_2024a	Cognitive flexibility	Task-switching Task-switching switch IES (ms)	Set shifting plus speed/accuracy. The study reports switch and non-switch IES separately; the extracted value is the switch condition rather than a switch-minus-repeat cost.	Moderate	—	Retain
26	Gu_2024a	Inhibitory control	Eriksen flanker Flanker incongruent IES (ms)	Distractor resistance plus processing speed. Incongruent trials require ignoring flankers, but raw ACC/RT does not isolate an adjusted interference contrast.	Moderate	—	Retain
27	Gu_2024a	Working memory	N-back 1-back IES (ms)	Active maintenance and updating. Each stimulus is compared with the one presented one trial earlier; this condition imposes relatively low working-memory demands. The inverse-efficiency score incorporates both accuracy and response speed.	Low–moderate	—	Retain
28	Gu_2024a	Working memory	N-back 2-back IES (ms)	Active maintenance and updating. Each stimulus is compared with the one presented two trials earlier; this condition imposes greater working-memory demands than	Moderate–high	—	Retain

No.	Study / arm	EF domain	Task / exact extracted score	Coding rationale and construct overlap	Domain specificity	Reviewer-focused concern (affected scores)	Decision
				1-back. The inverse-efficiency score incorporates both accuracy and response speed.			
29	Gu_2024a	Working memory	N-back 3-back IES (ms)	Active maintenance and updating. Each stimulus is compared with the one presented three trials earlier; this condition imposes greater working-memory demands than 1-back or 2-back. The inverse-efficiency score incorporates both accuracy and response speed.	Moderate–high	—	Retain
30	Gu_2024b	Cognitive flexibility	Task-switching Task-switching switch IES (ms)	Set shifting plus speed/accuracy. The study reports switch and non-switch IES separately; the extracted value is the switch condition rather than a switch-minus-repeat cost.	Moderate	—	Retain
31	Gu_2024b	Inhibitory control	Eriksen flanker Flanker incongruent IES (ms)	Distractor resistance plus processing speed. Incongruent trials require ignoring flankers, but raw ACC/RT does not isolate an adjusted interference contrast.	Moderate	—	Retain
32	Gu_2024b	Working memory	N-back 1-back IES (ms)	Active maintenance and updating. Each stimulus is compared with the one presented one trial earlier; this condition imposes relatively low working-memory demands. The inverse-efficiency score incorporates both accuracy and response speed.	Low–moderate	—	Retain
33	Gu_2024b	Working memory	N-back 2-back IES (ms)	Active maintenance and updating. Each stimulus is compared with the one presented two trials earlier; this condition imposes greater working-memory demands than 1-back. The inverse-efficiency score incorporates both accuracy and response speed.	Moderate–high	—	Retain
34	Gu_2024b	Working memory	N-back 3-back IES (ms)	Active maintenance and updating. Each stimulus is compared with the one presented three trials earlier; this condition imposes greater working-memory demands than	Moderate–high	—	Retain

No.	Study / arm	EF domain	Task / exact extracted score	Coding rationale and construct overlap	Domain specificity	Reviewer-focused concern (affected scores)	Decision
				1-back or 2-back. The inverse-efficiency score incorporates both accuracy and response speed.			
35	Jeong_2021	Working memory	Sternberg digit memory Sternberg easy ACC (%)	Short-term maintenance/retrieval. The probe is compared with a memorized digit set; higher load increases memory demand, while RT also reflects retrieval and response speed.	Low–moderate	—	Retain
36	Jeong_2021	Working memory	Sternberg digit memory Sternberg easy RT (ms)	Short-term maintenance/retrieval. The probe is compared with a memorized digit set; higher load increases memory demand, while RT also reflects retrieval and response speed.	Low–moderate	—	Retain
37	Jeong_2021	Working memory	Sternberg digit memory Sternberg hard ACC (%)	Short-term maintenance/retrieval. The probe is compared with a memorized digit set; higher load increases memory demand, while RT also reflects retrieval and response speed.	Moderate	—	Retain
38	Jeong_2021	Working memory	Sternberg digit memory Sternberg hard RT (ms)	Short-term maintenance/retrieval. The probe is compared with a memorized digit set; higher load increases memory demand, while RT also reflects retrieval and response speed.	Moderate	—	Retain
39	Kadri_2019	Inhibitory control	Stroop Color–Word Stroop Color-Word Interference Test (score)	Color–word conflict plus reading/processing speed. The Color–Word condition involves interference control, but the raw score is not an adjusted interference-cost measure.	Moderate	—	Retain
40	Kadri_2019	Inhibitory control	Stroop Stroop errors (count)	Conflict control plus task accuracy. Error counts may reflect failed conflict resolution, but the reported total is not a pure inhibitory-control measure.	Moderate	—	Retain
41	Kadri_2019	Inhibitory control	Stroop Stroop interference (score)	Conflict/interference control. An interference score/condition reflects suppression of competing word information, but the metric may represent either a difference score or condition-specific RT.	Moderate–high	—	Retain

No.	Study / arm	EF domain	Task / exact extracted score	Coding rationale and construct overlap	Domain specificity	Reviewer-focused concern (affected scores)	Decision
42	Liu_2018	Inhibitory control	Food-cue Stroop Stroop food-cue RT (s)	Food-related selective attention. Food words elicit attentional salience; this differs from the standard incongruent–congruent color–word interference contrast.	Low for inhibition	—	Retain
43	Liu_2018	Inhibitory control	Stroop Stroop incongruent RT (s)	Conflict control plus general processing speed. Incongruent trials require resolving word–color conflict, but raw RT also reflects general processing and response speed.	Moderate	—	Retain
44	Liu_2025	Inhibitory control	Posner spatial-cue task Posner invalid ACC (proportion)	Spatial attention/orienting. This condition requires spatial reorienting after a misleading cue; the score is not an invalid–valid cost or a response-inhibition contrast.	Low for inhibitory control	Posner task: spatial attention/orienting rather than specific inhibitory control (6 scores; 1 RCT)	Exclude
45	Liu_2025	Inhibitory control	Posner spatial-cue task Posner invalid RT (ms)	Spatial attention/orienting. This condition requires spatial reorienting after a misleading cue; the score is not an invalid–valid cost or a response-inhibition contrast.	Low for inhibitory control	Posner task: spatial attention/orienting rather than specific inhibitory control (6 scores; 1 RCT)	Exclude
46	Liu_2025	Inhibitory control	Posner spatial-cue task Posner neutral ACC (proportion)	Spatial attention/orienting. This condition indexes target detection without directional cueing; the score is not an invalid–valid cost or a response-inhibition contrast.	Low for inhibitory control	Posner task: spatial attention/orienting rather than specific inhibitory control (6 scores; 1 RCT)	Exclude
47	Liu_202	Inhibitory	Posner	Spatial attention/orienting. This	Low	Posner task:	Exclud

No.	Study / arm	EF domain	Task / exact extracted score	Coding rationale and construct overlap	Domain specificity	Reviewer-focused concern (affected scores)	Decision
	5	control	spatial-cue task Posner neutral RT (ms)	condition indexes target detection without directional cueing; the score is not an invalid–valid cost or a response-inhibition contrast.	for inhibitory control	spatial attention/orienting rather than specific inhibitory control (6 scores; 1 RCT)	e
48	Liu_2025	Inhibitory control	Posner spatial-cue task Posner valid ACC (proportion)	Spatial attention/orienting. This condition indexes facilitated spatial orienting; the score is not an invalid–valid cost or a response-inhibition contrast.	Low for inhibitory control	Posner task: spatial attention/orienting rather than specific inhibitory control (6 scores; 1 RCT)	Exclude
49	Liu_2025	Inhibitory control	Posner spatial-cue task Posner valid RT (ms)	Spatial attention/orienting. This condition indexes facilitated spatial orienting; the score is not an invalid–valid cost or a response-inhibition contrast.	Low for inhibitory control	Posner task: spatial attention/orienting rather than specific inhibitory control (6 scores; 1 RCT)	Exclude
50	Martin-Martinez_2015	Working memory	Digit Span Digit Span (score)	Storage plus manipulation (mixed). Forward recall primarily indexes span, whereas backward recall adds manipulation; the combined score cannot separate these components.	Mixed	Digit Span total score: short-term storage versus working-memory manipulation (3 scores; 2 RCTs)	Exclude
51	Martin-Martinez_2015	Working memory	Letter–Number Sequencing Letter-Number	Maintenance plus manipulation. Reordering mixed letters and numbers requires active maintenance, updating,	High	—	Retain

No.	Study / arm	EF domain	Task / exact extracted score	Coding rationale and construct overlap	Domain specificity	Reviewer-focused concern (affected scores)	Decision
			Sequencing (score)	and manipulation rather than simple repetition.			
52	Martin-Martinez_2015	Cognitive flexibility	Trail Making Test B Trail Making Test B (s)	Set shifting plus visual-search/motor speed. Alternating numbers and letters requires shifting, but raw completion time also reflects visual search, processing speed, and motor speed.	Moderate	Raw TMT-B time: set shifting versus visual search, processing speed, and motor speed (2 scores; 2 RCTs)	Exclude
53	Martin-Martinez_2015	Inhibitory control	Stroop Color–Word Stroop Word-Color (score)	Color–word conflict plus reading/processing speed. The Color–Word condition involves interference control, but the raw score is not an adjusted interference-cost measure.	Moderate	—	Retain
54	Purohit_2017	Cognitive flexibility	Trail Making Test B Trail Making Test B (s)	Set shifting plus visual-search/motor speed. Alternating numbers and letters requires shifting, but raw completion time also reflects visual search, processing speed, and motor speed.	Moderate	Raw TMT-B time: set shifting versus visual search, processing speed, and motor speed (2 scores; 2 RCTs)	Exclude
55	Purohit_2017	Inhibitory control	Stroop Color–Word Stroop Color-Word (score)	Color–word conflict plus reading/processing speed. The Color–Word condition involves interference control, but the raw score is not an adjusted interference-cost measure.	Moderate	—	Retain
56	Purohit_2017	Working memory	Digit Span Backward Digit Span	Maintenance plus manipulation. Reversing digit order requires maintaining and manipulating the	High	—	Retain

No.	Study / arm	EF domain	Task / exact extracted score	Coding rationale and construct overlap	Domain specificity	Reviewer-focused concern (affected scores)	Decision
			Backward (score)	sequence.			
57	Roh_2018	Inhibitory control	Stroop Color–Word Stroop Color-Word (score)	Color–word conflict plus reading/processing speed. The Color–Word condition involves interference control, but the raw score is not an adjusted interference-cost measure.	Moderate	—	Retain
58	Silva_2019	Cognitive flexibility	Trails test, Parts A/B Trail test cognitive flexibility (score)	Alternation with baseline adjustment. The study reports a hits-based B−A score rather than conventional TMT-B completion time; alternation is represented, but comparability with standard TMT-B metrics is limited.	Moderate; nonstandard metric	—	Retain
59	Torbeyns_2017	Inhibitory control	Stroop Stroop color-incongruent ACC (%)	Conflict control plus general processing speed. Incongruent trials require resolving word–color conflict, but raw RT also reflects general processing and response speed.	Moderate	—	Retain
60	Torbeyns_2017	Inhibitory control	Stroop Stroop color-incongruent RT (ms)	Conflict control plus general processing speed. Incongruent trials require resolving word–color conflict, but raw RT also reflects general processing and response speed.	Moderate	—	Retain
61	Torbeyns_2017	Inhibitory control	Stroop Stroop word-incongruent ACC (%)	Conflict control plus general processing speed. Incongruent trials require resolving word–color conflict, but raw RT also reflects general processing and response speed.	Moderate	—	Retain
62	Torbeyns_2017	Inhibitory control	Stroop Stroop word-incongruent RT (ms)	Conflict control plus general processing speed. Incongruent trials require resolving word–color conflict, but raw RT also reflects general processing and response speed.	Moderate	—	Retain
63	Wang_2026a	Cognitive flexibility	More–odd shifting More-odd shifting ACC (%)	Mixed-rule accuracy. Overall accuracy is reported across task sections and therefore is not a switch-specific accuracy cost.	Low–moderate	—	Retain
64	Wang_2	Cognitive	More–odd	Set shifting. Methods define RT as the	High–	—	Retain

No.	Study / arm	EF domain	Task / exact extracted score	Coding rationale and construct overlap	Domain specificity	Reviewer-focused concern (affected scores)	Decision
	026a	flexibility	shifting More-odd shifting RT (ms)	switching-minus-non-switching difference, although the workbook label does not explicitly include 'cost'.	moderate		
65	Wang_2026a	Inhibitory control	Go/No-Go GO/NOGO RT (ms)	Go-response speed. The source reports RT for Go responses and No-Go accuracy separately; Go RT alone does not index successful response withholding.	Low for inhibition	—	Retain
66	Wang_2026a	Working memory	N-back 2-back ACC (%)	Active maintenance and updating. Each stimulus is compared with the one presented two trials earlier; this condition imposes greater working-memory demands than 1-back. Accuracy may also reflect sustained attention.	Moderate–high	—	Retain
67	Wang_2026a	Working memory	N-back 2-back RT (ms)	Active maintenance and updating. Each stimulus is compared with the one presented two trials earlier; this condition imposes greater working-memory demands than 1-back. Reaction time also reflects processing speed.	Moderate–high	—	Retain
68	Wang_2026b	Cognitive flexibility	More–odd shifting More-odd shifting ACC (%)	Mixed-rule accuracy. Overall accuracy is reported across task sections and therefore is not a switch-specific accuracy cost.	Low–moderate	—	Retain
69	Wang_2026b	Inhibitory control	Go/No-Go GO/NOGO ACC (%)	Response inhibition. Withholding a prepotent Go response on No-Go trials directly indexes response suppression.	High	—	Retain
70	Wang_2026b	Inhibitory control	Go/No-Go GO/NOGO RT (ms)	Go-response speed. The source reports RT for Go responses and No-Go accuracy separately; Go RT alone does not index successful response withholding.	Low for inhibition	—	Retain
71	Wang_2026b	Working memory	N-back 2-back ACC (%)	Active maintenance and updating. Each stimulus is compared with the one presented two trials earlier; this condition imposes greater	Moderate–high	—	Retain

No.	Study / arm	EF domain	Task / exact extracted score	Coding rationale and construct overlap	Domain specificity	Reviewer-focused concern (affected scores)	Decision
				working-memory demands than 1-back. Accuracy may also reflect sustained attention.			
72	Wang_2026b	Working memory	N-back 2-back RT (ms)	Active maintenance and updating. Each stimulus is compared with the one presented two trials earlier; this condition imposes greater working-memory demands than 1-back. Reaction time also reflects processing speed.	Moderate–high	—	Retain
73	Gejl_2024a	Inhibitory control	Eriksen flanker Flanker incongruent RT (ms)	Distractor resistance plus processing speed. Incongruent trials require ignoring flankers, but raw ACC/RT does not isolate an adjusted interference contrast.	Moderate	—	Retain
74	Gejl_2024a	Inhibitory control	Eriksen flanker Flanker interference RT (ms)	Conflict/interference control. An incongruent–congruent contrast more directly isolates distractor interference than a raw task-condition score.	High–moderate	—	Retain
75	Gejl_2024a	Inhibitory control	Eriksen flanker Flanker interference ACC (percentage-point cost)	Conflict/interference control. An incongruent–congruent contrast more directly isolates distractor interference than a raw task-condition score.	High–moderate	—	Retain
76	Gejl_2024b	Inhibitory control	Eriksen flanker Flanker incongruent RT (ms)	Distractor resistance plus processing speed. Incongruent trials require ignoring flankers, but raw ACC/RT does not isolate an adjusted interference contrast.	Moderate	—	Retain
77	Gejl_2024b	Inhibitory control	Eriksen flanker Flanker interference RT (ms)	Conflict/interference control. An incongruent–congruent contrast more directly isolates distractor interference than a raw task-condition score.	High–moderate	—	Retain
78	Gejl_2024b	Inhibitory control	Eriksen flanker Flanker interference ACC (percentage-point cost)	Conflict/interference control. An incongruent–congruent contrast more directly isolates distractor interference than a raw task-condition score.	High–moderate	—	Retain

No.	Study / arm	EF domain	Task / exact extracted score	Coding rationale and construct overlap	Domain specificity	Reviewer-focused concern (affected scores)	Decision
79	Babayoglu_2026a	Working memory	Visual–Auditory Digit Span Visual-Auditory Digit Span total (score)	Serial short-term storage. The total score combines auditory and visual reproduction modes; no backward or reordering condition is described.	Low for working-memory manipulation	Digit Span total score: short-term storage versus working-memory manipulation (3 scores; 2 RCTs)	Exclude
80	Babayoglu_2026b	Working memory	Visual–Auditory Digit Span Visual-Auditory Digit Span total (score)	Serial short-term storage. The total score combines auditory and visual reproduction modes; no backward or reordering condition is described.	Low for working-memory manipulation	Digit Span total score: short-term storage versus working-memory manipulation (3 scores; 2 RCTs)	Exclude
81	Huang_2026	Inhibitory control	Eriksen flanker Flanker conflict effect RT (ms)	Conflict/interference control. An incongruent–congruent contrast more directly isolates distractor interference than a raw task-condition score.	High–moderate	—	Retain
82	Huang_2026	Working memory	N-back 2-back RT (ms)	Active maintenance and updating. Each stimulus is compared with the one presented two trials earlier; this condition imposes greater working-memory demands than 1-back. Reaction time also reflects processing speed.	Moderate–high	—	Retain
83	Huang_2026	Cognitive flexibility	More–odd shifting More-odd shifting switch cost RT (ms)	Set shifting. The reported difference adjusts for non-switch response speed and directly quantifies switching cost.	High–moderate	—	Retain
84	Liu_2026	Inhibitory control	Eriksen flanker Flanker interference RT (ms)	Conflict/interference control. An incongruent–congruent contrast more directly isolates distractor interference than a raw task-condition score.	High–moderate	—	Retain
85	Liu_202	Working	N-back	Active maintenance and updating.	Moder	—	Retain

No.	Study / arm	EF domain	Task / exact extracted score	Coding rationale and construct overlap	Domain specificity	Reviewer-focused concern (affected scores)	Decision
	6	memory	2-back RT (ms)	Each stimulus is compared with the one presented two trials earlier; this condition imposes greater working-memory demands than 1-back. Reaction time also reflects processing speed.	ate–high		
86	Liu_2026	Cognitive flexibility	More–odd shifting More-odd shifting RT (ms)	Set shifting. The score is defined as the heterogeneous-minus-homogeneous RT difference, reducing contamination by general processing speed.	High–moderate	—	Retain

Note. Each extracted score was assigned to one primary EF domain for the main analysis. The reviewer-focused sensitivity set excluded 11 prespecified scores with potentially overlapping or debatable construct assignment: six Posner scores, three Digit Span total scores, and two raw TMT-B time scores. Counts in the "Reviewer-focused concern" column refer to the total number of affected scores within the task family and are repeated across corresponding rows; they should not be summed. The primary analysis retained all 86 effect sizes, whereas the sensitivity set retained 75 effect sizes from 20 RCTs. EF, executive function; RT, reaction time; ACC, accuracy; IES, inverse efficiency score; TMT-B, Trail Making Test Part B.

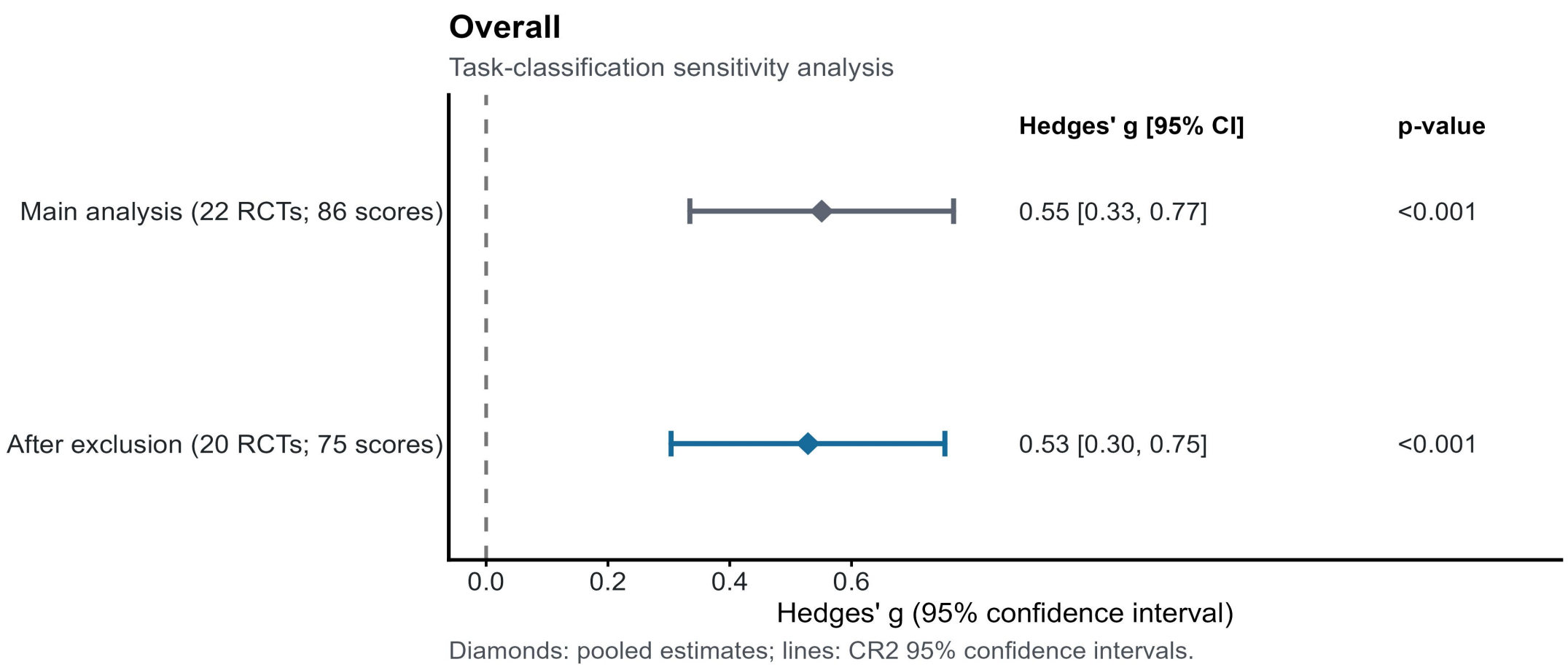

Figure S14. Task-classification sensitivity analysis for overall executive function.

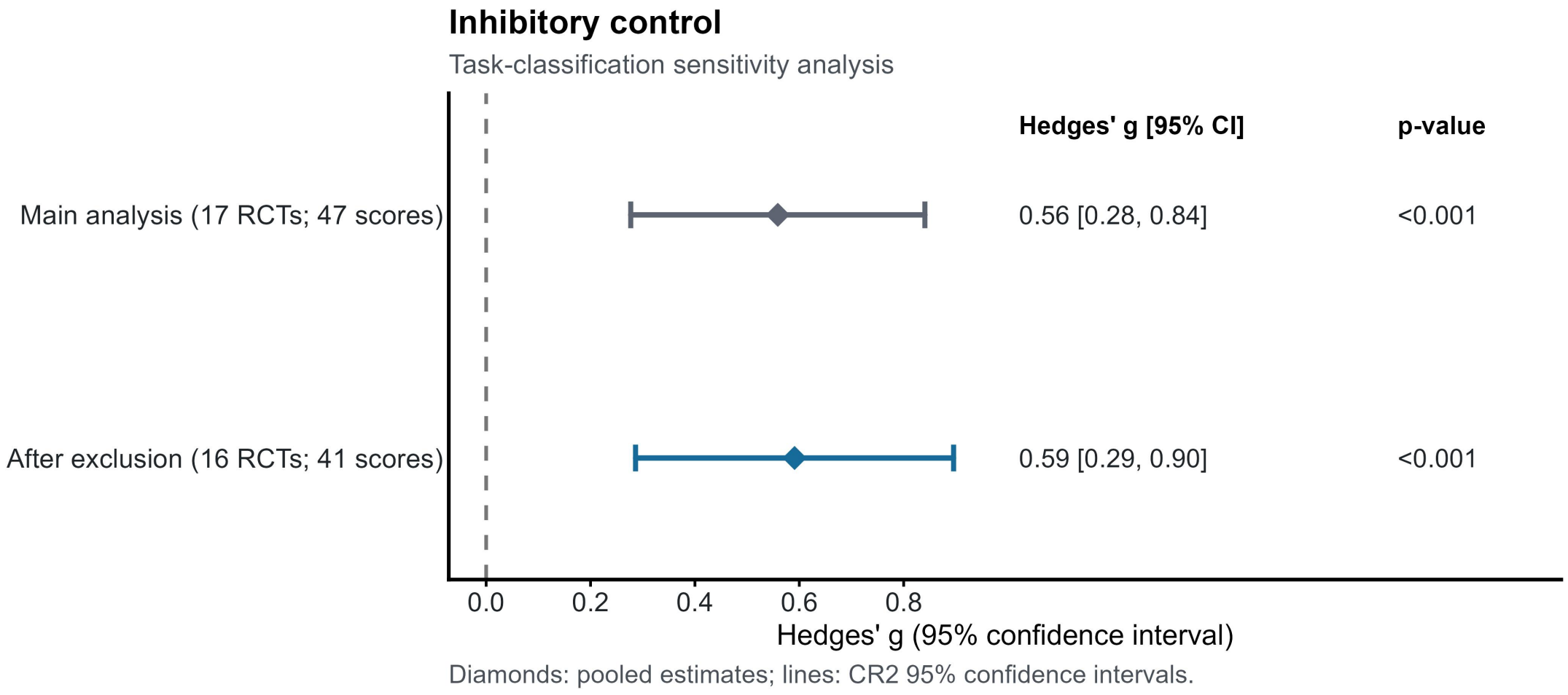

Figure S15. Task-classification sensitivity analysis for inhibitory control.

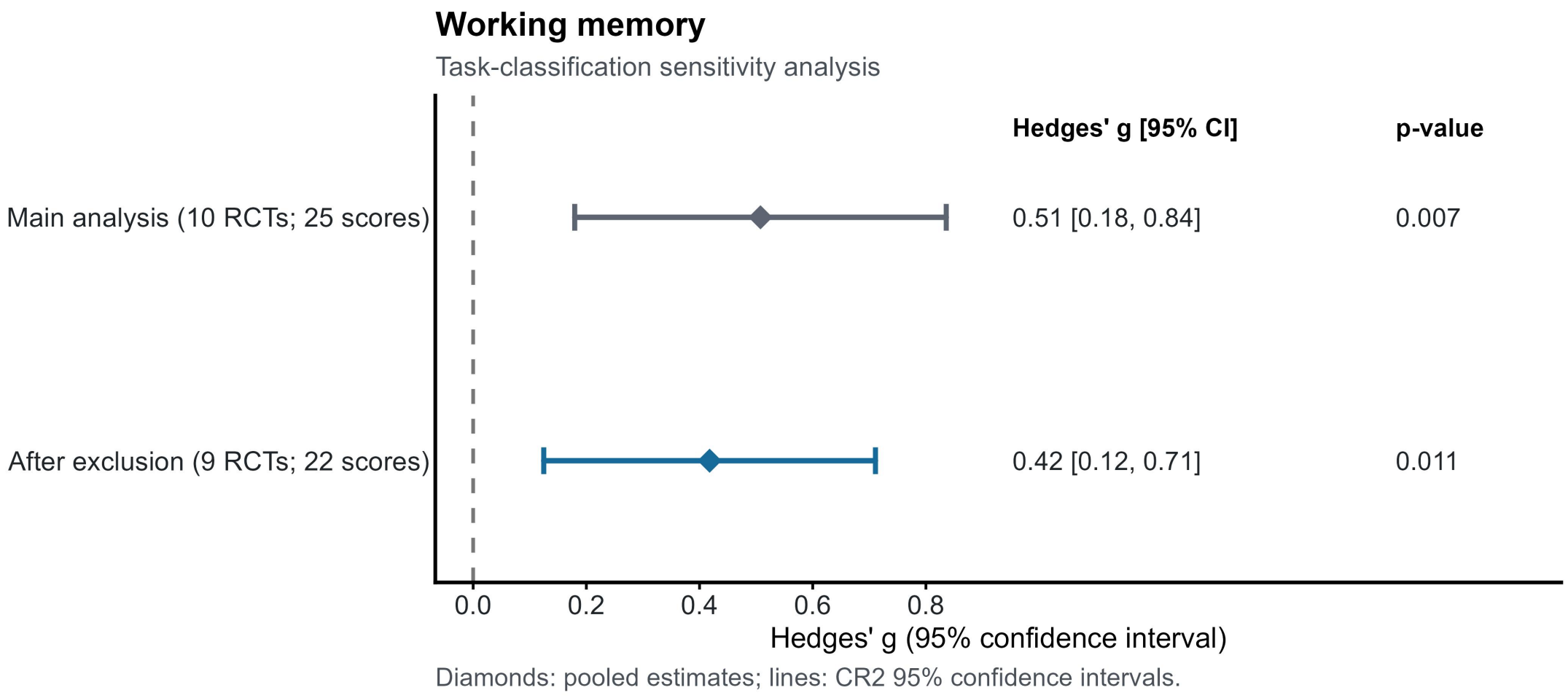

Figure S16. Task-classification sensitivity analysis for working memory.

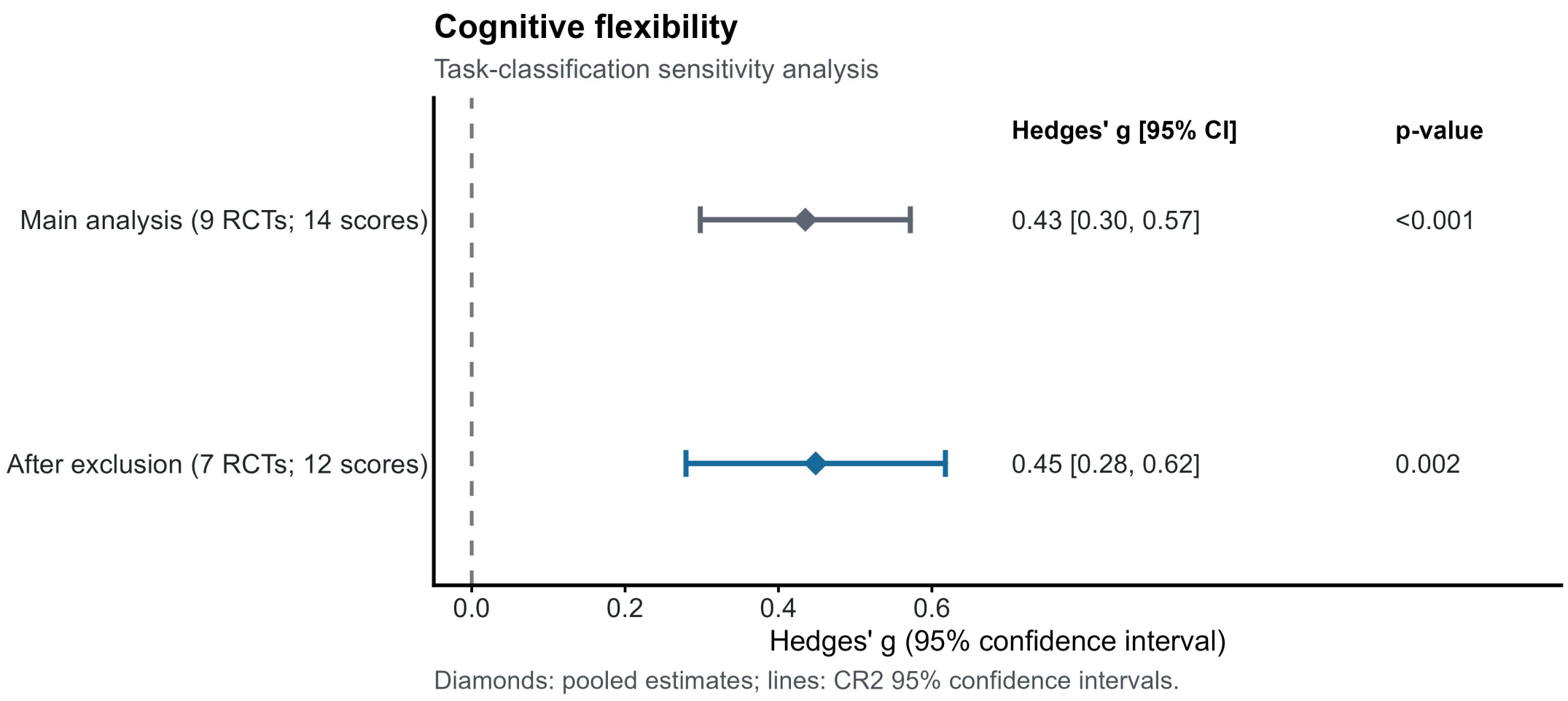

Figure S17. Task-classification sensitivity analysis for cognitive flexibility.

6.4 Correlation-assumption sensitivity analyses

The primary three-level models assumed a within-study outcome correlation of $\rho = 0.50$ and a pre–post correlation of $r = 0.50$ when change-score variances had to be derived. Sensitivity analyses varied one assumption at a time to 0.30 and 0.70 while holding the other at 0.50. Across all scenarios, the direction and statistical significance of the pooled effects were unchanged. Across the full set of correlation scenarios, Hedges' g ranged from 0.47 to 0.69 for overall executive function, 0.48 to 0.70 for inhibitory control, 0.44 to 0.63 for working memory, and 0.37 to 0.54 for cognitive flexibility (Table S11; Figures S18–S21).

Table S11. Sensitivity analyses for assumed within-study outcome correlation (ρ) and pre-post correlation (r)

Outcome	Assumption tested	RCTs / comparisons / effects	Hedges' g (95% CI); p	Δg vs primary	τ^2 Level 2 / Level 3	I^2 (%) Level 2 / Level 3 / Total
**Overall executive function**	**Primary r = 0.50; ρ = 0.50**	**22 / 27 / 86**	**0.55 (0.33 to 0.77); p < 0.001**	**+0.00**	**0.050 / 0.162**	**20.03 / 64.89 / 84.92**
	Outcome ρ = 0.30 (r = 0.50)	22 / 27 / 86	0.56 (0.33 to 0.78); p < 0.001	+0.01	0.036 / 0.191	12.90 / 69.14 / 82.04
	Outcome ρ = 0.70 (r = 0.50)	22 / 27 / 86	0.55 (0.34 to 0.76); p < 0.001	-0.01	0.070 / 0.134	30.90 / 58.66 / 89.57
	Pre/post r = 0.30 (ρ = 0.50)	22 / 27 / 86	0.47 (0.29 to 0.66); p < 0.001	-0.08	0.029 / 0.107	16.58 / 61.84 / 78.42

Outcome	Assumption tested	RCTs / comparisons / effects	Hedges' g (95% CI); p	Δg vs primary	τ² Level 2 / Level 3	I² (%) Level 2 / Level 3 / Total
	Pre/post r = 0.70 (ρ = 0.50)	22 / 27 / 86	0.69 (0.42 to 0.96); p < 0.001	+0.14	0.099 / 0.281	23.60 / 67.13 / 90.73
**Inhibitory control**	**Primary r = 0.50; ρ = 0.50**	**17 / 21 / 47**	**0.56 (0.28 to 0.84); p < 0.001**	**+0.00**	**0.034 / 0.219**	**11.55 / 73.38 / 84.93**
	Outcome ρ = 0.30 (r = 0.50)	17 / 21 / 47	0.56 (0.27 to 0.85); p < 0.001	+0.00	<0.001 / 0.254	<0.01 / 81.86 / 81.86
	Outcome ρ = 0.70 (r = 0.50)	17 / 21 / 47	0.56 (0.28 to 0.84); p < 0.001	+0.00	0.083 / 0.184	27.89 / 62.01 / 89.90
	Pre/post r = 0.30 (ρ = 0.50)	17 / 21 / 47	0.48 (0.24 to 0.72); p < 0.001	-0.08	0.008 / 0.154	3.77 / 74.77 / 78.54
	Pre/post r = 0.70 (ρ = 0.50)	17 / 21 / 47	0.70 (0.34 to 1.05); p < 0.001	+0.14	0.092 / 0.359	18.42 / 72.22 / 90.64

Outcome	Assumption tested	RCTs / comparisons / effects	Hedges' g (95% CI); p	Δg vs primary	τ² Level 2 / Level 3	I² (%) Level 2 / Level 3 / Total
**Working memory**	**Primary r = 0.50; ρ = 0.50**	**10 / 13 / 25**	**0.51 (0.18 to 0.84); p = 0.007**	**+0.00**	**<0.001 / 0.162**	**<0.01 / 80.67 / 80.67**
	Outcome ρ = 0.30 (r = 0.50)	10 / 13 / 25	0.50 (0.17 to 0.83); p = 0.007	-0.01	<0.001 / 0.166	<0.01 / 77.97 / 77.97
	Outcome ρ = 0.70 (r = 0.50)	10 / 13 / 25	0.51 (0.19 to 0.84); p = 0.006	+0.01	<0.001 / 0.157	<0.01 / 85.49 / 85.49
	Pre/post r = 0.30 (ρ = 0.50)	10 / 13 / 25	0.44 (0.16 to 0.72); p = 0.007	-0.07	<0.001 / 0.106	<0.01 / 73.49 / 73.49
	Pre/post r = 0.70 (ρ = 0.50)	10 / 13 / 25	0.63 (0.22 to 1.04); p = 0.007	+0.12	0.010 / 0.277	2.99 / 84.90 / 87.89
**Cognitive flexibility**	**Primary r = 0.50; ρ = 0.50**	**9 / 11 / 14**	**0.43 (0.30 to 0.57); p < 0.001**	**+0.00**	**<0.001 / <0.001**	**<0.01 / <0.01 / <0.01**

Outcome	Assumption tested	RCTs / comparisons / effects	Hedges' g (95% CI); p	Δg vs primary	τ^2 Level 2 / Level 3	I^2 (%) Level 2 / Level 3 / Total
	Outcome ρ = 0.30 (r = 0.50)	9 / 11 / 14	0.43 (0.29 to 0.57); p < 0.001	-0.01	0.000 / <0.001	0.00 / <0.01 / <0.01
	Outcome ρ = 0.70 (r = 0.50)	9 / 11 / 14	0.44 (0.30 to 0.58); p < 0.001	+0.00	0.006 / <0.001	12.18 / <0.01 / 12.18
	Pre/post r = 0.30 (ρ = 0.50)	9 / 11 / 14	0.37 (0.26 to 0.49); p < 0.001	-0.06	<0.001 / <0.001	<0.01 / <0.01 / <0.01
	Pre/post r = 0.70 (ρ = 0.50)	9 / 11 / 14	0.54 (0.36 to 0.72); p < 0.001	+0.10	0.008 / <0.001	13.35 / <0.01 / 13.35

Note. Each outcome includes the primary model plus four sensitivity scenarios. ρ is the assumed within-study correlation among outcome effect sizes, whereas r is the assumed pre-post correlation used to derive change-score variances. Only one correlation assumption was varied in each sensitivity scenario, with the other fixed at 0.50. Level 2 represents residual heterogeneity among effect sizes within trials and Level 3 represents between-trial heterogeneity. Three-level random-effects models were fitted by REML with trial-clustered CR2 confidence intervals and p-values.

Δg is the change in pooled Hedges' g relative to the primary model.

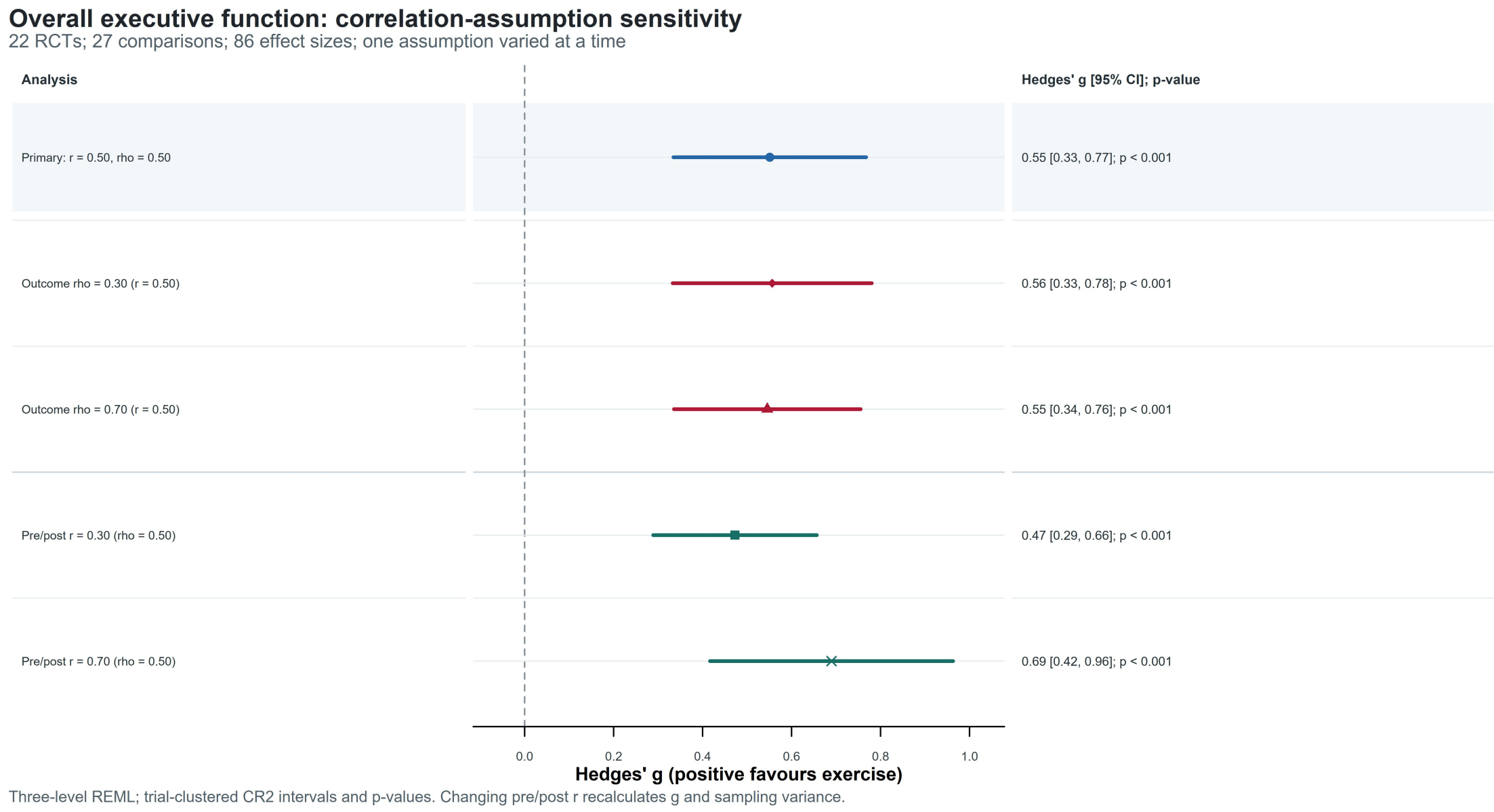

Figure S18. Sensitivity analyses of the pooled effect of exercise on overall executive function under alternative correlation assumptions

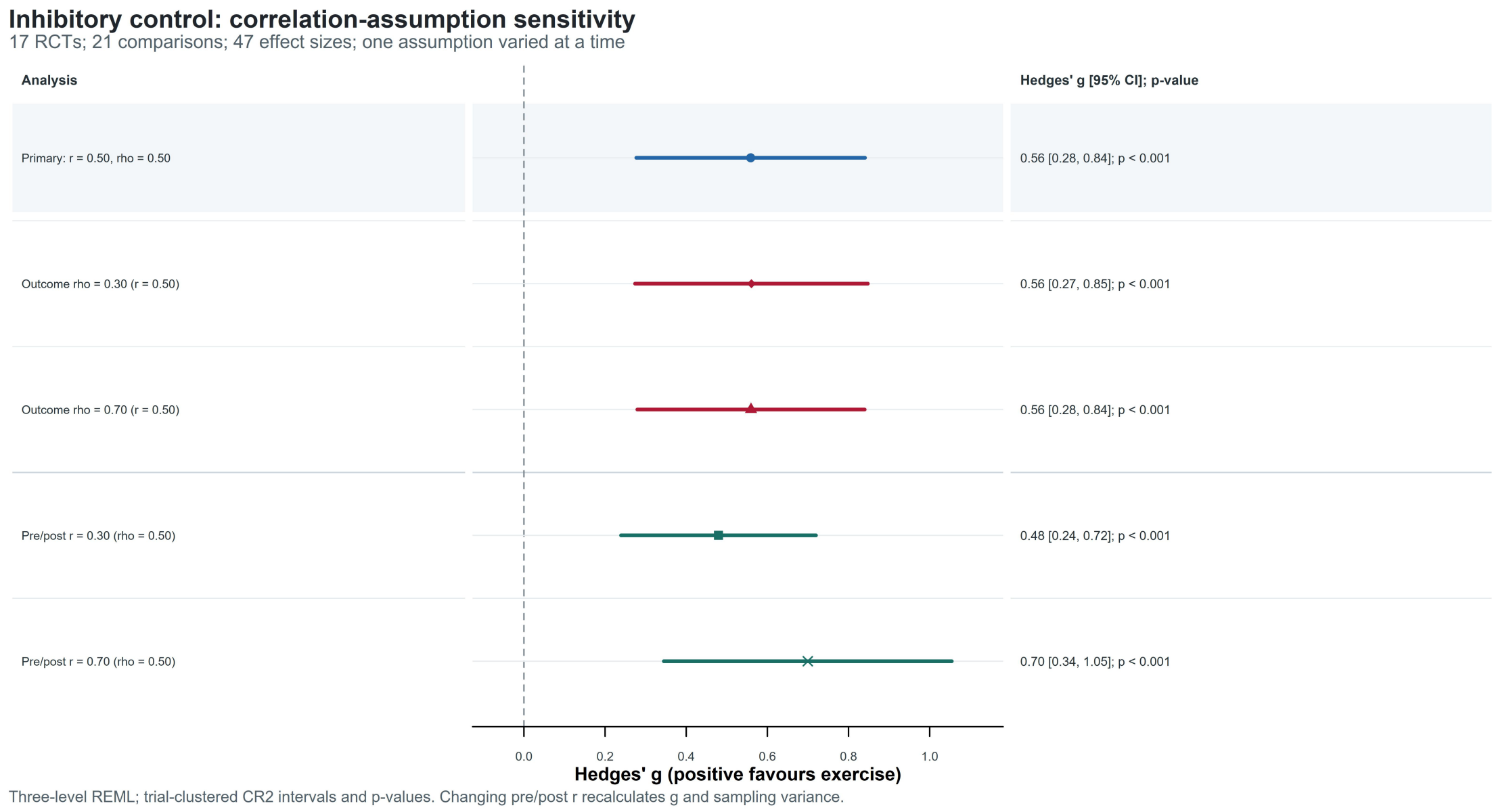

Figure S19. Sensitivity analyses of the pooled effect of exercise on inhibitory control under alternative correlation assumptions

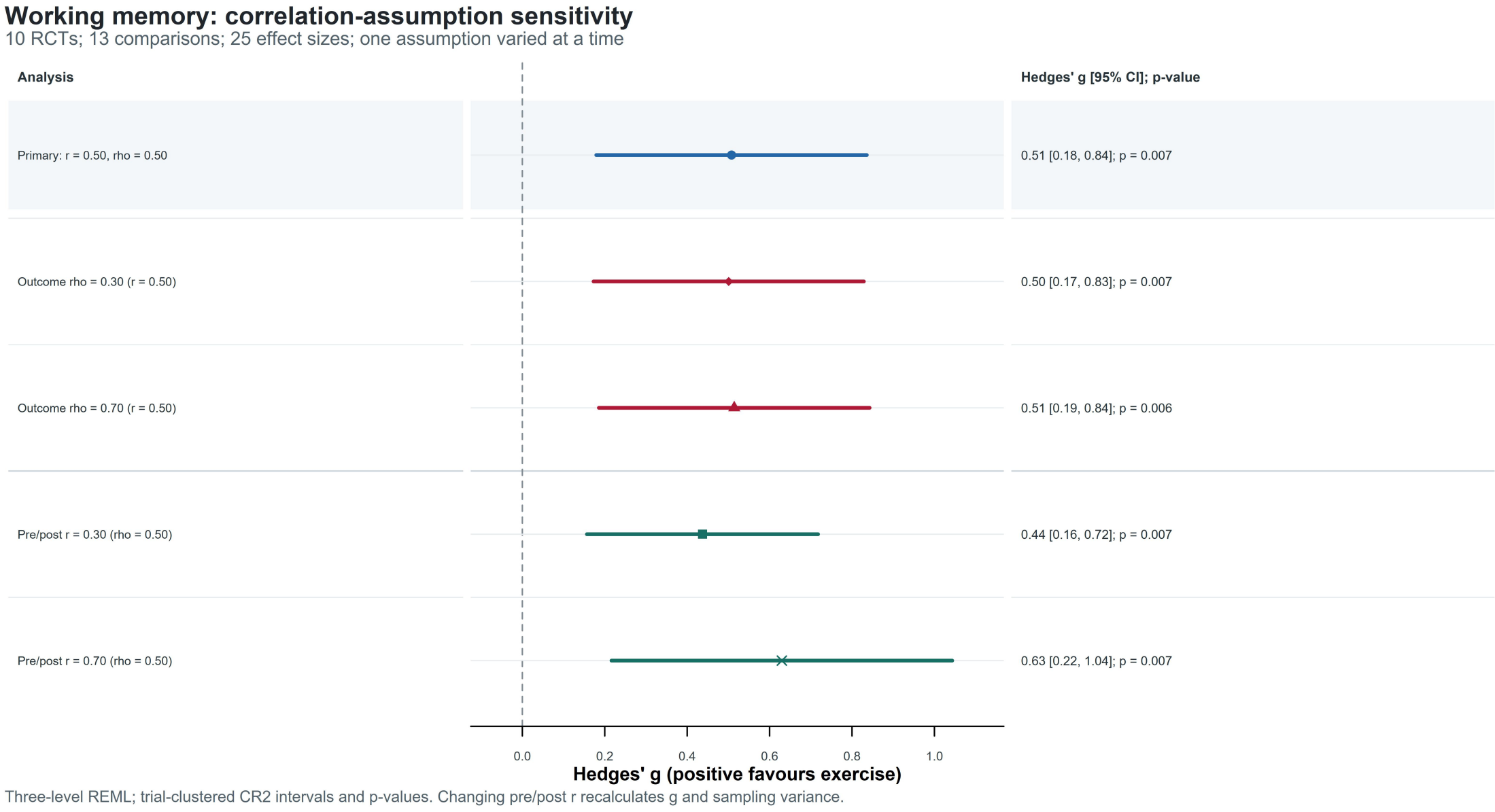

Figure S20. Sensitivity analyses of the pooled effect of exercise on working memory under alternative correlation
assumptions

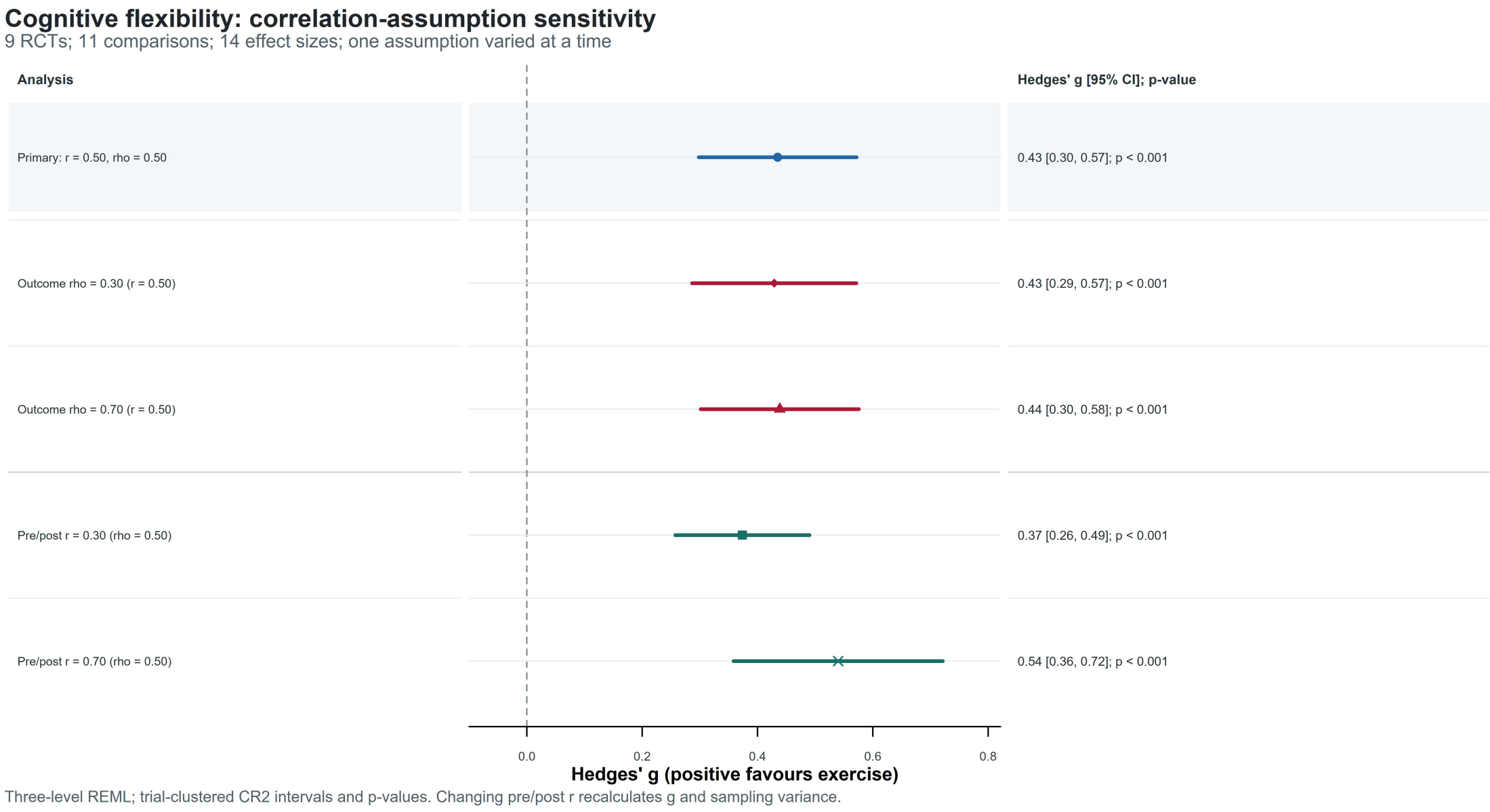

Figure S21. Sensitivity analyses of the pooled effect of exercise on cognitive flexibility under alternative correlation assumptions

7. Publication bias and small-study effects

Publication bias and small-study effects were examined using funnel plots based on trial-level aggregated effect estimates, thereby avoiding the treatment of multiple dependent effect sizes from the same trial as independent observations. Funnel plots were constructed separately for overall executive function and each of the three core executive-function domains. Funnel-plot asymmetry was further assessed using an Egger-type regression test and Begg's rank-correlation test.

For overall executive function, the analysis included 22 independent RCTs. Neither the Egger-type test (p = 0.823) nor Begg's test (p = 0.288) indicated statistically significant funnel-plot asymmetry. Similarly, no statistically significant evidence of small-study effects was detected for inhibitory control based on 17 RCTs (Egger-type p = 0.417; Begg p = 0.440) or for working memory based on 10 RCTs (Egger-type p = 0.484; Begg p = 0.381).

For cognitive flexibility, 9 independent RCTs were available. Neither the Egger-type test (p = 0.770) nor Begg's test (p = 0.477) was statistically significant. However, because fewer than 10 trials contributed to this analysis, the funnel plot and statistical tests for cognitive flexibility were considered exploratory and should be interpreted cautiously because of their limited power to detect asymmetry.

Overall, the funnel plots and formal asymmetry tests provided no statistical evidence of small-study effects across the analysed executive-function outcomes. Nevertheless, these findings should not be interpreted as definitive evidence of the absence of publication bias, particularly for domain-specific analyses with relatively small numbers of trials.

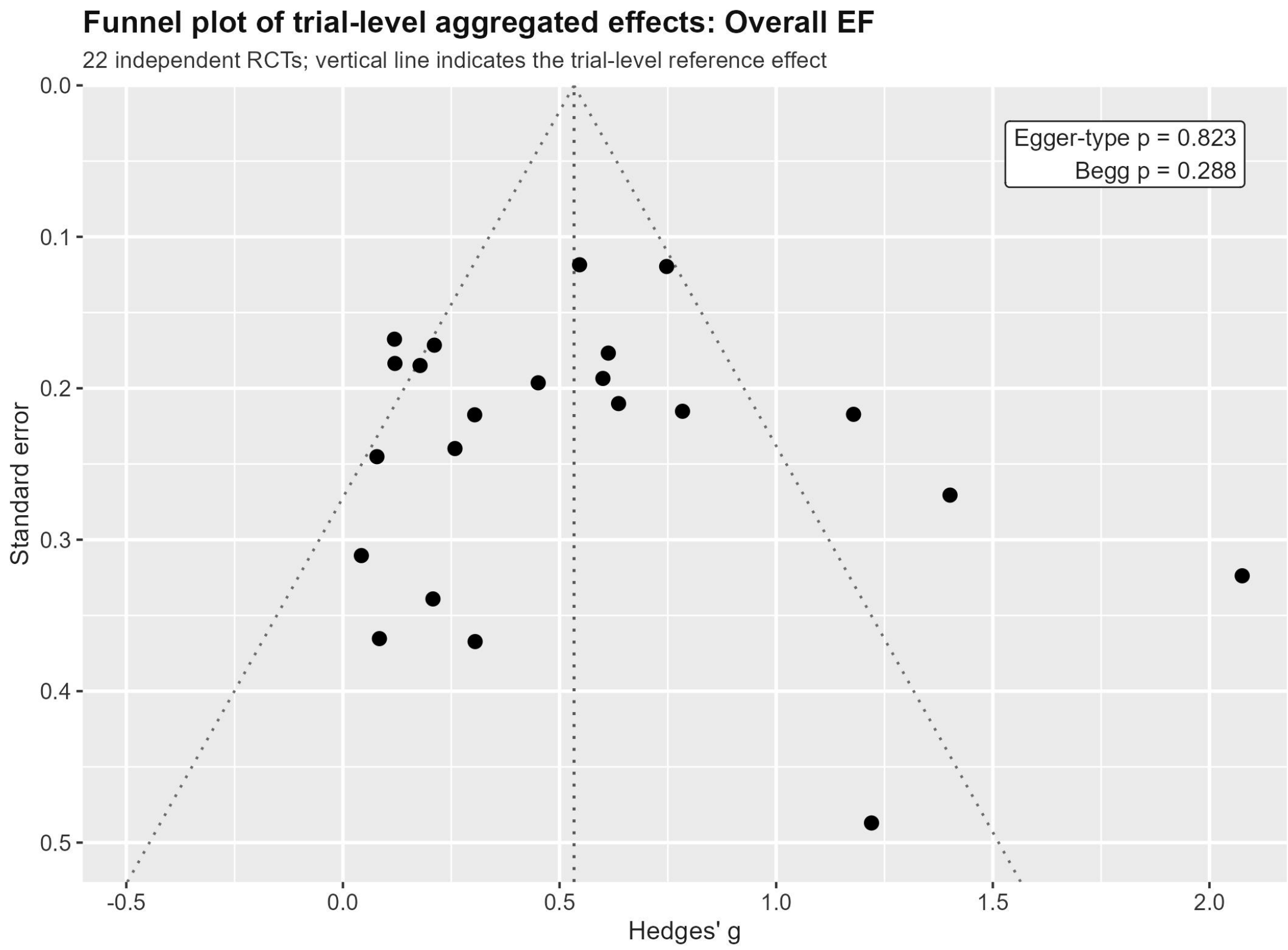

Figure S22. Funnel plot of trial-level aggregated effects for overall executive function.

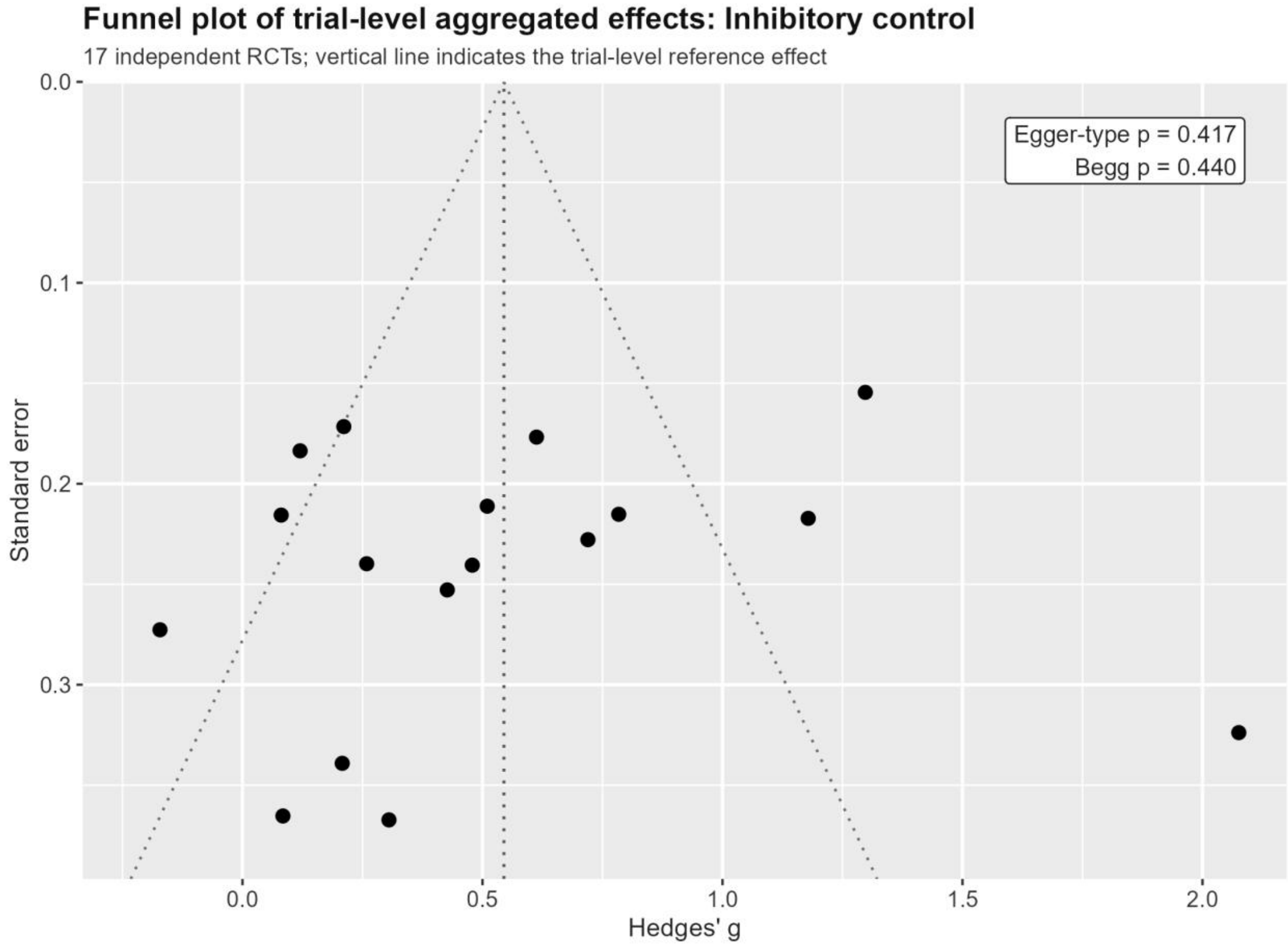

Figure S23. Funnel plot of trial-level aggregated effects for inhibitory control.

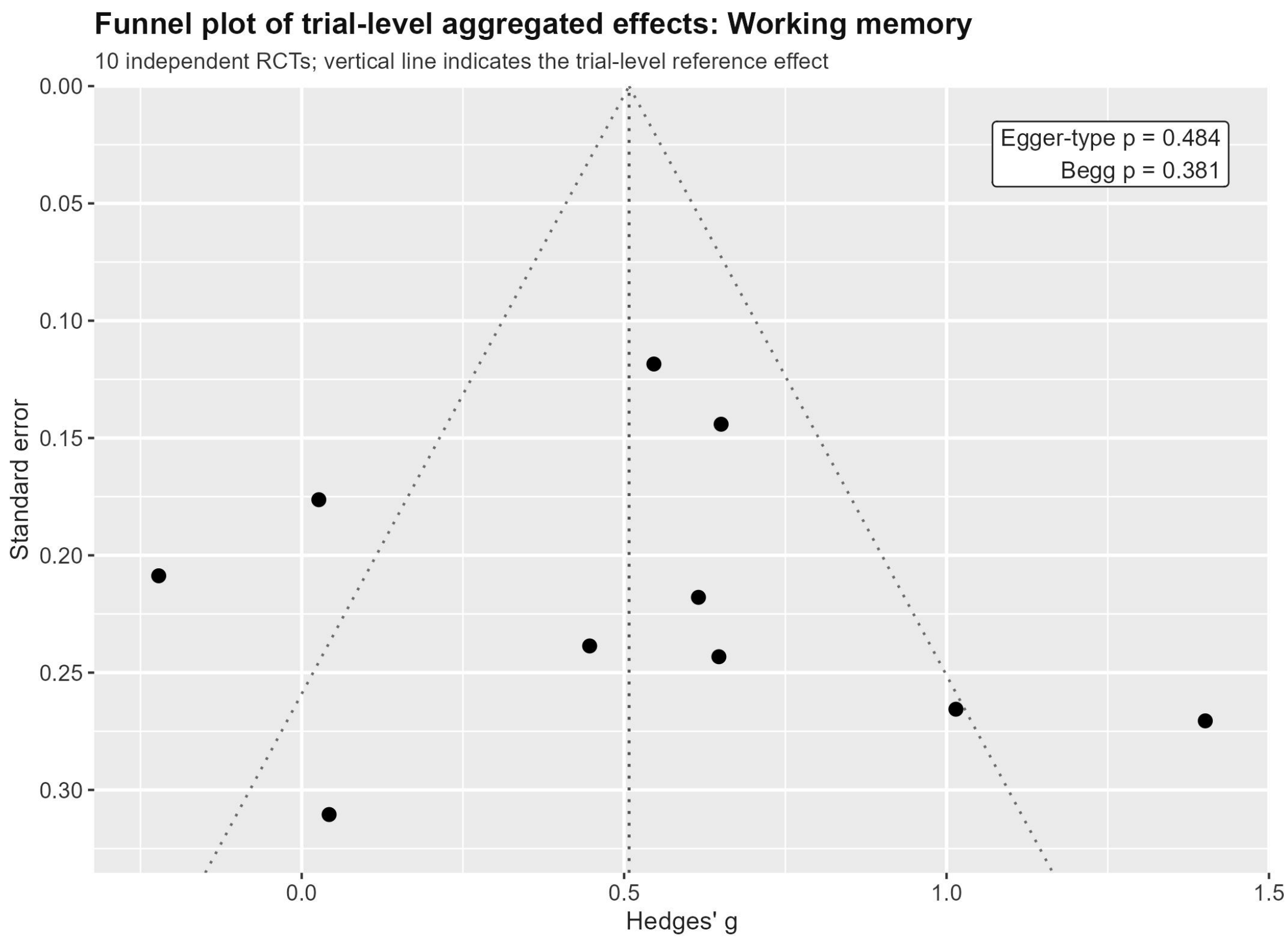

Figure S24. Funnel plot of trial-level aggregated effects for working memory.

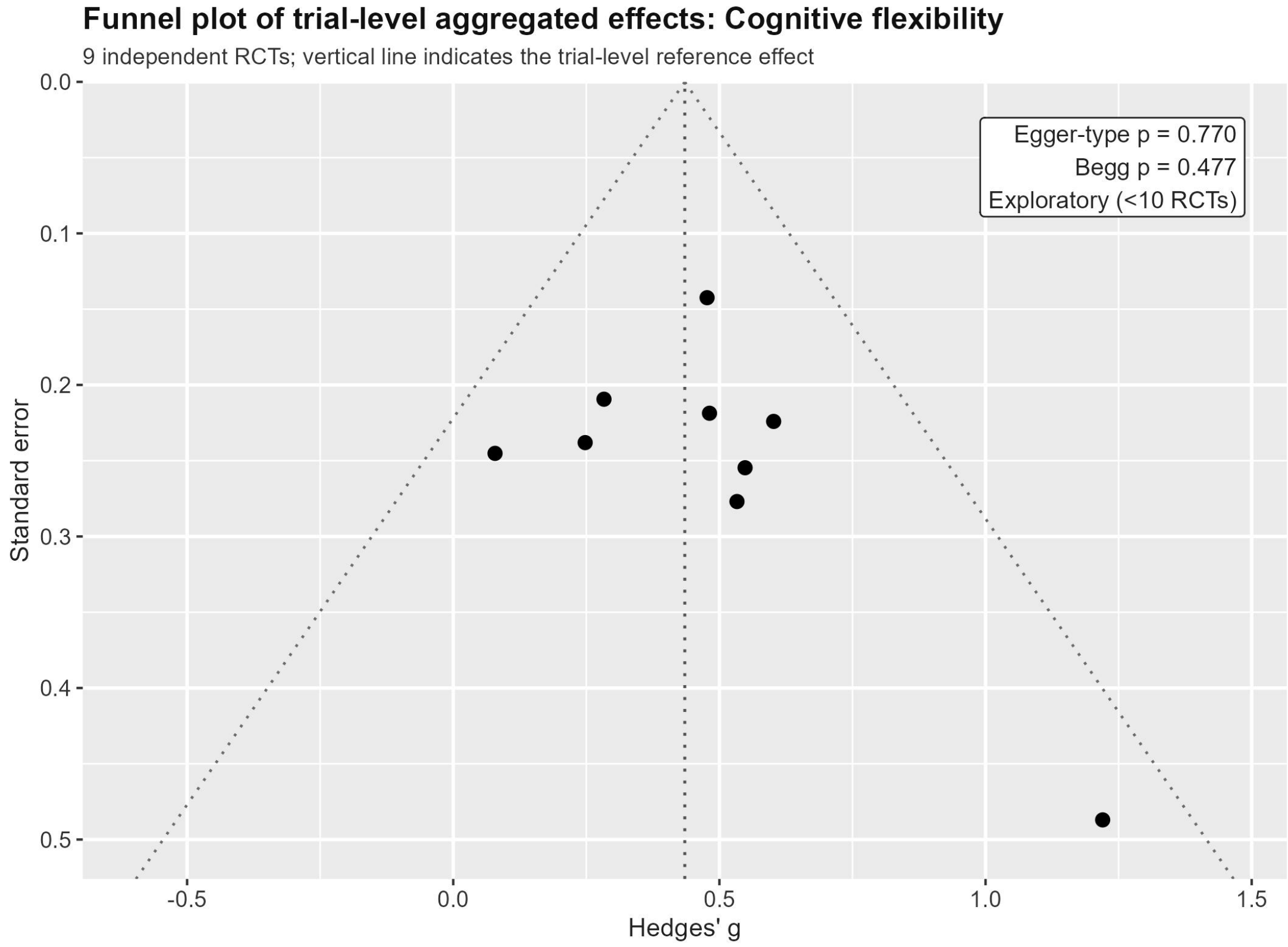

Figure S25. Funnel plot of trial-level aggregated effects for cognitive flexibility.

8. Exploratory meta-regression analyses

Exploratory univariable meta-regression analyses were conducted to examine whether selected continuous study- and intervention-level characteristics were associated with the magnitude of the exercise effect on overall executive function. Four moderators were examined separately: mean participant age, intervention duration, weekly exercise frequency, and session duration. Each moderator was entered into a separate three-level meta-regression model, with trial-clustered CR2 robust inference used for coefficient estimates, confidence intervals, and statistical tests. Studies with missing information for a given moderator were excluded only from the corresponding model. Because four moderator tests were conducted, the resulting p values were additionally adjusted using the Benjamini–Hochberg (BH) procedure.

Mean age showed a negative association with the exercise effect in the unadjusted CR2 analysis ($\beta = -0.102$ per year, 95% CI −0.190 to −0.015, $p = 0.028$), suggesting numerically smaller effects with increasing participant age. However, this association did not remain statistically significant after correction for multiple testing (BH-adjusted $p = 0.111$). Intervention duration was not significantly associated with effect size ($\beta = 0.023$ per week, 95% CI −0.041 to 0.087, $p = 0.165$; BH-adjusted $p = 0.330$). Similarly, no significant associations were observed for weekly exercise frequency ($\beta = -0.032$ per session/week, 95% CI −0.314 to 0.249, $p = 0.795$; BH-adjusted $p = 0.987$) or session duration ($\beta = 0.000$ per minute, 95% CI −0.009 to 0.009, $p = 0.987$; BH-adjusted $p = 0.987$).

Accordingly, none of the four examined moderators provided statistically significant evidence of effect modification after adjustment for multiple testing. These analyses should therefore be interpreted as exploratory, particularly given the limited number of independent trials available for moderator analyses.

Table S12. Exploratory univariable meta-regression analyses for overall executive function.

**Moderator**	**Unit**	**RCTs / comparisons / effects**	**β (95% CI), CR2**	**p, CR2**	**BH-adjusted p**	**τ^2 Level 2 / Level 3**	**Pseudo-R^2 (%)**
Mean age (years)	per 1-year increase	21 / 26 / 83	-0.102 (-0.190 to -0.015)	0.028	0.111	0.051 / 0.114	27.2
Intervention duration (weeks)	per 1-week increase	22 / 27 / 86	0.023 (-0.041 to 0.087)	0.165	0.330	0.049 / 0.084	37.3
Weekly frequency (sessions/week)	per 1-session/week increase	21 / 26 / 80	-0.032 (-0.314 to 0.249)	0.795	0.987	0.043 / 0.183	-7.3
Session duration (min)	per 1-min increase	22 / 27 / 86	0.000 (-0.009 to 0.009)	0.987	0.987	0.050 / 0.175	-5.9

Note. β represents the change in pooled Hedges' g per one-unit increase in the moderator; positive β values indicate a larger exercise-related benefit as the moderator increases. The four raw p-values were adjusted together using the Benjamini–Hochberg procedure. Pseudo-R^2 is the raw proportional change in the summed Level-2 and Level-3 variance relative to an intercept-only model fitted to the same rows; negative values can occur. Moderator values reported as NR were excluded only from the corresponding model. All models used trial-clustered CR2 robust inference.

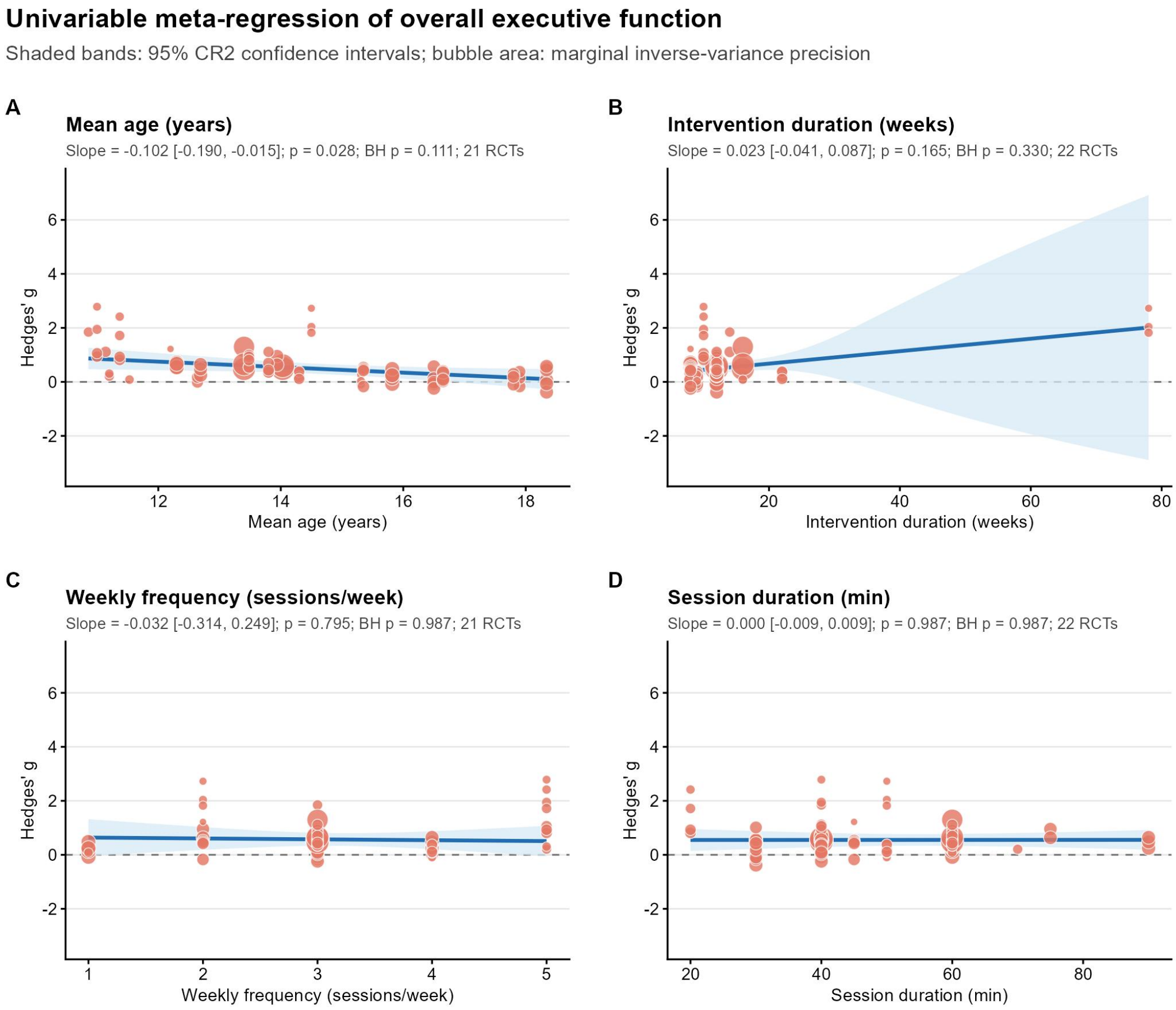

Figure S26. Exploratory univariable meta-regression analyses of moderators for overall executive function.
